# Multi-dimensional structure and subtype-specific heterogeneity of angiogenesis in breast cancer

**DOI:** 10.1371/journal.pone.0348390

**Published:** 2026-10-30

**Authors:** Dennis Baidoo

**Affiliations:** 1 Division of Biostatistics, College of Public Health, The Ohio State University, Columbus, Ohio, United States of America; 2 Department of Mathematics and Statistics, University of New Mexico, Albuquerque, New Mexico, United States of America; 3 Department of Statistics and Actuarial Science, University of Ghana, Accra, Ghana; Michigan Medicine, UNITED STATES OF AMERICA

## Abstract

**Background:**

The prognostic relevance of angiogenesis in breast cancer remains unclear when modeled as a coordinated gene program rather than individual gene effects. This study aimed to characterize the multidimensional structure of angiogenesis and assess the prognostic relevance of its latent components for overall survival in breast cancer.

**Methods:**

Using RNA sequencing data from 1,096 patients with The Cancer Genome Atlas Breast Invasive Carcinoma (TCGA-BRCA) from the United States, we summarized 36 angiogenesis-related genes via principal component analysis (PCA) applied to standardized log-counts per million values. The first three components ($Z_{1}-Z_{3}$), retained based on variance explained, scree plot inspection, and interpretable gene loading patterns, were evaluated for association with overall survival using Kaplan-Meier and Cox proportional hazards models adjusted for age and molecular subtype.

**Results:**

The first three components captured a multi-dimensional structure of angiogenesis activity. None was significantly associated with overall survival in multivariable Cox models (*Z*_1_: HR = 1.016, 95% CI: 0.957–1.078, p = 0.603; *Z*_2_: HR = 1.049, 95% CI: 0.954–1.153, p = 0.319; *Z*_3_: HR = 1.007, 95% CI: 0.918–1.105, p = 0.878), and Kaplan–Meier analyses showed no significant survival differences across median-based groups. Functional enrichment analyses indicated that the retained components represented distinct biological dimensions of angiogenesis. Subtype-specific analyses showed no statistically significant associations within individual subtypes and no strong evidence of effect modification. However, marked differences in latent component distributions across molecular subtypes revealed substantial subtype-specific heterogeneity. Collectively, these findings indicate limited independent prognostic relevance for overall survival despite substantial biological heterogeneity and subtype-specific variation.

**Conclusions:**

Angiogenesis is a multi-dimensional and biologically heterogeneous process. No latent angiogenesis component demonstrated independent prognostic value for overall survival, indicating that angiogenesis is insufficient as a standalone prognostic biomarker. Nevertheless, subtype-specific variation across latent dimensions highlights its relevance for understanding biological heterogeneity in breast cancer.

## Introduction

Do coordinated biological pathways provide meaningful and consistent prognostic information for cancer survival? There have been critical improvements and advances in high-throughput sequencing technologies for breast cancer research over two decades, enhancing a very comprehensive profiling of tumor gene expression at an unprecedented scale [1–6]. Large-scale initiatives such as The Cancer Genome Atlas (TCGA) have provided thousands of tumor samples with linked molecular and clinical data, providing new avenues and opportunities to comprehend the biological mechanisms governing disease progression and survival [7–12].

However, in the abundance of genomic data, a significant amount of the existing literature has focused and contributed primary on identifying individual genes or constructing prognostic signatures associated with survival outcomes [13–16]. While some of these studies have produced valuable genomic insights, they often treat genes independently or solely rely on two-stage analytical techniques that may not fully account for biological coordination processes or associated measurement error. Prior studies have established that cancer progression is rarely driven by isolated genes but rather, the reflection of the activity of a complex biological program, specifically, groups of genes concerned with the regulation of such processes, including angiogenesis, immune evasion, and metastasis [12,17–20]. While multiple biological processes contribute to cancer progression, angiogenesis is uniquely suited for this analysis due to its inherently coordinated and multi-gene regulatory structure. Unlike processes that may be driven by discrete signaling events, angiogenesis involves tightly regulated interactions among vascular, stromal, and tumor-derived factors [21], making it well-suited for a latent, multi-dimensional modeling approach [22].

However, conventional approaches that summarize pathway activity using a single aggregate score or focus on individual gene effects may obscure underlying biological heterogeneity. This pathway-oriented perspective is consistent with modern biological frameworks that emphasize coordinated cellular programs rather than isolated gene effects in cancer development and progression [23–25]. Such methods implicitly assume that all genes within a pathway contribute uniformly to a single dominant signal, which may not reflect the coordinated but diverse roles of genes involved in complex processes like angiogenesis. In contrast, latent variable approaches such as principal component analysis (PCA) [26–28] allow for the decomposition of gene expression into multiple orthogonal dimensions, each capturing distinct patterns of coordinated activity. This framework can reveal biologically meaningful substructures within a pathway (Fig 1), capturing coordinated gene expression while reducing measurement noise and dimensionality, which are common challenges in high-dimensional genomic data. By modeling shared variation across genes, these methods can improve interpretability and stability compared to approaches that rely on individual gene effects or single aggregated pathway scores, enabling the identification of specific transcriptional programs that may differentially relate to clinical outcomes. Several pathway-level and latent modeling approaches have previously been applied to cancer genomics, including pathway dependency models, latent factor methods, and integrative gene expression frameworks [18,22,29]. These approaches demonstrate the value of modeling coordinated biological activity rather than individual genes; however, relatively few studies have applied such frameworks specifically to angiogenesis in breast cancer while simultaneously evaluating survival outcomes, subtype heterogeneity, and clinical covariates. Dimension-reduction methods such as PCA are widely used for identifying dominant patterns in high-dimensional genomic data and provide a natural framework for summarizing coordinated biological activity while limiting redundancy among correlated features [26–28].

**Fig 1:**
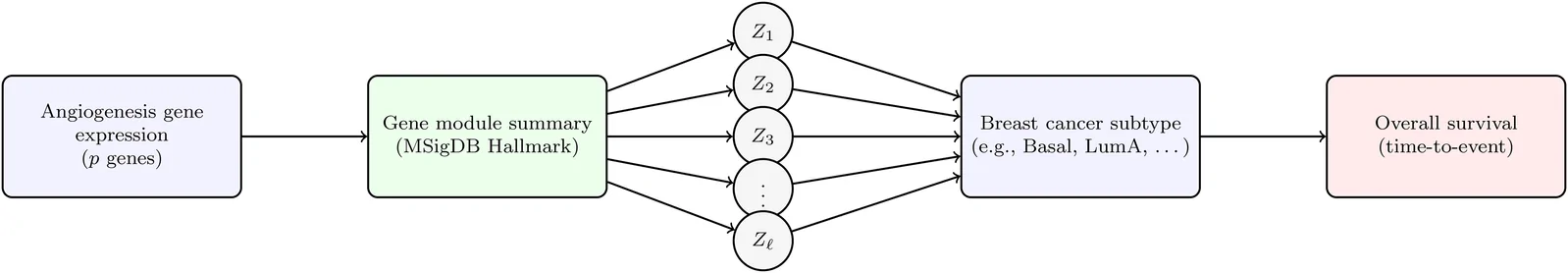
Conceptual framework for modeling angiogenesis as a multi-dimensional latent process. Gene expression is summarized into latent components that capture coordinated biological activity, which can be compared across molecular subtypes and evaluated for association with survival.

Angiogenesis, the formation of new blood vessels, plays a central role in tumor growth and dissemination [21,30–32]. These processes support the promotion of the circulation of oxygen and nutrient delivery to malignant tissue, where angiogenic signaling significantly contributes to aggressiveness and poorer clinical outcomes. Although prior studies have examined associations between angiogenesis-related genes and survival in breast cancer, fewer have modeled angiogenesis as a coherent latent biological program while explicitly accounting for clinical confounding and measurement variability in gene expression data [22,29,30,33,34]. Most prior studies have evaluated angiogenesis using individual genes or composite prognostic signatures derived from selected angiogenesis-related markers. While these approaches have reported associations with clinical outcomes, they generally treat angiogenesis as a single aggregate process and do not explicitly account for coordinated gene expression structure or latent biological heterogeneity within the pathway [33,35,36]. Recent studies have continued to identify angiogenesis-related gene signatures associated with breast cancer prognosis, immune infiltration, and treatment response, further supporting the clinical importance of angiogenic signaling while also highlighting substantial heterogeneity across tumor subtypes [33,35–38]. Consequently, potentially important subtype-specific or functionally distinct dimensions of angiogenesis activity may remain obscured.

Despite these advances, an important gap remains in the literature. Most prior studies have evaluated angiogenesis using single-gene markers or aggregated pathway scores, without explicitly modeling the multi-dimensional structure of gene co-expression or examining whether distinct latent components exhibit differential prognostic relevance across tumor subtypes. Furthermore, limited attention has been given to whether the prognostic contribution of angiogenesis persists after adjustment for key clinical covariates or varies across biologically defined subgroups. Addressing these limitations requires a framework that simultaneously captures latent biological heterogeneity, accounts for confounding, and evaluates subtype-specific effects.

This study investigates the relationship between angiogenesis gene program activity and overall survival in breast cancer using a latent variable framework. Fig 1 illustrates the conceptual framework underlying our analysis. Gene expression measurements from angiogenesis-related genes are first summarized into latent components using principal component analysis (PCA), capturing coordinated biological activity across multiple dimensions rather than a single aggregated signal. These latent components represent distinct transcriptional programs within the angiogenesis pathway and can vary systematically by molecular subtype, reflecting differences in tumor biology and microenvironmental context [11,26].

We then evaluate whether these latent dimensions are associated with overall survival after accounting for established clinical factors. Importantly, the goal is not to claim that any single component is inherently prognostic, but to test whether particular angiogenesis-related transcriptional patterns carry information beyond subtype and other covariates. By integrating biological structure with survival modeling, this work clarifies how angiogenesis-related heterogeneity is organized in TCGA-BRCA and assesses whether that heterogeneity translates into measurable prognostic signal. This approach is particularly appropriate for angiogenesis because it allows multiple independent patterns of coordinated gene expression to be identified, rather than imposing a single summary measure that may obscure biologically distinct signals. We hypothesized that angiogenesis exhibits a multidimensional transcriptional structure and evaluated whether these latent dimensions contribute prognostic information beyond established clinical variables.

## Methods and materials

### Data source

Breast cancer data were obtained from The Cancer Genome Atlas Breast Invasive Carcinoma (TCGA-BRCA) cohort, United States [39]. RNA sequencing (RNA-seq) gene expression profiles and corresponding clinical annotations were downloaded using the TCGAbiolinks package in R [39,40]. The dataset included 1,096 primary breast tumor samples with available overall survival information. For each patient, gene expression was quantified using STAR-aligned RNA sequencing data, resulting in gene-level count measurements across 60,660 genes. All analyses were conducted in R (version 4.5.2). Survival analyses were performed using the survival package, with additional data processing and visualization carried out using standard R packages.

### Ethics approval and consent to participate

This study utilized publicly available, de-identified data from The Cancer Genome Atlas (TCGA) Breast Invasive Carcinoma (TCGA-BRCA) project. The original data were collected under institutional ethical approvals and informed consent procedures by participating institutions. Because the data used in this study were publicly available and de-identified, additional institutional review board approval and informed consent were not required for this secondary analysis. The original data can be found here: (https://portal.gdc.cancer.gov/projects/TCGA-BRCA)

### Gene expression processing

These data were subsequently normalized using trimmed mean of M-values (TMM) and transformed to log-counts per million (log-CPM) prior to standardization for downstream analysis. Genes with low expression were filtered prior to analysis to reduce noise, retaining genes with counts per million (CPM) greater than 1 in at least 10% of samples. This step ensured that downstream analyses were restricted to reliably expressed genes. No explicit batch correction was applied, as the TCGA-BRCA data were processed under standardized protocols; quality control checks were performed to ensure data consistency; however, potential residual batch effects cannot be entirely excluded. The Hallmark Angiogenesis gene set was obtained from the Molecular Signatures Database (MSigDB) [25,41]. This predefined gene set was selected to align with widely used angiogenesis-related signatures in prior studies. Thirty-six genes were matched in the TCGA-BRCA dataset and retained for analysis [24,25].

### Clinical variables and outcome definition

Clinical variables included overall survival (OS) time (days to death or last follow-up), vital status (deceased or censored), clinical covariates including age at diagnosis, tumor stage, molecular subtype, and race as a demographic covariate [11]. Molecular subtype classification was based on TCGA-provided PAM50 annotations and was included as a categorical variable in all adjusted survival models. Survival time was defined as the number of days from diagnosis to death or last follow-up. A complete-case analysis was performed by excluding observations with missing survival outcomes or covariates included in the regression models. In addition, event counts were summarized across molecular subtypes to ensure adequate representation within each subgroup for survival modeling.

### Exploratory latent structure assessment

PCA is widely used for dimensionality reduction in high-dimensional genomic data. PCA identifies orthogonal linear combinations of correlated variables and has become a standard approach for extracting latent structure from high-dimensional molecular datasets [26,27]. To characterize the latent structure of angiogenesis activity, PCA was applied to the standardized gene expression matrix. It was used to identify orthogonal components representing dominant patterns of variation in angiogenesis-related gene expression. PCA summarizes correlated gene expression patterns into a smaller number of composite variables (latent components), each representing a distinct axis of coordinated biological variation. This allows complex gene interactions to be captured in a reduced and interpretable form [42,43]. The first three components were retained based on variance explained, scree plot inspection, and interpretability of gene loading patterns.

In assessing the intrinsic dimensionality of the angiogenesis gene program activity, PCA was performed on standardized log-counts per million (log-CPM) expression values. The first three latent components explained 21.78%, 13.27%, and 7.93% of the variance, respectively, highlighting a multi-dimensional structure without a single dominant axis. These retained components were subsequently evaluated for association with overall survival using Cox proportional hazards regression (Table 3).

### Gene module definition

To study coordinated biological activity, we focused on a predefined angiogenesis-related gene set obtained from the Molecular Signatures Database (MSigDB) [25,41]. The selected module consisted of *p* genes associated with vascular development and angiogenic signaling pathways. Only Genes with counts per million (CPM) greater than 1 in at least 10% of samples were retained for analysis. This filtering step reduced noise from lowly expressed genes and ensured that downstream analyses were restricted to reliably expressed angiogenesis-related genes.

### Data structure and notation

To formalize the analysis, we introduce notation describing gene expression and clinical variables. Let i=1,...,n index breast cancer patients from the TCGA-BRCA cohort. For each patient, we observe the following: $G_{i}=(G_{i1},...,G_{ip})$ – standardized log-CPM expression measurements for genes in the Hallmark Angiogenesis set (*p* = 36), $X_{i}\in {\mathbb{R}}^{n\text{x}p}$: clinical covariates (e.g., age, molecular subtype, tumor stage, and race), $T_{i}$: true event time (overall survival), and $C_{i}$: censoring time.

### Multi-factor latent gene module model

We represent angiogenesis as a multi-dimensional latent process derived from gene expression data. Since our PCA analysis indicated a multi-dimensional structure of the angiogenesis gene program, we let


$$\begin{array}{c} {𝐙}_{i}=(Z_{i1},Z_{i2},Z_{i3}{)}^{⊤} \end{array}$$

denote the patient-specific scores corresponding to the first three principal components of the standardized gene expression matrix. These latent scores can be interpreted as patient-specific measures of underlying angiogenesis activity along distinct biological dimensions, rather than direct measurements of individual genes [26,27].

Let ${𝐆}_{i}=(G_{i1},...,G_{ip}{)}^{⊤}$ denote the standardized expression levels for the *p* angiogenesis-related genes for patient *i*. PCA decomposes the gene expression matrix as


$$\begin{array}{c} G_{i}=\Lambda Z_{i}+\epsilon_{i}, \end{array}$$

where $Z_{i}\in {\mathbb{R}}^{k}$ is the vector of latent components (principal component scores), $\Lambda \in {\mathbb{R}}^{p\times k}$ is the loading matrix, and $\epsilon_{i}\in {\mathbb{R}}^{p}$ is the residual term representing variation not captured by the retained components with mean zero (0) and standard deviation σ. Where $\epsilon_{i}$ captures the remaining unexplained variation. The principal components were obtained by eigen-decomposition of the sample covariance matrix of the standardized gene expression data [44,45]. These components provide a low-dimensional representation of coordinated angiogenesis-related transcriptional activity, capturing dominant patterns of variation across patients, with $Z_{1},Z_{2},$ and *Z*_3_ as the first three principal components.

### Survival analysis

The association between latent angiogenesis activity and overall survival was evaluated using Cox proportional hazards models [46]. Let $T_{i}$ denote the survival time and $\delta_{i}$ the event indicator for patient *i*. The hazard function was specified as


$$\begin{array}{c} \lambda (t|{𝐙}_{i},X_{i})=\lambda_{0}(t)\exp\left(\beta^{⊤}{𝐙}_{i}+\gamma^{⊤}X_{i}\right), \end{array}$$

where $\lambda_{0}(t)$ is the baseline hazard function, $\beta \in {\mathbb{R}}^{k}$ is the vector of regression coefficients for the latent component scores ${𝐙}_{i}$, and γ is the vector of regression coefficients for the clinical covariates $X_{i}$ (age and molecular subtype in the primary multivariable model). Hazard ratios (HRs) were reported as $\exp(\beta_{k})$ for each latent component. In this framework, hazard ratios quantify how changes in latent angiogenesis activity are associated with the relative risk of death, after accounting for clinical covariates. Both univariable and multivariable models were fitted, with the multivariable model adjusting for age and molecular subtype. Tumor stage and race were summarized descriptively and evaluated in exploratory analyses but were not included in the primary multivariable model because the primary objective was to assess whether angiogenesis-related latent structure provided prognostic information beyond age and molecular subtype. No causal interpretation is assumed; the estimated associations reflect conditional relationships between angiogenesis activity and survival under the proportional hazards assumption. The proportional hazards assumption was assessed using Schoenfeld residuals for individual covariates and globally [47,48]. For variables exhibiting evidence of violation, time-dependent effects were explored to evaluate the robustness of model estimates.

## Results

### Baseline characteristics

A total of 1,096 patients with available gene expression and clinical data were included in the analysis, with event counts summarized in Table 1 and 2. For each latent angiogenesis component, patients were stratified into “low” and “high” groups using a median split. Specifically, individuals with component values less than or equal to the cohort median were classified as “low,” while those with values greater than the median were classified as “high.” This approach was adopted to facilitate group-based comparisons in Kaplan–Meier survival analyses [49] and descriptive summaries, while maintaining balanced group sizes and avoiding arbitrary threshold selection. The median split provides a data-driven and reproducible approach for visualization and descriptive survival comparisons. However, dichotomization of continuous variables can reduce statistical power and obscure continuous risk gradients; therefore, primary inference relied on continuous Cox proportional hazards models [50,51].

**Table 1:** Baseline demographic and clinical characteristics of TCGA-BRCA patients stratified by latent angiogenesis components.

Characteristic	Low	High	*p*-value
**(A) *Z***_**1**_ **Stratification**			
N	548	548	
Age (years), mean (SD)	59.4 (12.9)	57.4 (13.3)	0.0091
Overall survival (years), mean (SD)	3.47 (3.45)	3.25 (3.04)	0.2729
Survival status, n (%)			0.8616
Alive, n (%)	473 (86.3)	470 (85.8)	
Dead, n (%)	75 (13.7)	78 (14.2)	
***Subtype***			< 0.001
Luminal A, n (%)	240 (43.8)	328 (59.9)	
Luminal B, n (%)	130 (23.7)	79 (14.4)	
Basal, n (%)	132 (24.1)	65 (11.9)	
HER2-enriched, n (%)	32 (5.8)	50 (9.1)	
***Race***			< 0.001
White, n (%)	343 (62.6)	415 (75.7)	
Black, n (%)	122 (22.3)	60 (10.9)	
Asian, n (%)	33 (6.0)	28 (5.1)	
**(B) *Z***_**2**_ **Stratification**			
N	548	548	
Age (years), mean (SD)	58.7 (13.2)	58.2 (13.1)	0.5051
Overall survival (years), mean (SD)	3.30 (3.22)	3.42 (3.29)	0.5672
Survival status, n (%)			0.1632
Alive, n (%)	480 (87.6)	463 (84.5)	
Dead, n (%)	68 (12.4)	85 (15.5)	
***Subtype***			< 0.001
Luminal A, n (%)	308 (56.2)	260 (47.4)	
Luminal B, n (%)	74 (13.5)	135 (24.6)	
Basal, n (%)	102 (18.6)	95 (17.3)	
HER2-enriched, n (%)	26 (4.7)	56 (10.2)	
***Race***			< 0.001
White, n (%)	379 (69.2)	379 (69.2)	
Black, n (%)	118 (21.5)	64 (11.7)	
**(C) *Z***_**3**_ **Stratification**			
N	548	548	
Age (years), mean (SD)	59.0 (13.5)	57.9 (12.8)	0.1663
Overall survival (years), mean (SD)	3.28 (3.13)	3.44 (3.37)	0.3886
Survival status, n (%)			0.1632
Alive, n (%)	480 (87.6)	463 (84.5)	
Dead, n (%)	68 (12.4)	85 (15.5)	
***Subtype***			< 0.001
Luminal A, n (%)	378 (69.0)	190 (34.7)	
Luminal B, n (%)	145 (26.5)	64 (11.7)	
Basal, n (%)	2 (0.4)	195 (35.6)	
HER2-enriched, n (%)	19 (3.5)	63 (11.5)	
***Race***			0.0010
White, n (%)	379 (69.2)	379 (69.2)	
Black, n (%)	74 (13.5)	108 (19.7)	

**Table 2:** Detailed pathologic stage distribution across latent angiogenesis components.

Component	Stage	Low n (%)	High n (%)	*p*-value
***Z***_**1**_ **Stratification**				0.1985
	Stage IIA	186 (33.9)	151 (27.6)	
	Stage IIB	117 (21.4)	115 (21.0)	
	Stage IIIA	57 (10.4)	80 (14.6)	
	Stage IA	45 (8.2)	42 (7.7)	
	Stage I	32 (5.8)	51 (9.3)	
	Stage IIIC	29 (5.3)	26 (4.7)	
	Stage IIIB	10 (1.8)	12 (2.2)	
	Stage IV	6 (1.1)	11 (2.0)	
	Unknown	53 (9.7)	50 (9.1)	
***Z***_**2**_ **Stratification**				0.0496
	Stage IIA	164 (29.9)	173 (31.6)	
	Stage IIB	120 (21.9)	112 (20.4)	
	Stage IIIA	74 (13.5)	63 (11.5)	
	Stage IA	45 (8.2)	42 (7.7)	
	Stage I	33 (6.0)	50 (9.1)	
	Stage IIIC	29 (5.3)	26 (4.7)	
	Stage IIIB	12 (2.2)	10 (1.8)	
	Stage IV	9 (1.6)	8 (1.5)	
	Unknown	50 (9.1)	53 (9.7)	
***Z***_**3**_ **Stratification**				0.0431
	Stage IIA	153 (27.9)	184 (33.6)	
	Stage IIB	131 (23.9)	101 (18.4)	
	Stage IIIA	78 (14.2)	59 (10.8)	
	Stage IA	40 (7.3)	47 (8.6)	
	Stage I	36 (6.6)	47 (8.6)	
	Stage IIIC	27 (4.9)	28 (5.1)	
	Stage IIIB	15 (2.7)	7 (1.3)	
	Stage IV	5 (0.9)	12 (2.2)	
	Unknown	48 (8.8)	55 (10.0)	

The baseline demographic and clinical characteristics stratified by angiogenesis activity are presented in Table 1. It provides detailed summaries across all latent components, including age, survival outcomes, subtype distribution, race, and tumor stage comparisons between low and high angiogenesis groups. The low and high angiogenesis groups were generally comparable with respect to age, follow-up time, and survival status (Table 1).

In contrast, tumor subtype distribution differed significantly between low and high groups across all stratifications (Table 1; *p* < 0.001 for each comparison). The direction of enrichment depended on the component: high *Z*_1_ was enriched for Luminal A, high *Z*_2_ for Luminal B/HER2-enriched, and high *Z*_3_ for Basal tumors. These subtype-specific differences motivated further investigation of the latent structure of angiogenesis activity and its association with survival outcomes. Significant differences in race distributions were also observed across multiple angiogenesis stratifications (Table 1). For *Z*_1_, the high group contained a greater proportion of White patients and a lower proportion of Black patients than the low group (p < 0.001). Whereas, the high *Z*_3_ group contained a greater proportion of Black patients than the corresponding low group (p = 0.0010). Overall, race distributions differed significantly across several latent angiogenesis components. Detailed distributions of pathologic stage across angiogenesis groups are presented in Table 2. Overall, stage distributions were broadly comparable between low and high groups for most components, with only modest differences observed. Statistically significant differences in stage distribution were observed for the second and third angiogenesis latent components, while the first did not show significant differences. Tumor stage showed limited association with angiogenesis group differences. While stage was not significantly associated with most latent components, modest differences were observed for specific components.

### Association between angiogenesis components and survival

The association between individual angiogenesis principal components and overall survival is presented in Table 3. None of the three components were statistically significantly associated with overall survival, and estimated hazard ratios were close to unity with confidence intervals spanning 1 (*Z*_1_: HR = 1.016, 95% CI: 0.957–1.078, p = 0.603; *Z*_2_: HR = 1.049, 95% CI: 0.954–1.153, p = 0.319; *Z*_3_: HR = 1.007, 95% CI: 0.918–1.105, p = 0.878).

**Table 3:** Association between angiogenesis principal components and overall survival in TCGA-BRCA patients (Cox proportional hazards model).

	Hazard Ratio	95% CI	*z*	*p*-value
*Z*_1_	1.016	0.957–1.078	0.52	0.603
*Z*_2_	1.049	0.954–1.153	1.00	0.319
*Z*_3_	1.007	0.918–1.105	0.15	0.878
*Likelihood ratio test*				0.735

*n* = 1,096 patients; 153 events.

In the Cox model including all three angiogenesis principal components (Table 3), none of the retained components were significantly associated with overall survival, and hazard ratio estimates were close to unity (Concordance index = 0.676, SE = 0.026; likelihood ratio test p = 0.735). Sensitivity analyses for unmeasured confounding yielded E-values of 1.143, 1.276, and 1.091 for *Z*_1_, *Z*_2_, and *Z*_3_, respectively, with corresponding confidence-limit E-values equal to 1. To better characterize the biological interpretation of the latent angiogenesis dimensions, we examined component loadings and downstream functional enrichment patterns. *Z*_2_ was characterized by positive contributions from genes such as *ITGAV*, *OLR1*, *VCAN*, and *PTK2*, which are involved in cell adhesion, extracellular matrix remodeling, and angiogenic signaling. In contrast, negative loadings were observed for genes including *TIMP1*, *JAG2*, and *PDGFA*, which are associated with matrix regulation and signaling pathways involved in vascular stability and remodeling. Functional enrichment results (Fig 2) demonstrated that each latent component represented distinct biological processes, supporting the multidimensional nature of the angiogenesis program.

**Fig 2:**
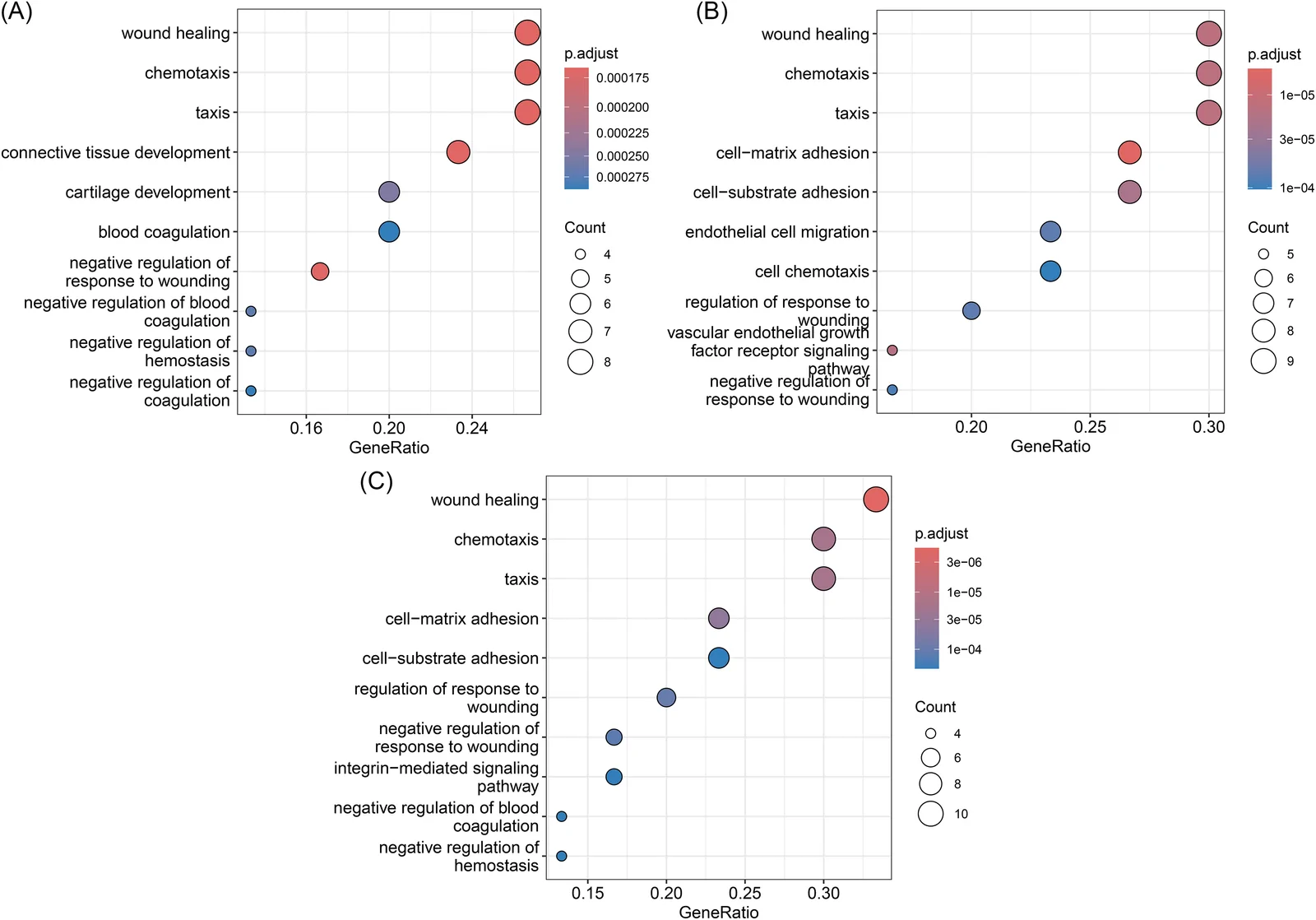
Gene ontology enrichment analysis of latent angiogenesis components. (A) *Z*_1_ is enriched for structural and tissue remodeling processes, including connective tissue development and wound healing. (B) *Z*_2_ is enriched for angiogenesis-related processes such as endothelial cell migration, chemotaxis, and *VEGF* signaling. (C) *Z*_3_ is enriched for regulatory and cell–matrix interaction pathways, including integrin signaling and response to wounding.

Although not associated with survival, *Z*_2_ captured a biologically interpretable gradient involving extracellular matrix remodeling and angiogenic signaling, demonstrating that latent transcriptional programs can be biologically meaningful even when they do not independently predict clinical outcomes. To characterize the biological processes underlying each latent component, gene ontology (GO) enrichment analysis was performed (Fig 2). The first component (*Z*_1_) was primarily enriched for connective tissue development, wound healing, and coagulation. The second component (*Z*_2_) was enriched for endothelial cell migration, chemotaxis, and vascular endothelial growth factor (*VEGF*) receptor signaling. In contrast, the third component (*Z*_3_) was enriched for integrin-mediated signaling, cell–matrix adhesion, and regulation of response to wounding (Fig 2).

The latent components captured distinct angiogenesis-related biological processes. However, none of the individual components showed a statistically significant association with survival in Cox proportional hazards models.

### Latent Structure of Angiogenesis Gene Expression

The transcriptional organization of angiogenesis-related genes is shown in Fig 3. The gene–gene correlation matrix (Fig 3A) contained structured blocks of positive correlations and distinct clusters of co-expressed genes. Hierarchical clustering identified groups of genes with similar expression patterns.

**Fig 3:**
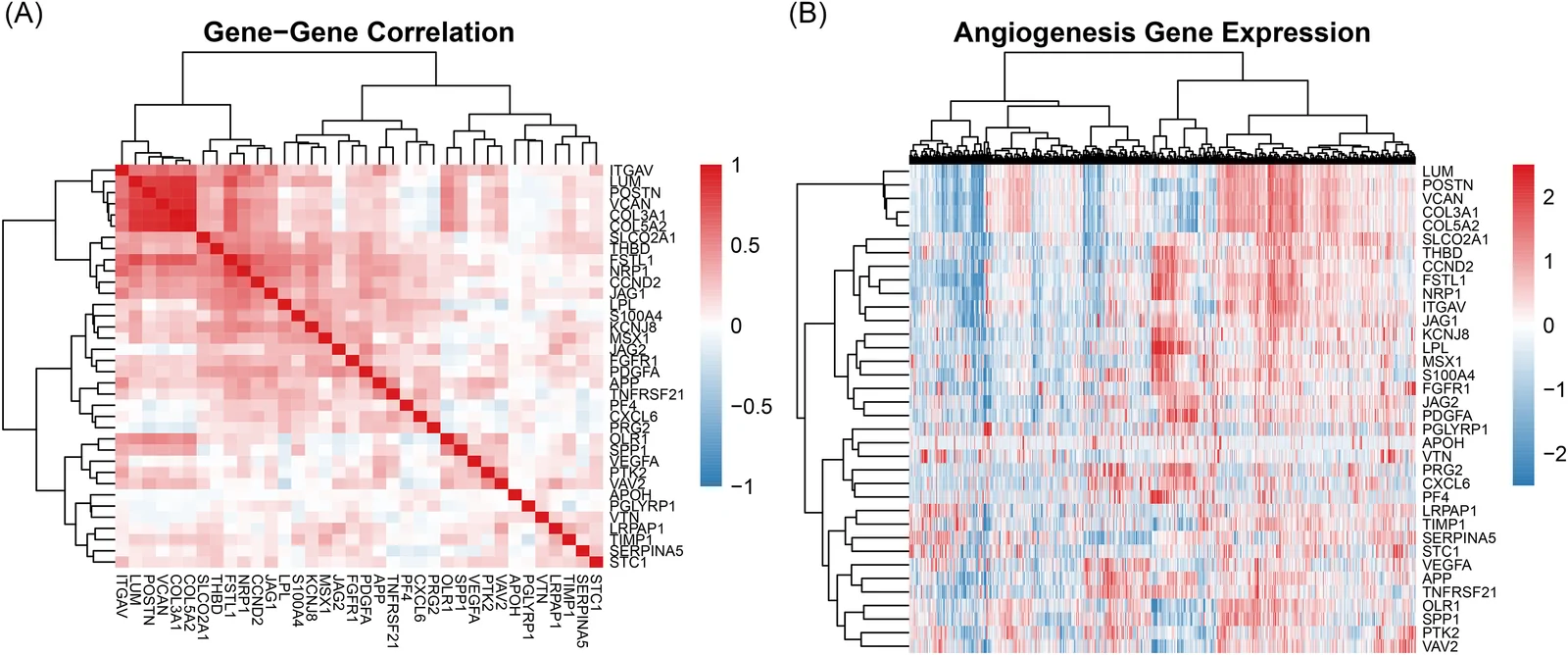
Latent transcriptional organization of the angiogenesis gene program in TCGA-BRCA. (A) Gene–Gene Correlation Structure and (B) Expression Patterns of the Angiogenesis Gene Program in TCGA-BRCA.

Consistent with this modular structure, the gene expression heatmap (Fig 3B) demonstrated substantial heterogeneity across patients, with distinct groups exhibiting coordinated upregulation and downregulation of angiogenesis genes. Distinct patterns were observed across the latent components, with variation distributed across multiple dimensions rather than concentrated within a single component. Distinct clusters of co-expressed genes were observed, with extracellular matrix–related genes such as *COL3A1*, *COL5A2*, and *VCAN* showing coordinated upregulation, while genes such as *VEGFA* and *CXCL6* exhibited variable expression across patients. The correlation matrix and heatmap (Fig 3) revealed substantial modularity in angiogenesis gene expression, providing the biological basis for retaining multiple latent dimensions rather than a single summary score.

### Latent components capture subtype structure and survival heterogeneity

The distributions of latent angiogenesis components across molecular subtypes and survival status are shown in Fig 4 and Table 1. Clear subtype-specific patterns were observed across all three components ($Z_{1}\text{-}Z_{3}$) (Fig 4A). In particular, Basal, Luminal, and Normal subtypes exhibited distinct shifts in component distributions, indicating that angiogenesis-related transcriptional programs vary systematically across molecular subtypes rather than forming a homogeneous signal.

**Fig 4:**
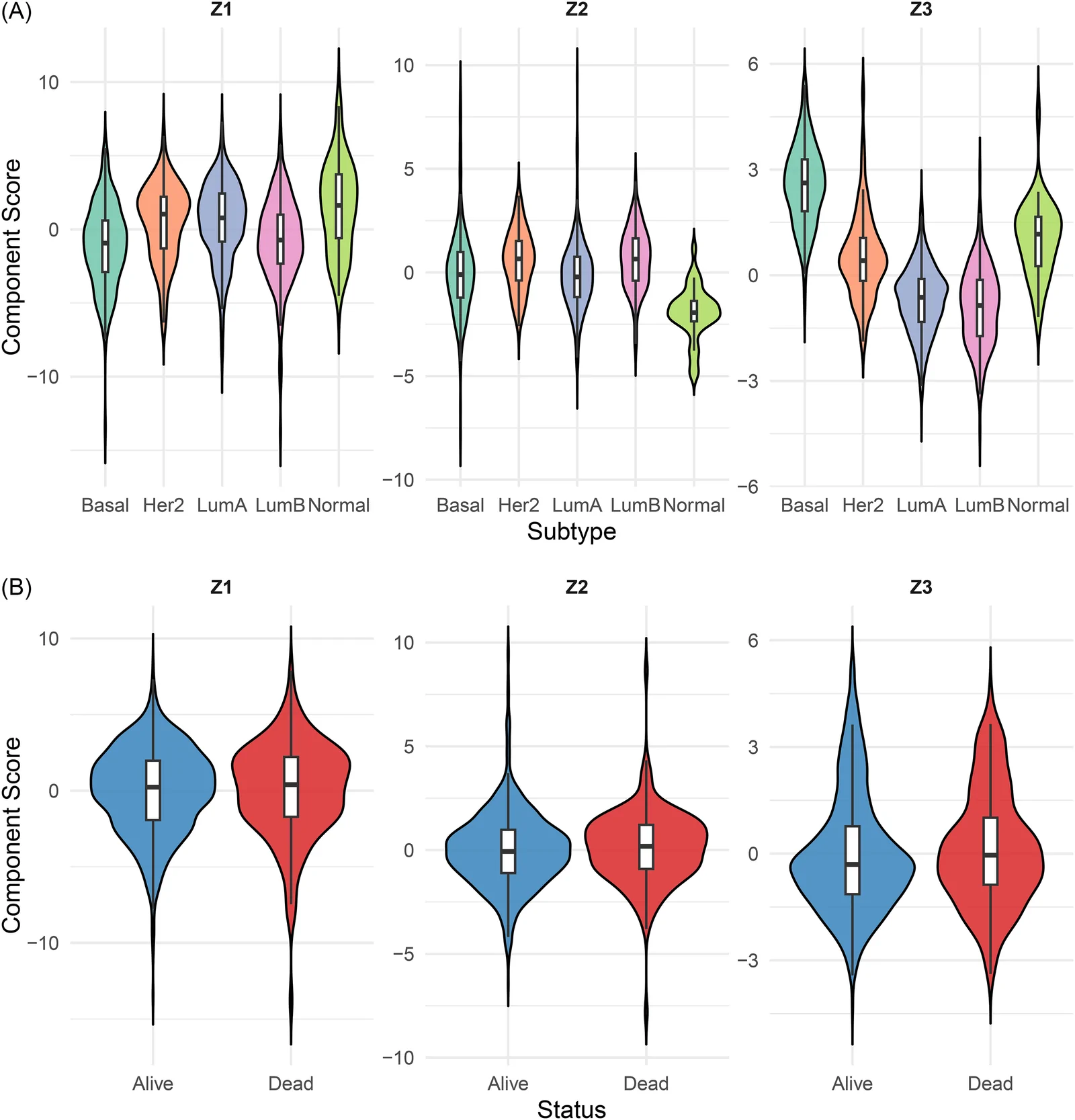
Latent scores correspond to principal component values derived from angiogenesis-related gene expression. (A) Distribution of latent components (*Z*_1_–*Z*_3_) across PAM50 subtypes. (B) Distribution of latent components by survival status (Alive vs Dead). Violin plots were generated using kernel density estimation of the latent scores, with overlaid boxplots indicating the median and interquartile range.

When stratified by survival status (Fig 4B), differences in component distributions between alive and deceased patients were modest. While slight shifts were observed, particularly in *Z*_1_ and *Z*_3_, substantial overlap remained between groups, and survival curves showed only limited separation across component-defined strata.

The latent angiogenesis components captured substantial subtype-related biological variation, whereas associations with survival outcomes were generally modest and distributed across multiple components. To further assess the relationship between angiogenesis activity and survival, Kaplan–Meier and Cox proportional hazards analyses were performed. Fig 4 provides visual evidence that angiogenesis variation is explained largely by molecular subtype rather than survival status.

### Unadjusted and adjusted survival analysis of latent angiogenesis components

Unadjusted Kaplan–Meier survival curves for the latent angiogenesis components are shown in Fig 5. Across all individual components, substantial overlap was observed between the high and low groups, with no statistically significant differences in overall survival detected.

**Fig 5:**
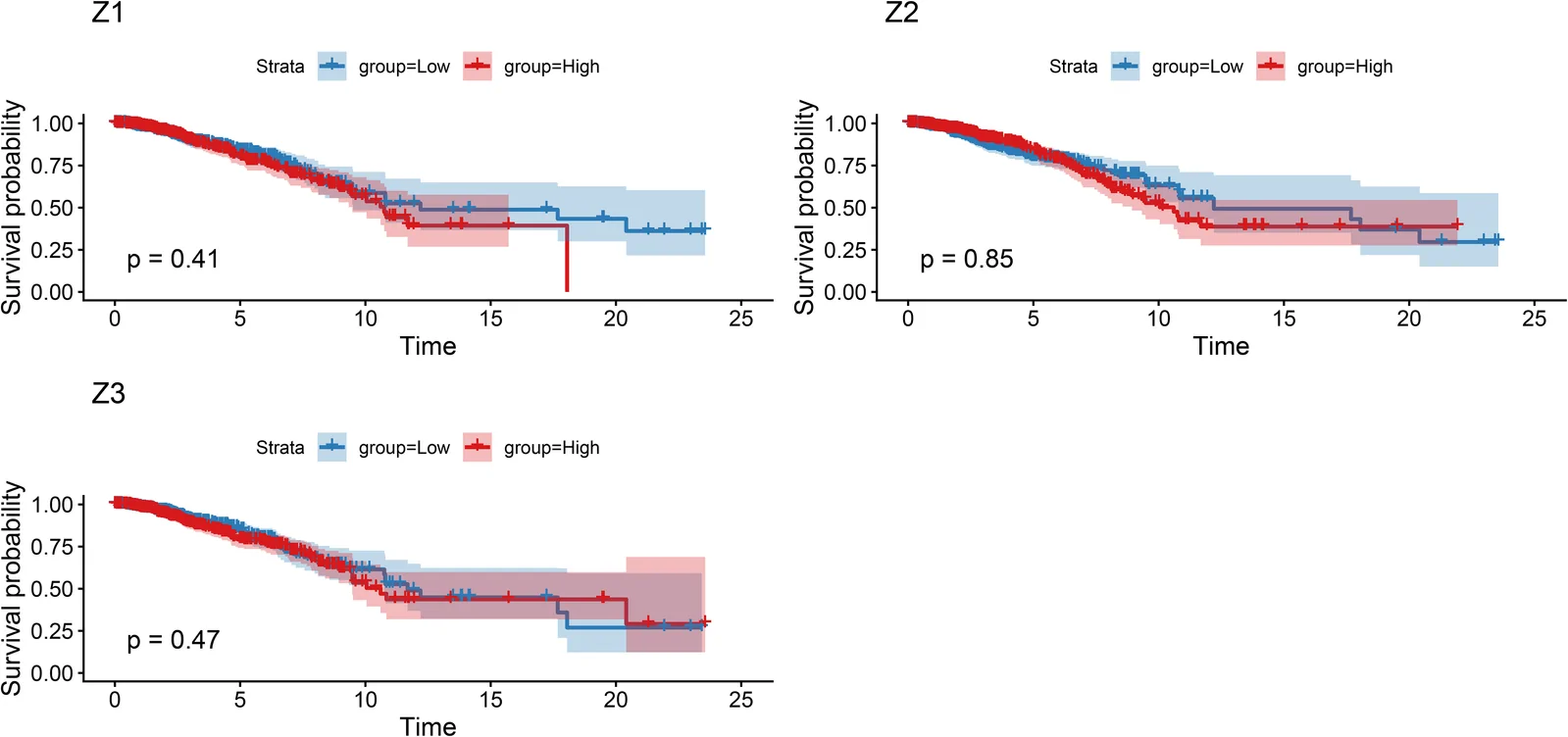
Unadjusted Kaplan–Meier survival curves for latent angiogenesis components. No statistically significant survival differences were observed across the retained latent angiogenesis dimensions $\left(Z_{1}-Z_{3}\right)$. Shaded regions represent 95% confidence intervals.

To assess whether deviations from proportional hazards could explain these patterns, we fitted a time-dependent Cox model for *Z*_3_; the time-dependent term was not statistically significant, and inclusion of this term did not materially alter the estimated associations.

Substantial overlap was observed between survival curves across all components, with limited separation of survival outcomes in the unadjusted analyses. Median-based grouping was used for visualization; however, dichotomization of continuous variables reduces information and may attenuate the ability to detect modest associations. Alternative tertile-based stratification yielded consistent results (log-rank *p* = 0.31). Continuous Cox models likewise did not provide evidence of statistically significant associations for *Z*_1_–*Z*_3_ (Table 3).

Kaplan–Meier survival analyses based on median-split grouping of the latent components are shown in Fig 5. Consistent with these analyses, none of the individual components demonstrated statistically significant differences in survival. Overall, survival curves showed substantial overlap across angiogenesis groups, suggesting limited prognostic separation in this cohort.

Adjusted hazard ratios from the multivariable Cox proportional hazards model are presented in Fig 6. After adjusting for age and molecular subtype, none of the retained latent angiogenesis components were significantly associated with overall survival, with estimated hazard ratios close to unity.

**Fig 6:**
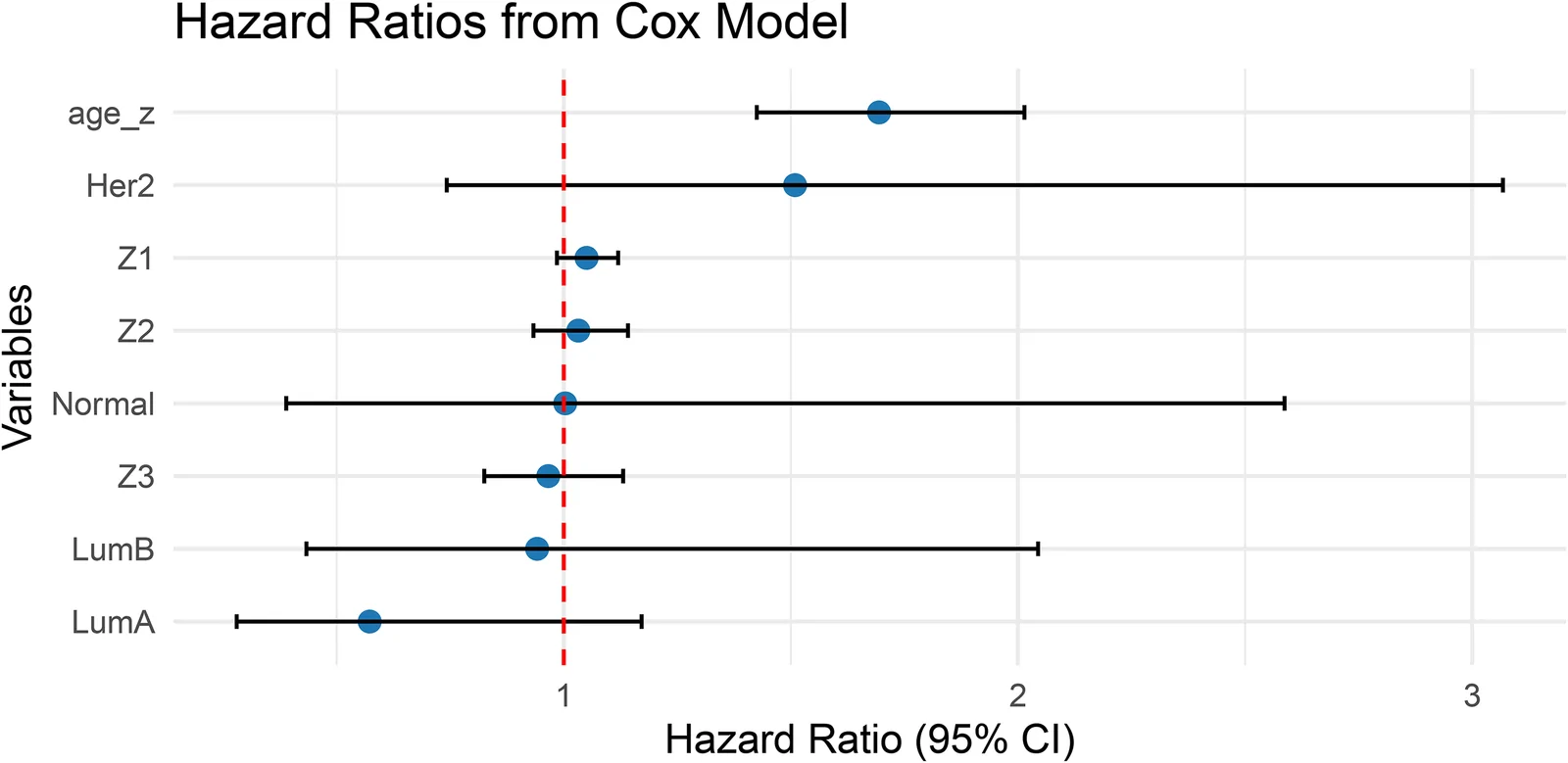
Adjusted hazard ratios for clinical covariates and angiogenesis gene program activity. Hazard ratios and 95% confidence intervals are shown for age at diagnosis and molecular subtypes in a multivariable Cox proportional hazards model. Points represent estimated hazard ratios (HRs), and horizontal lines denote 95% confidence intervals. The dashed vertical line at HR = 1 indicates no association with survival.

In contrast, age remained a statistically significant predictor of survival [52–54]. Molecular subtype also contributed to differences in hazard estimates, although confidence intervals varied across subtype groups. Sensitivity analyses based on proportional hazards diagnostics and time-dependent modeling yielded consistent results, with no material change in the estimated associations. Molecular subtype also demonstrated variability in hazard, although with wider confidence intervals reflecting heterogeneity across groups. Following adjustment for key clinical covariates, the association between angiogenesis-related measures and survival was no longer statistically significant. Across analyses, survival associations were modest, while subtype-specific biological variation accounted for a larger proportion of the observed differences. To further evaluate subtype-specific effects, Cox proportional hazards models were fitted within each molecular subtype. No statistically significant associations between *Z*_2_ and survival were observed across subtypes. In Basal tumors (n = 197, events = 29), the estimated effect was near null (HR = 0.97, 95% CI: 0.80–1.17, p = 0.723). Similarly, no association was observed in HER2 tumors (n = 82, events = 17; HR = 1.01, 95% CI: 0.70–1.44, p = 0.972), Luminal B tumors (n = 209, events = 33; HR = 0.94, 95% CI: 0.74–1.19, p = 0.612), or Normal-like tumors (n = 40, events = 7; HR = 1.45, 95% CI: 0.65–3.21, p = 0.364). In Luminal A tumors (n = 568, events = 67), a modest but non-significant association was observed (HR = 1.10, 95% CI: 0.96–1.27, p = 0.177).

## Discussion

In this study, we examined the prognostic role of angiogenesis in breast cancer using a latent variable framework derived from gene expression data, characterizing angiogenesis as a structured, multi-dimensional process rather than a single pathway measure. Our results show that angiogenesis is not a single homogeneous pathway but a multi-dimensional biological process with subtype-specific variation (Fig 4). However, survival associations were weak and not statistically significant in either the continuous Cox proportional hazards models (Table 3) or the Kaplan–Meier analyses (Fig 5). Together, these findings indicate that angiogenesis-related transcriptional variation in TCGA-BRCA is more strongly associated with tumor subtype than with overall survival.

The observed weak prognostic signal can be explained by the underlying transcriptional structure of angiogenesis [55,56]. Gene correlation and clustering analyses revealed coordinated but distinct expression modules (Fig 3), and PCA confirmed that variation in angiogenesis-related expression is distributed across multiple latent dimensions rather than captured by a single dominant axis (Table 3). For example, *Z*_2_ reflected a balance between extracellular matrix remodeling and regulatory signaling pathways based on gene loadings [57–59]. While these latent axes are biologically interpretable, they did not translate into statistically significant survival associations in this cohort (Table 3), suggesting that angiogenesis-related transcriptional heterogeneity may be more reflective of subtype biology than independent prognosis. These interpretations are based on loading patterns and should be considered exploratory and hypothesis-generating. Differences in race distributions were also observed across several latent angiogenesis stratifications (Table 1). Similar demographic variation has been reported previously in studies describing racial differences in breast cancer subtype composition and clinical presentation [60–62].

These findings further highlight the heterogeneity present within the TCGA-BRCA cohort. After adjusting for age and molecular subtype, none of the retained latent angiogenesis components remained significantly associated with overall survival (Fig 6), and their inclusion did not significantly improve model fit beyond clinical covariates alone (likelihood ratio test, p > 0.05). In contrast, age remained a strong predictor of survival (HR = 1.58, 95% CI: 1.324–1.880, *p* < 0.05). Model diagnostics indicated generally acceptable adherence to the proportional hazards assumption, with no evidence of violation for all covariates. The global test was not statistically significant, and incorporation of a time-dependent effect did not yield a statistically significant association or materially change the study conclusions [63], suggesting that the association is stable across modeling assumptions. While PCA represents a simplified approach, it provides an interpretable framework for identifying dominant patterns of coordinated gene expression. Sensitivity analyses for unmeasured confounding yielded relatively small E-values for all three latent components (*Z*_1_: 1.143, *Z*_2_: 1.276, and *Z*_3_: 1.091), with confidence-limit E-values equal to 1. These findings indicate that only modest unmeasured confounding would be required to explain the observed associations, consistent with the overall weak effect sizes observed across analyses [64].. The concordance index suggested moderate discriminatory ability (C-index = 0.676), indicating that latent angiogenesis patterns captured some information related to survival outcomes. However, the individual latent components were not significantly associated with overall survival (Table 3), and the observed discrimination remained insufficient to support angiogenesis as a robust standalone prognostic biomarker. From a translational perspective, the moderate prognostic discrimination observed in this study suggests that angiogenesis-related transcriptional activity captures some survival-relevant information. However, the lack of statistically significant component-specific associations and the modest magnitude of discrimination indicate that angiogenesis alone is unlikely to provide clinically sufficient risk stratification. Rather than indicating failure, this finding highlights the importance of evaluating biological pathways within integrative frameworks that incorporate multiple components of the tumor microenvironment. These results suggest that angiogenesis contributes only a partial and context-dependent signal, reinforcing the need for multi-factor prognostic models.

Across analyses, estimates for *Z*_2_ were not statistically significant, and Kaplan–Meier analyses based on dichotomized groups similarly showed no clear separation (Fig 5). While dichotomization can reduce power to detect subtle gradients, the current results do not provide strong evidence for a survival association of *Z*_2_ in this cohort.

Importantly, subtype-stratified analyses did not reveal statistically significant associations within individual subtypes, and no strong evidence of interaction was observed. This finding further supports the conclusion that the observed subtype-specific differences in angiogenesis activity do not translate into clinically meaningful differences in overall survival, despite clear subtype separation in the latent score distributions (Fig 4A). This suggests that any prognostic contribution of angiogenesis is modest and not consistently reproducible across molecular context (Table 1–2, and Fig 5). This heterogeneity suggests that angiogenesis primarily reflects subtype-specific biology [21] rather than a universal prognostic mechanism. These findings provide a potential explanation for inconsistent results in prior studies that treated angiogenesis as a single pathway, where some studies report significant associations with survival while others find limited or no prognostic value [33,35–37]. By modeling angiogenesis as a multi-dimensional process, our results suggest that these inconsistencies may arise from collapsing distinct biological signals into a single summary measure [13–16]. These findings are broadly consistent with, but also distinct from, prior studies that have reported stronger prognostic associations for angiogenesis-related gene signatures in breast cancer. Many earlier studies have identified angiogenesis as a significant predictor of survival; however, these analyses often rely on aggregated pathway scores or selected gene signatures, which may capture dominant signals driven by a subset of highly prognostic genes rather than the full complexity of the angiogenesis program. In contrast, the present study models angiogenesis as a multi-dimensional latent process, revealing that angiogenesis-related transcriptional variation is distributed across multiple orthogonal components rather than concentrated in a single dominant signal (Fig 3 and Fig 2). Furthermore, this study did not include direct benchmarking against established angiogenesis scoring methods or previously published prognostic gene signatures. For example, recent angiogenesis-related prognostic signature studies have reported improved survival prediction using selected gene panels [33,35,37]. In contrast, the present analysis suggests that when angiogenesis is modeled as a multidimensional latent program, substantial biological heterogeneity remains but independent prognostic effects are limited (Table 3 and Fig 5).

Differences in study design may further explain these discrepancies [33,35–37]. Prior analyses frequently vary in cohort composition, clinical endpoints (e.g., disease-free vs. overall survival), gene set definitions, and modeling strategies, including the use of dichotomized risk scores or penalized regression approaches that can amplify apparent associations [50,51]. Additionally, subtype composition and imbalance across cohorts may influence reported prognostic effects, particularly if certain subtypes exhibit stronger baseline angiogenic activity (Table 1) [21,53]. By explicitly adjusting for molecular subtype and evaluating interaction effects, our analysis suggests that previously reported associations may partially reflect underlying subtype heterogeneity rather than a consistent, independent prognostic role for angiogenesis. These findings highlight the importance of modeling biological pathways as structured, multi-dimensional systems [13–16] and suggest that angiogenesis alone may have limited utility as a standalone prognostic biomarker.

Subtype-specific patterns may reflect underlying biological heterogeneity and differences in tumor microenvironment rather than direct angiogenesis effects. In our cohort, this is supported by the strong subtype shifts in latent score distributions (Fig 4A) alongside the comparatively weak separation by survival status and Kaplan–Meier curves (Fig 4B and Fig 5). Overall, these results suggest that future prognostic models should integrate angiogenesis with other tumor microenvironmental features to improve predictive performance [65–68].

Despite these findings, several limitations should be considered. First, the analysis is based on observational data from the TCGA-BRCA cohort, which lacks standardized treatment assignment and comprehensive clinical annotations. As a result, treatment-related confounding could not be fully accounted for, and the observed associations should be interpreted as prognostic rather than causal. Additionally, the cohort represents a heterogeneous population collected across multiple centers, which may introduce variability in measurement and clinical management (Table 1 and Table 2). While we adjusted for key covariates such as age and subtype, residual confounding cannot be excluded. Tumor stage was evaluated descriptively and demonstrated only modest differences across latent angiogenesis groups. Additional exploratory stage-adjusted analyses resulted in substantial reductions in sample size and event count because of incomplete stage information, limiting their interpretability. Additionally, the analysis was conducted using a single cohort (TCGA-BRCA), and external validation in independent datasets such as the Molecular Taxonomy of Breast Cancer International Consortium (METABRIC) or other large breast cancer cohorts was not performed. Future studies should evaluate the reproducibility and transportability of latent angiogenesis components across independent populations and clinical settings [69,70]. As such, the generalizability of the findings to other populations and clinical settings remains to be established.

Second, the latent variable framework relies on principal component analysis, which captures linear combinations of gene expression and may not fully represent complex, nonlinear, or context-specific biological interactions. Although sensitivity analyses showed no evidence of strong nonlinearity or time-dependent effects, subtle interactions between angiogenesis and other tumor microenvironmental processes may not be captured in the current model. Furthermore, the use of a predefined angiogenesis gene set may omit relevant pathways or genes that contribute to vascular dynamics, potentially limiting the scope of the analysis [71,72].

Furthermore, the observed patterns highlight the complexity of linking angiogenesis-related transcriptional activity to clinically actionable risk stratification. Although individual components captured biologically distinct transcriptional programs (Fig 2), none demonstrated robust prognostic associations in Cox or Kaplan–Meier analyses (Table 3 and Fig 5), suggesting that angiogenesis-related variation primarily reflects biological heterogeneity rather than survival differences. Emerging evidence suggests that angiogenesis interacts extensively with immune regulation, stromal remodeling, vascular normalization, and myeloid-cell–mediated signaling processes, supporting the need for integrative models that jointly evaluate multiple components of the tumor microenvironment rather than angiogenesis alone [65–68].

However, without independent validation and deeper integration of clinical and biological context, these signals remain insufficient for direct clinical application. Instead, they provide a foundation for future work aimed at integrating angiogenesis with complementary molecular and clinical features to improve prognostic modeling.

## Conclusion

This study demonstrates that angiogenesis in breast cancer exhibits a multi-dimensional transcriptional structure, with substantial subtype-specific variation but no strong independent prognostic effect for overall survival. Across unadjusted, adjusted, and subtype-specific analyses, none of the retained latent components showed statistically significant associations with overall survival.

Multivariable analyses further confirmed the limited independent prognostic value of angiogenesis, with clinical factors such as age contributing more strongly to survival prediction. These findings suggest that angiogenesis primarily reflects subtype-specific biological heterogeneity rather than a stable or universal prognostic mechanism.

Importantly, angiogenesis should not be interpreted as a standalone prognostic biomarker but rather as one component of the broader tumor microenvironment. Future prognostic models may benefit from integrating angiogenesis-related signals with complementary biological processes, including immune activity, stromal composition, hypoxia, and treatment response. In addition, validation in independent cohorts and across diverse clinical settings will be essential to assess the generalizability and potential clinical utility of these findings.

## References

[1] Stover DG, Wagle N. Precision medicine in breast cancer: genes, genomes, and the future of genomically driven treatments. Curr Oncol Rep. 2015;17(4):15. doi: 10.1007/s11912-015-0438-0 25708799 PMC5854397

[2] Karn T. High-throughput gene expression and mutation profiling: current methods and future perspectives. Breast Care. 2013;8(6):401–6. doi: 10.1159/000357461 PMC3919435 24550747

[3] Curtis C. Genomic profiling of breast cancers. Curr Opin Obstet Gynecol. 2015;27(1):34–9. doi: 10.1097/GCO.0000000000000145 25502431 PMC4497788

[4] Orsini A, Diquigiovanni C, Bonora E. Omics Technologies Improving Breast Cancer Research and Diagnostics. International Journal of Molecular Sciences. 2023;24(16):12690. doi: 10.3390/ijms241612690 PMC10454385 37628869

[5] Reis-Filho JS. Next-generation sequencing. Breast Cancer Res. 2009;11 Suppl 3(Suppl 3):S12. doi: 10.1186/bcr2431 20030863 PMC2797692

[6] Amevor R, et al. Integrative prognostic modeling of breast cancer survival with gene expression and clinical data. In: arXiv.org, 2025. https://arxiv.org/abs/2508.16740

[7] Lee J-S. Exploring cancer genomic data from the cancer genome atlas project. BMB Rep. 2016;49(11):607–11. doi: 10.5483/bmbrep.2016.49.11.145 27530686 PMC5346320

[8] Chang JT-H, Lee YM, Huang RS. The impact of the Cancer Genome Atlas on lung cancer. Transl Res. 2015;166(6):568–85. doi: 10.1016/j.trsl.2015.08.001 26318634 PMC4656061

[9] Colaprico A, Silva TC, Olsen C, Garofano L, Cava C, Garolini D, et al. TCGAbiolinks: an R/Bioconductor package for integrative analysis of TCGA data. Nucleic Acids Res. 2016;44(8):e71. doi: 10.1093/nar/gkv1507 26704973 PMC4856967

[10] Liu J, Lichtenberg T, Hoadley KA, Poisson LM, Lazar AJ, Cherniack AD, et al. An Integrated TCGA Pan-Cancer Clinical Data Resource to Drive High-Quality Survival Outcome Analytics. Cell. 2018;173(2):400-416.e11. doi: 10.1016/j.cell.2018.02.052 29625055 PMC6066282

[11] Tomczak K, Czerwińska P, Wiznerowicz M. The Cancer Genome Atlas (TCGA): an immeasurable source of knowledge. Contemp Oncol (Pozn). 2015;19(1A):A68-77. doi: 10.5114/wo.2014.47136 25691825 PMC4322527

[12] Wang Z, Jensen MA, Zenklusen JC. A Practical Guide to The Cancer Genome Atlas (TCGA). Methods Mol Biol. 2016;1418:111–41. doi: 10.1007/978-1-4939-3578-9_6 27008012

[13] West M, Ginsburg GS, Huang AT, Nevins JR. Embracing the complexity of genomic data for personalized medicine. Genome Res. 2006;16(5):559–66. doi: 10.1101/gr.3851306 16651662

[14] Qiu YL, Zheng H, Devos A, Selby H, Gevaert O. A meta-learning approach for genomic survival analysis. Nat Commun. 2020;11(1):6350. doi: 10.1038/s41467-020-20167-3 33311484 PMC7733508

[15] Anaya J, et al. A pan-cancer analysis of prognostic genes. PeerJ. 2016;3:e1499. doi: 10.7717/peerj.1499 PMC4815555 27047702

[16] Zhao Q, et al. Combining multidimensional genomic measurements for predicting cancer prognosis: observations from TCGA. Brief Bioinform. 2014;16(2):291–303. doi: 10.1093/bib/bbu003 PMC4375393 24632304

[17] Creixell P, Reimand J, Haider S, Wu G, Shibata T, Vazquez M, et al. Pathway and network analysis of cancer genomes. Nat Methods. 2015;12(7):615–21. doi: 10.1038/nmeth.3440 26125594 PMC4717906

[18] Edelman EJ, Guinney J, Chi J-T, Febbo PG, Mukherjee S. Modeling cancer progression via pathway dependencies. PLoS Comput Biol. 2008;4(2):e28. doi: 10.1371/journal.pcbi.0040028 18282083 PMC2242820

[19] Xu M, Kao M-CJ, Nunez-Iglesias J, Nevins JR, West M, Zhou XJ. An integrative approach to characterize disease-specific pathways and their coordination: a case study in cancer. BMC Genomics. 2008;9 Suppl 1(Suppl 1):S12. doi: 10.1186/1471-2164-9-S1-S12 18366601 PMC2386054

[20] Chen B, Aouiche C, Shang X. Integrating multiple datasets to discover stage-specific cancer related genes and stage-specific pathways. Bioinformatics research and applications. 2019. p. 240–50. 10.1007/978-3-030-17938-0_22 PMC6509867 31074385

[21] Elayat G, Selim A. Angiogenesis in breast cancer: insights and innovations. Clin Exp Med. 2024;24(1):178. doi: 10.1007/s10238-024-01446-5 39105831 PMC11303414

[22] Merl D, Chen JL-Y, Chi J-T, West M. An integrative analysis of cancer gene expression studies using bayesian latent factor modeling. Ann Appl Stat. 2009;3(4):1675–94. doi: 10.1214/09-aoas261 20953268 PMC2954437

[23] Hanahan D. Hallmarks of cancer: new dimensions. Cancer Discovery. 2022;12(1):31–46. doi: 10.1158/2159-8290.cd-21-1059 35022204

[24] Subramanian A, Tamayo P, Mootha VK, Mukherjee S, Ebert BL, Gillette MA, et al. Gene set enrichment analysis: a knowledge-based approach for interpreting genome-wide expression profiles. Proc Natl Acad Sci U S A. 2005;102(43):15545–50. doi: 10.1073/pnas.0506580102 16199517 PMC1239896

[25] Liberzon A r t h u r, et al. The molecular signatures database hallmark gene set collection. Cell Systems. 2015;1(6):417–25. doi: 10.1016/j.cels.2015.12.004 PMC4707969 26771021

[26] Jolliffe IT, Cadima J. Principal component analysis: a review and recent developments. Philos Trans A Math Phys Eng Sci. 2016;374(2065):20150202. doi: 10.1098/rsta.2015.0202 26953178 PMC4792409

[27] Lever J, Krzywinski M, Altman N. Principal component analysis. Nature. 2017. doi: 10.1038/nmeth.4346

[28] Ringnér M. What is principal component analysis?. Nat Biotechnol. 2008;26(3):303–4. doi: 10.1038/nbt0308-303 18327243

[29] Baek S, Ho Y-Y, Ma Y. Using sufficient direction factor model to analyze latent activities associated with breast cancer survival. Biometrics. 2020;76(4):1340–50. doi: 10.1111/biom.13208 31860141 PMC7305041

[30] Carvalho CM, Chang J, Lucas JE, Nevins JR, Wang Q, West M. High-Dimensional Sparse Factor Modeling: Applications in Gene Expression Genomics. J Am Stat Assoc. 2008;103(484):1438–56. doi: 10.1198/016214508000000869 21218139 PMC3017385

[31] Amini A, Masoumi Moghaddam S, Morris DL, Pourgholami MH. The critical role of vascular endothelial growth factor in tumor angiogenesis. Curr Cancer Drug Targets. 2012;12(1):23–43. doi: 10.2174/156800912798888956 22111836

[32] Hall K, Ran S. Regulation of tumor angiogenesis by the local environment. Front Biosci (Landmark Ed). 2010;15(1):195–212. doi: 10.2741/3615 20036815

[33] Xu Y, Peng Y, Shen M, Liu L, Lei J, Gao S, et al. Construction and Validation of Angiogenesis-Related Prognostic Risk Signature to Facilitate Survival Prediction and Biomarker Excavation of Breast Cancer Patients. J Oncol. 2022;2022:1525245. doi: 10.1155/2022/1525245 35498539 PMC9045999

[34] Ahmed T, Begum M. Association between Gene Expression, Clinical Factors and Survival in Patients with Breast Cancer. Journal of Biomedical Analytics. 2018 Feb;1(1):1–14. doi: 10.30577/jba.2018.v1n1.5

[35] Tao D, Wang Y, Zhang X, Wang C, Yang D, Chen J, et al. Identification of Angiogenesis-Related Prognostic Biomarkers Associated With Immune Cell Infiltration in Breast Cancer. Front Cell Dev Biol. 2022;10:853324. doi: 10.3389/fcell.2022.853324 35602610 PMC9121305

[36] Ayoub NM, Sardiah S, Al-Share QY, Alkader MS. Exploring angiogenic pathways in breast cancer: Clinicopathologic correlations and prognostic implications based on gene expression profiles from a large-scale genomic dataset. PLoS One. 2024;19(9):e0310557. doi: 10.1371/journal.pone.0310557 39302921 PMC11414925

[37] Chen S, Cui W, Dong J, Chen W, Dong H, Zhao R. A Novel Signature Based on Angiogenesis-Related Genes Predicts the Prognosis and Immunotherapy Response in HER2-Positive Breast Cancer. J Cancer. 2024;15(14):4731–48. doi: 10.7150/jca.94120 39006091 PMC11242330

[38] Oshi M, Newman S, Tokumaru Y, Yan L, Matsuyama R, Endo I, et al. Intra-Tumoral Angiogenesis Is Associated with Inflammation, Immune Reaction and Metastatic Recurrence in Breast Cancer. Int J Mol Sci. 2020;21(18):6708. doi: 10.3390/ijms21186708 32933189 PMC7555442

[39] The Cancer Imaging Archive. The Cancer Genome Atlas Breast Invasive Carcinoma (TCGA-BRCA) Collection. https://www.cancerimagingarchive.net/collection/tcga-brca/ 2024.

[40] National Cancer Institute Genomic Data Commons. TCGA-BRCA: The Cancer Genome Atlas Breast Invasive Carcinoma Project. https://portal.gdc.cancer.gov/projects/TCGA-BRCA 2026.

[41] Liberzon A. A description of the Molecular Signatures Database (MSigDB) Web site. Methods Mol Biol. 2014;1150:153–60. doi: 10.1007/978-1-4939-0512-6_9 24743996

[42] Wang K, et al. Multi-context principal component analysis. In: 2026. https://arxiv.org/abs/2601.15239

[43] Konishi T. Principal Component Analysis for Experiments. In: 2012. https://arxiv.org/abs/1212.6006

[44] Yu T, Bai Y. Improving gene expression data interpretation by finding latent factors that co-regulate gene modules with clinical factors. BMC Genomics. 2011;12:563. doi: 10.1186/1471-2164-12-563 22087761 PMC3282832

[45] Zhou Y, Wang P, Wang X, Zhu J, Song PX-K. Sparse multivariate factor analysis regression models and its applications to integrative genomics analysis. Genet Epidemiol. 2017;41(1):70–80. doi: 10.1002/gepi.22018 27862229 PMC5154917

[46] Cox DR. Regression Models and Life-Tables. Journal of the Royal Statistical Society Series B: Statistical Methodology. 1972;34(2):187–202. doi: 10.1111/j.2517-6161.1972.tb00899.x

[47] Schoenfeld D. Partial residuals for the proportional hazards regression model. Biometrika. 1982;69(1):239–41. doi: 10.1093/biomet/69.1.239

[48] Grambsch PM, Therneau TM. Proportional hazards tests and diagnostics based on weighted residuals. Biometrika. 1994;81(3):515–26. doi: 10.1093/biomet/81.3.515

[49] Kaplan EL, Meier P. Nonparametric Estimation from Incomplete Observations. Journal of the American Statistical Association. 1958;53(282):457–81. doi: 10.1080/01621459.1958.10501452

[50] Altman DG, Royston P. The cost of dichotomising continuous variables. BMJ. 2006;332(7549):1080. doi: 10.1136/bmj.332.7549.1080 16675816 PMC1458573

[51] Royston P, Altman DG, Sauerbrei W. Dichotomizing continuous predictors in multiple regression: a bad idea. Stat Med. 2006;25(1):127–41. doi: 10.1002/sim.2331 16217841

[52] Brandt J, Garne JP, Tengrup I, Manjer J. Age at diagnosis in relation to survival following breast cancer: a cohort study. World J Surg Oncol. 2015;13:33. doi: 10.1186/s12957-014-0429-x 25889186 PMC4344734

[53] Partridge AH, Hughes ME, Warner ET, Ottesen RA, Wong Y-N, Edge SB, et al. Subtype-Dependent Relationship Between Young Age at Diagnosis and Breast Cancer Survival. J Clin Oncol. 2016;34(27):3308–14. doi: 10.1200/JCO.2015.65.8013 27480155

[54] Kaindal S, Venkataramana B. A comparative analysis of parametric survival models and machine learning methods in breast cancer prognosis. Sci Rep. 2025;15(1):31288. doi: 10.1038/s41598-025-15696-0 40855106 PMC12379255

[55] Alcalá-Corona SA, de Anda-Jáuregui G, Espinal-Enríquez J, Hernández-Lemus E. Network Modularity in Breast Cancer Molecular Subtypes. Front Physiol. 2017;8:915. doi: 10.3389/fphys.2017.00915 29204123 PMC5699328

[56] Vervoort SJ, et al. Global transcriptional analysis identifies a novel role for SOX4 in tumor-induced angiogenesis. eLife. 2018. doi: 10.7554/elife.27706 PMC6277201 30507376

[57] Giatromanolaki A, Sivridis E, Simopoulos C, Polychronidis A, Gatter KC, Harris AL, et al. Differential assessment of angiogenic activity and of vascular survival ability (VSA) in breast cancer. Clin Exp Metastasis. 2002;19(8):673–9. doi: 10.1023/a:1021354028685 12553372

[58] Ramanathan R, Olex AL, Dozmorov M, Bear HD, Fernandez LJ, Takabe K. Angiopoietin pathway gene expression associated with poor breast cancer survival. Breast Cancer Res Treat. 2017;162(1):191–8. doi: 10.1007/s10549-017-4102-2 28062977 PMC5290093

[59] Desmedt C, Haibe-Kains B, Wirapati P, Buyse M, Larsimont D, Bontempi G, et al. Biological processes associated with breast cancer clinical outcome depend on the molecular subtypes. Clin Cancer Res. 2008;14(16):5158–65. doi: 10.1158/1078-0432.CCR-07-4756 18698033

[60] Carey LA, Perou CM, Livasy CA, Dressler LG, Cowan D, Conway K, et al. Race, breast cancer subtypes, and survival in the Carolina Breast Cancer Study. JAMA. 2006;295(21):2492–502. doi: 10.1001/jama.295.21.2492 16757721

[61] Dietze EC, Sistrunk C, Miranda-Carboni G, O’Regan R, Seewaldt VL. Triple-negative breast cancer in African-American women: disparities versus biology. Nat Rev Cancer. 2015;15(4):248–54. doi: 10.1038/nrc3896 25673085 PMC5470637

[62] Newman LA. Disparities in breast cancer and african ancestry: a global perspective. Breast J. 2015;21(2):133–9. doi: 10.1111/tbj.12369 25639288

[63] Forsare C, Bak M, Falck A-K, Grabau D, Killander F, Malmström P, et al. Non-linear transformations of age at diagnosis, tumor size, and number of positive lymph nodes in prediction of clinical outcome in breast cancer. BMC Cancer. 2018;18(1):1226. doi: 10.1186/s12885-018-5123-x 30526533 PMC6286551

[64] Ding P, Tyler J. VanderWeele. Sensitivity Analysis Without Assumptions. Epidemiology. 2016 May;27(3):368–77. doi: 10.1097/ede.0000000000000457 PMC4820664 26841057

[65] Xu J, Tang Z. Progress on angiogenic and antiangiogenic agents in the tumor microenvironment. Front Oncol. 2024;14:1491099. doi: 10.3389/fonc.2024.1491099 39629004 PMC11611712

[66] Choi Y, Jung K. Normalization of the tumor microenvironment by harnessing vascular and immune modulation to achieve enhanced cancer therapy. Exp Mol Med. 2023;55(11):2308–19. doi: 10.1038/s12276-023-01114-w 37907742 PMC10689787

[67] Kuo H-Y, Khan KA, Kerbel RS. Antiangiogenic–immune-checkpoint inhibitor combinations: lessons from phase III clinical trials. Nature Reviews Clinical Oncology. 2024;21(6):468–82. doi: 10.1038/s41571-024-00886-y 38600370

[68] Yang F, Lee G, Fan Y. Navigating tumor angiogenesis: therapeutic perspectives and myeloid cell regulation mechanism. Angiogenesis. 2024;27(3):333–49. doi: 10.1007/s10456-024-09913-z 38580870 PMC11303583

[69] METABRIC - EGA European Genome-Phenome Archive. Ega-archive.org. https://ega-archive.org/studies/EGAS00000000083 Accessed 2026 July 8.

[70] Qiu YL, Zheng H, Devos A, Selby H, Gevaert O. A meta-learning approach for genomic survival analysis. Nat Commun. 2020;11(1):6350. doi: 10.1038/s41467-020-20167-3 33311484 PMC7733508

[71] Peale V, Gerritsen ME. Gene profiling techniques and their application in angiogenesis and vascular development. The Journal of Pathology. 2001;195(1):7–19. doi: 10.1002/path.888 11568887

[72] Hahn CN, et al. Expression profiling reveals functionally important genes and coordinately regulated signaling pathway genes during in vitro angiogenesis. Physiological Genomics. 2005;22(1):57–69. doi: 10.1152/physiolgenomics.00278.2004 15840639

