# Supplementary material for: Multi-dimensional structure and subtype-specific heterogeneity of angiogenesis in breast cancer
Source: PLoS One. 2026 Oct 30;21(10):e0348390. doi: 10.1371/journal.pone.0348390 (PMC13722169; doi:10.1371/journal.pone.0348390)
Supplement: Supplementary file 1 [file pone.0348390.s002.pdf]

PLOS One

Multi-Dimensional and Subtype-Specific Variation in Angiogenesis and Its Association with Survival in Breast Cancer

--Manuscript Draft--

**Manuscript Number:**	PONE-D-26-18657
**Article Type:**	Research Article
**Full Title:**	Multi-Dimensional and Subtype-Specific Variation in Angiogenesis and Its Association with Survival in Breast Cancer
**Short Title:**	Multi-Dimensional Angiogenesis and Its Subtype-Specific Association with Survival in Breast Cancer
**Corresponding Author:**	Dennis Baidoo, M.S<br>University of New Mexico - Albuquerque: The University of New Mexico<br>Accra, Greater Accra GHANA
**Keywords:**	Breast cancer; Gene expression; Angiogenesis; Latent variable model; High-dimensional genomics
**Abstract:**	Background: The prognostic relevance of angiogenesis in breast cancer remains unclear when modeled as a coordinated gene program rather than individual gene effects.<br>Methods: Using RNA sequencing data from 1,227 TCGA-BRCA patients, we summarized 36 angiogenesis-related genes via principal component analysis applied to standardized log-counts per million values. The first three components (Z1–Z3) and a combined score (Z123) were evaluated for association with overall survival using Kaplan–Meier and Cox models adjusted for clinical covariates.<br>Results: The first three components captured a multi-dimensional structure of angiogenesis activity. Only Z2 was significantly associated with survival (HR = 0.91, 95% CI: 0.86–0.96, $p < 0.001$), while Z1, Z3, and Z123 were not. Kaplan–Meier analyses showed no significant survival differences across median-based groups. In adjusted models, the combined angiogenesis score was not independently associated with survival, whereas age remained a strong predictor. Subtype-specific analyses showed a modest effect in Luminal A tumors (HR = 0.63, 95% CI: 0.4034–0.9941, $p < 0.05$) only.<br>Conclusions: Angiogenesis is a multi-dimensional and subtype-dependent process with limited independent prognostic value. Prognostic signal is confined to a specific latent axis rather than overall pathway activity, suggesting that angiogenesis alone is insufficient as a standalone biomarker.
**Order of Authors:**	Dennis Baidoo, M.S
**Additional Information:**	
**Question**	**Response**
**Financial Disclosure**<br><br>Enter a financial disclosure statement that describes the sources of funding for the work included in this submission. Review the submission guidelines for detailed requirements. View published research articles from *PLOS ONE* for specific examples.<br><br>This statement is required for submission and **will appear in the published article** if	The author(s) received no specific funding for this work.

the submission is accepted. Please make sure it is accepted.<br><br>**Funded studies**<br>Enter a statement with the following details:<br>• Initials of the authors who received each award<br>• Grant numbers awarded to each author<br>• The full name of each funder<br>• URL of each funder website<br>• Did the sponsors or funders play any role in the study design, data collection and analysis, decision to publish, or preparation of the manuscript?<br><br>Did you receive funding for this work?	
**Competing Interests**<br><br>Use the instructions below to enter a competing interest statement for this submission. On behalf of all authors, disclose any competing interests that could be perceived to bias this work—acknowledging all financial support and any other relevant financial or non-financial competing interests.<br><br>This statement is required for submission and **will appear in the published article** if the submission is accepted. Please make sure it is accurate and that any funding sources listed in your Funding Information later in the submission form are also declared in your Financial Disclosure statement.<br><br>View published research articles from *PLOS ONE* for specific examples.	The authors have declared that no competing interests exist

**NO authors have competing interests**<br><br>Enter: *The authors have declared that no competing interests exist.*<br><br>**Authors with competing interests**<br><br>Enter competing interest details beginning with this statement:<br><br>*I have read the journal's policy and the authors of this manuscript have the following competing interests: [insert competing interests here]*<br><br>* typeset	
**Ethics Statement**<br><br>Enter an ethics statement for this submission. This statement is required if the study involved:<br><br>• Human participants<br>• Human specimens or tissue<br>• Vertebrate animals or cephalopods<br>• Vertebrate embryos or tissues<br>• Field research<br><br>Write "N/A" if the submission does not require an ethics statement.<br><br>General guidance is provided below. Consult the submission guidelines for detailed instructions. **Make sure that all information entered here is included in the Methods section of the manuscript.**	N/A

**Format for specific study types**<br><br>**Human Subject Research (involving human participants and/or tissue)**<br>• Give the name of the institutional review board or ethics committee that approved the study<br>• Include the approval number and/or a statement indicating approval of this research<br>• Indicate the form of consent obtained (written/oral) or the reason that consent was not obtained (e.g. the data were analyzed anonymously)<br><br>**Animal Research (involving vertebrate animals, embryos or tissues)**<br>• Provide the name of the Institutional Animal Care and Use Committee (IACUC) or other relevant ethics board that reviewed the study protocol, and indicate whether they approved this research or granted a formal waiver of ethical approval<br>• Include an approval number if one was obtained<br>• If the study involved *non-human primates*, add *additional details* about animal welfare and steps taken to ameliorate suffering<br>• If anesthesia, euthanasia, or any kind of animal sacrifice is part of the study, include briefly which substances and/or methods were applied<br><br>**Field Research**<br><br>Include the following details if this study involves the collection of plant, animal, or other materials from a natural setting:<br>• Field permit number<br>• Name of the institution or relevant body that granted permission	
Data Availability<br><br>Before publication, Authors are required to make fully available and without restriction all data underlying their findings. Sharing the data underlying your findings increases the visibility, credibility, and impact of your work, while supporting transparency and future scientific discovery. Please see our PLOS Data Policy page for detailed information on this policy.</p><br>A Data Availability	No data was generated by this study. The following existing data sources were used: The Cancer Genome Atlas (TCGA) Breast Invasive Carcinoma (TCGA-BRCA) dataset, available from the Genomic Data Commons (GDC) Data Portal at https://portal.gdc.cancer.gov/.

Statement, detailing where the data can be accessed, is required at first submission. Insert your Data Availability Statement in the box below.</p> Please see the data reporting of our submission guidelines for instructions on what you need to include in your Data Availability Statement. Examples of acceptable Data Availability Statements are included here for reference:</p> - The minimal data set [and accompanying code, where generated] is available at [repository name] via [DOI hyperlink] - No data was generated by this study. The following existing data sources were used: [dataset name] from [dataset location] available via [web address or DOI hyperlink]. - For Study Protocols: No datasets were generated or analysed during the current study. All relevant data from this study will be made available upon study completion. <i>PLOS allows rare exemptions to address legal and ethical concerns. If you have legal or ethical restrictions, please use the box below to detail these in full sentences for the Journal team to consider.</i></p> This statement will be typeset if the manuscript is accepted for publication.</p>	

Manuscript

Click here to access/download;Manuscript;Baidoo_Angiogenesis_BreastCanc

Multi-Dimensional and Subtype-Specific Variation in Angiogenesis and Its Association with Survival in Breast Cancer

Dennis Baidoo[1,2,3*]

1 Department of Mathematics and Statistics, University of New Mexico, Albuquerque, NM, USA
2 Department of Statistics and Actuarial Science, University of Ghana, Accra, Ghana
3 College of Population Health, University of New Mexico, Albuquerque, NM, USA

¶ Author contributions: The author conceived the study, curated the data, performed the analyses, interpreted the results, and wrote the manuscript.

* dbaidoo@unm.edu

Abstract

Background: The prognostic relevance of angiogenesis in breast cancer remains unclear when modeled as a coordinated gene program rather than individual gene effects. **Methods:** Using RNA sequencing data from 1,227 TCGA-BRCA patients, we summarized 36 angiogenesis-related genes via principal component analysis (PCA) applied to standardized log-counts per million values. The first three components (Z_1–Z_3) and a combined score (Z_{123}) were evaluated for association with overall survival using Kaplan–Meier and Cox models adjusted for clinical covariates. **Results:** The first three components captured a multi-dimensional structure of angiogenesis activity. Only Z_2 was significantly associated with survival (HR $= 0.91$, 95% CI: 0.86–0.96, $p < 0.001$), while Z_1, Z_3, and Z_{123} were not. Kaplan–Meier analyses showed no significant survival differences across median-based groups. In adjusted models, the combined angiogenesis score was not independently associated with survival, whereas age remained a strong predictor. Subtype-specific analyses showed a modest effect in Luminal A tumors (HR=0.63, 95% CI: 0.4034–0.9941, $p < 0.05$) only. **Conclusions:** Angiogenesis is a multi-dimensional and subtype-dependent process with limited independent prognostic value. Prognostic signal is confined to a specific latent axis rather than overall pathway activity, suggesting angiogenesis alone is insufficient as a standalone biomarker.

Keywords: Breast cancer , Gene expression , Angiogenesis , Latent variable model , High-dimensional genomics

Author summary

Angiogenesis, the process by which tumors form new blood vessels, is widely believed to play an important role in cancer progression and patient

survival. However, most studies examine individual genes rather than treating angiogenesis as a coordinated biological process. In this study, we used gene expression data from breast cancer patients to model angiogenesis as a multi-dimensional gene program. By summarizing groups of related genes into latent components, we assessed whether angiogenesis activity is associated with survival outcomes. We found that while angiogenesis exhibits a complex internal structure, it does not provide a strong or consistent signal for predicting survival overall. Instead, only one component showed a modest association, and this effect varied across tumor subtypes, appearing primarily in Luminal A but not in more aggressive subtypes such as Basal tumors. These findings suggest that angiogenesis alone may not be sufficient as a standalone prognostic biomarker and should be interpreted within the broader context of tumor biology.

Introduction

Do coordinated biological pathways provide meaningful and consistent prognostic information for cancer survival? There have been critical improvements and advances in high-throughput sequencing technologies for breast cancer research over two decades, enhancing a very comprehensive profiling of tumor gene expression at an unprecedented scale [1–6]. Large-scale initiatives such as The Cancer Genome Atlas (TCGA) have provided thousands of tumor samples with linked molecular and clinical data, providing new avenues and opportunities to comprehend the biological mechanisms governing disease progression and survival [7–12].

However, in the abundance of genomic data, a significant amount of the existing literature has focused and contributed primary on identifying individual genes or constructing prognostic signatures associated with survival outcomes [13–16]. While some of these studies have produced valuable genomic insights, they often treat genes independently or solely rely on two-stage analytical techniques that may not fully account for biological coordination processes or associated measurement error. Prior studies have established that cancer progression is rarely driven by isolated genes but rather, the reflection of the activity of a complex biological program, specifically, groups of genes concerned with the regulation of such processes, including angiogenesis, immune evasion, and metastasis [12, 17–20].

Angiogenesis, the formation of new blood vessels, plays a central role in tumor growth and dissemination [21–23]. These processes support the promotion of the circulation of oxygen and nutrient delivery to malignant tissue, where angiogenic signaling significantly contributes to aggressiveness and poorer clinical outcomes. Although prior studies have examined associations between angiogenesis-related genes and survival in breast cancer, fewer have modeled angiogenesis as a coherent latent biological program while explicitly accounting for clinical confounding and measurement variability in gene expression data [21, 24–27].

Figure 1: **Conceptual framework for modeling angiogenesis as a multi-dimensional latent process**. Gene expression is summarized into latent components that capture coordinated biological activity and relate to subtype and survival.

This study investigates the relationship between angiogenesis gene pro-

gram activity and overall survival in breast cancer using a latent variable framework. Using RNA sequencing data and clinical annotations from the TCGA-BRCA cohort, we characterize angiogenesis as a coordinated, multi-gene process rather than a single pathway signal and examine its association with survival outcomes. By integrating biological structure with statistical modeling, this work aims to clarify the extent to which angiogenesis contributes to patient prognosis and whether its effects are consistent across tumor subtypes.

Methods and materials

Data Source and Preprocessing

Breast cancer data were obtained from The Cancer Genome Atlas - Breast Invasive Carcinoma (TCGA-BRCA) cohort. RNA sequencing (RNA-seq) gene expression profiles and corresponding clinical annotations were downloaded using the `TCGAbiolinks` package in R. The dataset included 1,227 primary breast tumor samples with available overall survival information. For each patient, gene expression was quantified using STAR-aligned RNA sequencing data, resulting in gene-level count measurements across 60,660 genes. These data were subsequently normalized and standardized for downstream analysis, overall survival time (days to death or last follow-up), vital status (deceased or censored), clinical covariates including age at diagnosis, tumor stage, and molecular subtype [11]. Survival time was defined as the number of days from diagnosis to death or last follow-up.

The Hallmark Angiogenesis gene set was obtained from the Molecular Signatures Database (MSigDB) [28, 29]. Thirty-six genes were matched in the TCGA-BRCA dataset and retained for analysis.

Exploratory Latent Structure Assessment

To characterize the latent structure of angiogenesis activity, principal component analysis (PCA) was applied to the standardized gene expression matrix. PCA was used to identify orthogonal components representing dominant patterns of variation in angiogenesis-related gene expression. The first three principal components were retained for downstream analysis based on their contribution to variance and interpretability. A combined angiogenesis score was constructed as the sum of the retained components to capture overall angiogenesis activity across multiple latent dimensions.

In assessing the intrinsic dimensionality of the angiogenesis gene program activity, PCA was performed on standardized log-counts per million (log-CPM) expression values. The first three latent components explained 22.6%, 9.1%, and 7.7% of the variance, respectively, highlighting a multi-dimensional structure without a single dominant axis, with the remaining variation more likely due to random noise based on the proportion of variance explained and inspection of the scree plot. Cox proportional hazards regression applied to the first three component scores showed that the second principal component was significantly associated with overall survival ($p < 0.05$), whereas the first and third were not, motivating the use of a multi-factor latent modeling framework (Table 2).

Gene Module Definition

To study coordinated biological activity, we focused on a predefined angiogenesis-related gene set obtained from the Molecular Signatures Database (MSigDB). The selected module consisted of p genes associated with vascular development and angiogenic signaling pathways. Only genes with sufficient expression variability across samples were retained.

Data Structure and Notation

Let $i = 1, \ldots, n$ index breast cancer patients from the TCGA-BRCA cohort. For each patient we observe the following, $G_i = (G_{i1}, \ldots, G_{ip})$ - standardized log-CPM expression measurements for genes in the Hallmark Angiogenesis set ($p = 36$), $X_i \in \mathbb{R}^{nxp}$: clinical covariates (e.g., age, tumor stage, molecular subtype), T_i: true event time (overall survival), and C_i: censoring time.

Multi-Factor Latent Gene Module Model

Since our PCA analysis indicated a multi-dimensional structure of the angiogenesis gene program, we let

$$\mathbf{Z}_i = (Z_{i1}, Z_{i2}, Z_{i3})^\top$$

denote the patient-specific scores corresponding to the first three principal components of the standardized gene expression matrix.

Let $\mathbf{G}_i = (G_{i1}, \ldots, G_{ip})^\top$ denote the standardized expression levels for the p angiogenesis-related genes for patient i. PCA decomposes the gene expression matrix as

$$G_i = \Lambda Z_i + \epsilon_i,$$

where $Z_i \in \mathbb{R}^k$ is the vector of latent components (principal component scores), $\Lambda \in \mathbb{R}^{p \times k}$ is the loading matrix, and $\epsilon_i \in \mathbb{R}^p$ is the residual term representing variation not captured by the retained components with mean zero (0) and standard deviation σ. Where ϵ_i captures the remaining unexplained variation. The principal components were obtained by eigen-decomposition of the sample covariance matrix of the standardized gene expression data.

These components provide a low-dimensional representation of coordinated angiogenesis-related transcriptional activity, capturing dominant patterns of variation across patients, with Z_1, Z_2, and Z_3 as the first three principal components, and a combined angiogenesis score (Z_{123}) was defined as the sum of the first three component scores to represent aggregate pathway activity.

Survival Analysis

The association between latent angiogenesis activity and overall survival was evaluated using Cox proportional hazards models. Let T_i denote the survival time and δ_i the event indicator for patient i. The hazard function was specified as

$$\lambda(t \mid \mathbf{Z}_i, X_i) = \lambda_0(t) \exp\left(\boldsymbol{\beta}^\top \mathbf{Z}_i + \boldsymbol{\gamma}^\top X_i\right),$$

where $\lambda_0(t)$ is the baseline hazard function. Hazard ratios (HRs) were reported as $\exp(\beta_k)$ for each latent component. Both univariable and

multivariable models were fitted, with the multivariable model adjusting for clinical covariates. No causal interpretation is assumed; the estimated associations reflect conditional relationships between angiogenesis activity and survival under the proportional hazards assumption.

Results

Baseline Characteristics

The baseline characteristics stratified by angiogenesis activity are presented in Table 1. The low and high angiogenesis groups were comparable with respect to age and follow-up time, with no statistically significant differences observed (age: $p > 0.05$; time: $p > 0.05$). Similarly, survival status did not differ significantly between groups ($p > 0.05$), indicating no strong unadjusted association between angiogenesis and overall survival.

In contrast, tumor subtype distribution differed significantly between groups ($p < 0.001$). The high angiogenesis group was enriched for Luminal A and Luminal B subtypes, whereas the low angiogenesis group contained a higher proportion of Basal tumors. These findings indicate that angiogenesis activity is not uniformly distributed across molecular subtypes, suggesting underlying biological heterogeneity that may influence its relationship with survival outcomes. These subtype-specific differences motivated further investigation of the latent structure of angiogenesis activity and its association with survival outcomes.

Table 1: Baseline characteristics by angiogenesis group

Characteristic	Low (N = 518)[1]	High (N = 509)[1]	p-value[2]
Age	0.03 (1.00)	-0.01 (0.97)	0.5
Time (in years)	3.50 (3.57)	3.42 (2.98)	0.4
Status	69 (13%)	75 (15%)	0.5
Subtype			<0.001
Basal	126 (24%)	62 (12%)	
Her2	43 (8.3%)	29 (5.7%)	
LumA	239 (46%)	292 (57%)	
LumB	88 (17%)	111 (22%)	
Normal	22 (4.2%)	15 (2.9%)	

[1] Mean (SD); n (%)
[2] Wilcoxon rank sum test; Pearson's Chi-squared test

Association Between Angiogenesis Components and Survival

The association between individual angiogenesis principal components and overall survival is presented in Table 2. Among the three components, only the second principal component (Z_2) was significantly associated with survival (HR = 0.91, 95% CI: 0.86–0.96, $p < 0.001$), indicating a protective effect. In contrast, Z_1(HR = 0.97, 95% CI: 0.918–1.020, $p > 0.05$) and Z_3 (HR = 1.02, 95% CI: 0.936–1.103, $p > 0.05$) were not significantly associated with survival.

Despite the statistical significance of Z_2, the overall model discrimination was modest (concordance index = 0.545), suggesting limited predictive

performance. These findings indicate that survival associations are not uniformly distributed across angiogenesis dimensions, but are instead concentrated within a specific latent component, supporting a multi-dimensional structure of angiogenesis with heterogeneous prognostic relevance. To further evaluate the overall effect of angiogenesis activity and interpret the biological meaning of the survival-associated component (Z_2), we next examined survival differences using combined latent scores and nonparametric survival analysis, in addition to its gene loadings. Z_2 was characterized by positive contributions from genes such as *ITGAV*, *OLR1*, *VCAN*, and *PTK2*, which are involved in cell adhesion, extracellular matrix remodeling, and angiogenic signaling. In contrast, negative loadings were observed for genes including *TIMP1*, *JAG2*, and *PDGFA*, which are associated with matrix regulation and signaling pathways involved in vascular stability and remodeling.

This opposing pattern of gene contributions suggests that Z_2 captures a balance between pro-angiogenic extracellular matrix activity and regulatory or stabilizing pathways. The observed association of Z_2 with survival, therefore, reflects a specific biological axis within the broader angiogenesis program rather than a global effect of angiogenesis activity, highlighting that the prognostic relevance of angiogenesis arises from specific transcriptional programs rather than overall pathway activity.

Figure 2: **Latent transcriptional organization of the angiogenesis gene program in TCGA-BRCA.** (A) Gene–gene Pearson correlation matrix of the 36 angiogenesis genes across 1,227 breast cancer patients. Hierarchical clustering reveals structured positive correlation blocks, indicating coordinated co-expression submodules rather than a single homogeneous program. (B) Heatmap of log-transformed and Z-score–standardized gene expression values across patients. Distinct patient subgroups exhibit coordinated upregulation (red) and downregulation (blue) of angiogenesis genes, supporting a multi-dimensional latent expression architecture.

Latent Structure of Angiogenesis Gene Expression

The transcriptional organization of angiogenesis-related genes is shown in Figure 2. The gene–gene correlation matrix (Figure 2A) revealed structured blocks of positive correlations, indicating the presence of coordinated co-expression modules rather than a single homogeneous gene program. Hierarchical clustering further supported the existence of distinct gene clusters with shared expression patterns.

Consistent with this modular structure, the gene expression heatmap (Figure 2B) demonstrated substantial heterogeneity across patients, with distinct groups exhibiting coordinated upregulation and downregulation of angiogenesis genes. These patterns suggest that angiogenesis activity is organized along multiple latent dimensions rather than a single dominant axis. The findings provide evidence for a multi-dimensional transcriptional architecture underlying angiogenesis, motivating the use of principal component analysis to capture its latent structure and investigate its association with survival outcomes.

Table 2: Association between angiogenesis principal components and overall survival in TCGA-BRCA patients (Cox proportional hazards model).

	Hazard Ratio	95% CI	z	p value
Z_1	0.968	0.918–1.020	-1.23	0.219
Z_2	0.908	0.861–0.958	-3.53	< 0.001
Z_3	1.016	0.936–1.103	0.39	0.698

$n = 1{,}227$ patients; 201 events.

Concordance index = 0.545 (SE = 0.025).

Likelihood ratio test $p < 0.01$.

Figure 3: **Latent angiogenesis components capture molecular subtype structure and survival-associated heterogeneity.** (A) Distribution of latent components (Z_1-Z_3) across PAM50 breast cancer subtypes (Basal, Her2, LumA, LumB, Normal). The violin plots reveal subtype-specific shifts in latent expression patterns, indicating that angiogenesis-related transcriptional programs are not homogeneous but vary systematically across molecular subtypes. (B) Distribution of latent components stratified by survival status (Alive vs Dead). Subtle shifts in component distributions suggest that angiogenesis activity contributes modestly to survival differences, consistent with a diffuse but biologically meaningful signal rather than a single dominant prognostic axis.

Latent Components Capture Subtype Structure and Survival Heterogeneity

The distributions of latent angiogenesis components across molecular subtypes and survival status are shown in Figure 3. Clear subtype-specific patterns were observed across all three components (Z_1-Z_3) (Figure 3A). In particular, Basal, Luminal, and Normal subtypes exhibited distinct shifts in component distributions, indicating that angiogenesis-related transcriptional programs vary systematically across molecular subtypes rather than forming a homogeneous signal.

When stratified by survival status (Figure 3B), differences in component distributions between alive and deceased patients were modest. While slight shifts were observed, particularly in Z_1 and Z_3, the overlap between groups remained substantial, suggesting that angiogenesis activity contributes only weakly to survival discrimination at the individual component level.

Together, these findings demonstrate that latent angiogenesis components primarily capture subtype-specific biological variation, with only a diffuse and modest association with survival outcomes. This supports the view that angiogenesis operates as a heterogeneous, multi-dimensional process with limited standalone prognostic power. To further quantify the relationship between angiogenesis activity and survival, we next evaluated survival differences using Kaplan–Meier analysis and Cox proportional hazards models.

Unadjusted and Adjusted Survival Analysis of Latent Angiogenesis Components

Unadjusted Kaplan–Meier survival curves for the latent angiogenesis components are shown in Figure 4. No statistically significant differences in

overall survival were observed between high and low groups for any of the individual components (Z_1: $p > 0.05$, Z_2: $p > 0.05$, Z_3: $p > 0.05$).

Similarly, the combined angiogenesis score (Z_{123}) did not demonstrate a statistically significant association with survival ($p > 0.05$), although a modest trend toward poorer survival was observed in the high angiogenesis group.

The substantial overlap between survival curves across all components indicates that angiogenesis activity alone does not strongly discriminate survival outcomes in an unadjusted setting. These findings are consistent with the multi-dimensional structure observed in earlier analyses, suggesting that the prognostic signal of angiogenesis is diffuse and not driven by a single dominant axis. To further evaluate the association between angiogenesis activity and survival while accounting for clinical covariates, we next examined Cox proportional hazards models.

Kaplan–Meier survival analyses based on median-split grouping of the latent components while adjusting for clinical annotations are shown in Figure 5. Consistent with the unadjusted analysis, none of the individual components demonstrated statistically significant differences in survival (Z_1: $p > 0.05$, Z_2: $p > 0.05$, Z_3: $p > 0.05$).

The combined angiogenesis score (Z_{123}) exhibited a modest trend toward poorer survival in the high group ($p > 0.05$), although this did not reach statistical significance. Compared to the unadjusted analyses, the separation between groups is slightly more pronounced for Z_{123}, suggesting a weak but consistent signal when aggregating across latent dimensions.

Therefore, the substantial overlap between survival curves indicates that angiogenesis does not provide strong discrimination of survival outcomes. These findings further support the interpretation that angiogenesis-related prognostic information is distributed across multiple latent components rather than concentrated in a single dominant axis.

Figure 4: **Unadjusted Kaplan–Meier survival curves for latent angiogenesis components.** No significant survival differences were observed for Z_1-Z_3 or the combined score (Z_{123}), although a modest trend toward poorer survival is seen in the high Z_{123} group.

Figure 5: **Kaplan–Meier survival analysis of latent angiogenesis components.** Survival curves comparing high versus low groups (median split) for the first three latent components (Z_1-Z_3) and their combined score (Z_{123}). None of the individual components showed statistically significant survival differences (Z_1: $p = 0.29$, Z_2: $p = 0.48$, Z_3: $p = 0.39$). The combined angiogenesis score (Z_{123}) demonstrated a trend toward poorer survival in the high group ($p = 0.10$), suggesting that angiogenic activity is distributed across multiple latent dimensions rather than driven by a single dominant component. Shaded regions represent 95% confidence intervals.

Adjusted hazard ratios from the multivariable Cox proportional hazards model are presented in Figure 6. After adjusting for age and molecular subtype, the combined angiogenesis score (Z_{123}) was not significantly associated with overall survival, with an estimated hazard ratio close to unity.

In contrast, age remained a strong and statistically significant predictor of survival, consistent with established clinical evidence [30–32]. Molecular

subtype also demonstrated variability in hazard, although with wider confidence intervals reflecting heterogeneity across groups. Overall, the adjusted analysis confirms that angiogenesis does not independently predict survival when accounting for key clinical covariates. These findings are consistent with earlier results, indicating that the prognostic signal of angiogenesis is modest and largely driven by subtype-specific biological variation rather than a strong independent effect.

Figure 6: **Adjusted hazard ratios for clinical covariates and angiogenesis gene program activity.** Hazard ratios (HR) and 95% confidence intervals are shown for age at diagnosis and molecular subtypes in a multivariable Cox proportional hazards model. Points represent estimated hazard ratios and horizontal lines indicate 95% confidence intervals; the dashed vertical line at HR = 1 denotes no effect.

Discussion

In this study, we examined the prognostic role of angiogenesis in breast cancer using a latent variable framework derived from gene expression data. Our results show that angiogenesis is not a single homogeneous pathway but a multi-dimensional biological process with subtype-specific variation, leading to a modest and diffuse association with survival (Figure 3). Although one latent component (Z_2) was significantly associated with survival, the overall angiogenesis signal did not strongly discriminate outcomes, as evidenced by non-significant Kaplan–Meier analyses and a near-null combined effect in (Z_{123}; HR = 1.001, 95% CI: 0.9996–1.0033, $p > 0.05$).

The observed weak global effect can be explained by the underlying transcriptional structure of angiogenesis [33, 34]. Gene correlation and clustering analyses revealed coordinated but distinct expression modules (Figure 2), and principal component analysis demonstrated that prognostic information is concentrated within a specific latent axis rather than distributed uniformly across all components (Table 2). In particular, Z_2 captured a balance between extracellular matrix remodeling and regulatory signaling pathways, suggesting that survival associations arise from specific biological contrasts rather than overall angiogenesis activity [35–37]. This multi-dimensional organization dilutes the impact of any single component when aggregated, resulting in a diffuse overall signal.

These findings were robust across multiple sensitivity analyses. Adjustment for clinical covariates confirmed that angiogenesis is not an independent predictor of survival in Figure 6, and model comparison indicated that inclusion of Z_{123} did not significantly improve model fit beyond age and subtype ($p > 0.05$). After adjusting for age and molecular subtype, the angiogenesis score remained non-significant, and inclusion of the combined angiogenic component did not significantly improve model fit compared to clinical covariates alone (likelihood ratio test $p > 0.05$). In contrast, age remained a strong predictor of survival (HR=1.58, 95% CI: 1.324–1.880, $p < 0.05$). Additional analyses showed no evidence of nonlinearity or time-dependent effects [38], suggesting that the association is stable across modeling assumptions. Furthermore, the estimated E-value was small (E-value = 1.04), indicating that even minimal unmeasured confounding could explain the observed effect, reinforcing its limited magnitude [39].

Importantly, subtype-stratified analyses revealed that the effect of angiogenesis is context-dependent, with a modest association observed in Luminal A tumors (HR=0.63, 95% CI: 0.4034-0.9941, $p < 0.05$) but not in Basal tumors (Table 1 and Figure 3). This heterogeneity suggests that angiogenesis primarily reflects subtype-specific biology rather than a universal prognostic mechanism. These findings provide a potential explanation for inconsistent results in prior studies that have treated angiogenesis as a single pathway [13–16]. Overall, our results highlight the importance of modeling biological processes as multi-dimensional systems and suggest that future prognostic models should integrate angiogenesis with other tumor microenvironmental features to improve predictive performance.

Despite these findings, several limitations should be considered. First, the analysis is based on observational data from the TCGA-BRCA cohort, which lacks standardized treatment assignment and comprehensive clinical annotations. As a result, treatment-related confounding could not be fully accounted for, and the observed associations should be interpreted as prognostic rather than causal. Additionally, the cohort represents a heterogeneous population collected across multiple centers, which may introduce variability in measurement and clinical management. While we adjusted for key covariates such as age and subtype, residual confounding cannot be excluded.

Second, the latent variable framework relies on principal component analysis, which captures linear combinations of gene expression and may not fully represent complex, nonlinear, or context-specific biological interactions. Although sensitivity analyses showed no evidence of strong nonlinearity or time-dependent effects, subtle interactions between angiogenesis and other tumor microenvironmental processes may not be captured in the current model. Furthermore, the use of a predefined angiogenesis gene set may omit relevant pathways or genes that contribute to vascular dynamics, potentially limiting the scope of the analysis [40, 41].

Conclusion

Throughout this study, we demonstrated that angiogenesis in breast cancer exhibits a multi-dimensional and subtype-dependent structure, resulting in a modest and diffuse association with survival. While some specific latent components showed a significant association with survival, the overall angiogenesis signal did not strongly discriminate outcomes, as reflected by overlapping Kaplan–Meier curves and a near-null effect for the combined score.

Multivariable analyses further confirmed the limited independent prognostic value of angiogenesis, and age improved overall survival, establishing that clinical factors dominate prognostic performance. Moreover, Sensitivity analyses consistently supported these findings. The estimated E-value for the angiogenesis effect was small, indicating that minimal unmeasured confounding could explain the observed association.

Modeling angiogenesis as a multi-dimensional and subtype-dependent process provides a more accurate framework for understanding its role in breast cancer survival. In this study, we showed that angiogenesis exhibits a modest and diffuse association with survival, with no strong independent prognostic effect after adjustment for clinical covariates. These findings suggest that angiogenesis is not a universal biomarker but reflects

context-specific biological processes that vary across tumor subtypes. Incorporating such multi-dimensional representations into prognostic modeling may improve the interpretation of pathway-level effects and support more biologically informed approaches to risk stratification.

Supporting information

There is no supporting information for this manuscript.

Funding information

The author received no specific funding for this work.

Acknowledgments

I would like to thank the contributors of The Cancer Genome Atlas (TCGA) project for generating and making publicly available the data used in this study. I also acknowledge the developers and maintainers of the open-source R packages and computational tools that supported the analysis. I am grateful to colleagues and mentors for their valuable feedback and discussions that helped improve this work.

References

1. Stover DG and Wagle N. Precision Medicine in Breast Cancer: Genes, Genomes, and the Future of Genomically Driven Treatments. Current Oncology Reports 2015 Feb; 17. DOI: 10.1007/s11912-015-0438-0
2. Karn T. High-Throughput Gene Expression and Mutation Profiling: Current Methods and Future Perspectives. Breast Care 2013; 8:401–6. DOI: 10.1159/000357461
3. Curtis C. Genomic profiling of breast cancers. Current Opinion in Obstetrics and Gynecology 2015 Feb; 27:34–9. DOI: 10.1097/gco.0000000000000145
4. Orsini A, Diquigiovanni C, and Bonora E. Omics Technologies Improving Breast Cancer Research and Diagnostics. International Journal of Molecular Sciences 2023 Jan; 24:12690. DOI: 10.3390/ijms241612690
5. Reis-Filho JS. Next-generation sequencing. Breast Cancer Research 2009 Dec; 11. DOI: 10.1186/bcr2431
6. Amevor R, Kubuafor E, Baidoo D, Salifu J, Gonnage KM, and Okeke OJ. Integrative Prognostic Modeling of Breast Cancer Survival with Gene Expression and Clinical Data. arXiv.org, 2025. Available from: https://arxiv.org/abs/2508.16740
7. Lee JS. Exploring cancer genomic data from the cancer genome atlas project. BMB Reports 2016 Nov; 49:607–11. DOI: 10.5483/bmbrep.2016.49.11.145
8. Chang JTH, Lee YM, and Huang RS. The impact of the Cancer Genome Atlas on lung cancer. Translational Research 2015 Dec; 166:568–85. DOI: 10.1016/j.trsl.2015.08.001

9. Colaprico A et al. TCGAbiolinks: an R/Bioconductor package for integrative analysis of TCGA data. Nucleic Acids Research 2015 Dec; 44:e71–e71. DOI: 10.1093/nar/gkv1507
10. Liu J, Lichtenberg T, Hoadley KA, Poisson LM, Lazar AJ, Cherniack AD, et al. An Integrated TCGA Pan-Cancer Clinical Data Resource to Drive High-Quality Survival Outcome Analytics. Cell 2018 Apr; 173:400–416.e11. DOI: 10.1016/j.cell.2018.02.052
11. Tomczak K, Czerwińska P, and Wiznerowicz M. Review the Cancer Genome Atlas (TCGA): an Immeasurable Source of Knowledge. Współczesna Onkologia 2015; 1A:68–77. DOI: 10.5114/wo.2014.47136. Available from: https://www.ncbi.nlm.nih.gov/pmc/articles/PMC4322527/
12. Wang Z, Jensen MA, and Zenklusen JC. A Practical Guide to The Cancer Genome Atlas (TCGA). Methods in Molecular Biology 2016 :111–41. DOI: 10.1007/978-1-4939-3578-9_6
13. West M. Embracing the complexity of genomic data for personalized medicine. Genome Research 2006 May; 16:559–66. DOI: 10.1101/gr.3851306
14. Qiu YL, Zheng H, Devos A, Selby H, and Gevaert O. A meta-learning approach for genomic survival analysis. Nature Communications 2020 Dec; 11. DOI: 10.1038/s41467-020-20167-3
15. Anaya J, Reon BJ, Chen W, Bekiranov S, and Dutta A. A pan-cancer analysis of prognostic genes. 2016 Feb; 3:e1499–e1499. DOI: 10.7717/peerj.1499
16. Zhao Q, Shi X, Xie Y, Huang J, Shia B, and Ma S. Combining multidimensional genomic measurements for predicting cancer prognosis: observations from TCGA. Briefings in Bioinformatics 2014 Mar; 16:291–303. DOI: 10.1093/bib/bbu003. Available from: https://academic.oup.com/bib/article/16/2/291/246070
17. Creixell P et al. Pathway and network analysis of cancer genomes. Nature Methods 2015 Jun; 12:615–21. DOI: 10.1038/nmeth.3440
18. Edelman EJ, Guinney J, Chi JT, Febbo PG, and Mukherjee S. Modeling Cancer Progression via Pathway Dependencies. PLoS Computational Biology 2008 Feb; 4. Ed. by Tucker-Kellogg G:e28. DOI: 10.1371/journal.pcbi.0040028
19. Xu M, Kao MCJ, Nunez-Iglesias J, Nevins JR, West M, and Zhou XJ. An integrative approach to characterize disease-specific pathways and their coordination: a case study in cancer. BMC Genomics 2008 Mar; 9. DOI: 10.1186/1471-2164-9-s1-s12
20. Chen B, Aouiche C, and Shang X. Integrating Multiple Datasets to Discover Stage-Specific Cancer Related Genes and Stage-Specific Pathways. Lecture Notes in Computer Science 2019 :240–50. DOI: 10.1007/978-3-030-17938-0_22
21. Carvalho CM, Chang J, Lucas JE, Nevins JR, Wang Q, and West M. High-Dimensional Sparse Factor Modeling: Applications in Gene Expression Genomics. Journal of the American Statistical Association 2008 Dec; 103:1438–56. DOI: 10.1198/016214508000000869

22. Amini A, Masoumi Moghaddam S, L. Morris D, and H. Pourgholami M. The Critical Role of Vascular Endothelial Growth Factor in Tumor Angiogenesis. Current Cancer Drug Targets 2012 Jan; 12:23–43. DOI: 10.2174/156800912798888956
23. Hall K. Regulation of tumor angiogenesis by the local environment. Frontiers in Bioscience 2010; 15:195. DOI: 10.2741/3615
24. Baek S, Ho YY, and Ma Y. Using sufficient direction factor model to analyze latent activities associated with breast cancer survival. Biometrics 2020 Jan; 76:1340–50. DOI: 10.1111/biom.13208
25. Xu Y et al. Construction and Validation of Angiogenesis-Related Prognostic Risk Signature to Facilitate Survival Prediction and Biomarker Excavation of Breast Cancer Patients. Journal of Oncology 2022 Apr; 2022. Ed. by Raza F:1–21. DOI: 10.1155/2022/1525245
26. Ahmed T and Begum M. Association between Gene Expression, Clinical Factors and Survival in Patients with Breast Cancer. Journal of Biomedical Analytics 2018 Feb; 1:1–14. DOI: 10.30577/jba.2018.v1n1.5
27. Merl D, Chen JLY, Chi JT, and West M. An integrative analysis of cancer gene expression studies using Bayesian latent factor modeling. The Annals of Applied Statistics 2009 Dec; 3. DOI: 10.1214/09-aoas261
28. Liberzon A. A Description of the Molecular Signatures Database (MSigDB) Web Site. Methods in molecular biology 2014 Jan :153–60. DOI: 10.1007/978-1-4939-0512-6_9
29. Liberzon A, Birger C, Thorvaldsdóttir H, Ghandi M, Mesirov JP, and Tamayo P. The Molecular Signatures Database Hallmark Gene Set Collection. Cell Systems 2015 Dec; 1:417–25. DOI: 10.1016/j.cels.2015.12.004
30. Brandt J, Garne J, Tengrup I, and Manjer J. Age at diagnosis in relation to survival following breast cancer: a cohort study. World Journal of Surgical Oncology 2015; 13:33. DOI: 10.1186/s12957-014-0429-x
31. Partridge AH et al. Subtype-Dependent Relationship Between Young Age at Diagnosis and Breast Cancer Survival. Journal of Clinical Oncology 2016 Sep; 34:3308–14. DOI: 10.1200/jco.2015.65.8013. Available from: https://ascopubs.org/doi/full/10.1200/JCO.2015.65.8013
32. Kaindal S and Venkataramana B. A comparative analysis of parametric survival models and machine learning methods in breast cancer prognosis. Scientific Reports 2025 Aug; 15. DOI: 10.1038/s41598-025-15696-0
33. Alcalá-Corona SA, Anda-Jáuregui G de, Espinal-Enriquez J, Tovar H, and Hernández-Lemus E. Network Modularity and Hierarchical Structure in Breast Cancer Molecular Subtypes. Springer Proceedings in Complexity 2018 :352–8. DOI: 10.1007/978-3-319-96661-8_36
34. Vervoort SJ et al. Global transcriptional analysis identifies a novel role for SOX4 in tumor-induced angiogenesis. eLife 2018 Dec; 7. DOI: 10.7554/elife.27706

35. Giatromanolaki A et al. Differential assessment of angiogenic activity and of vascular survival ability (VSA) in breast cancer. Clinical & Experimental Metastasis 2002 Dec; 19:673–9. DOI: 10.1023/a:1021354028685

36. Ramanathan R, Olex AL, Dozmorov M, Bear HD, Fernandez LJ, and Takabe K. Angiopoietin pathway gene expression associated with poor breast cancer survival. Breast Cancer Research and Treatment 2017 Jan; 162:191–8. DOI: 10.1007/s10549-017-4102-2

37. Desmedt C et al. Biological Processes Associated with Breast Cancer Clinical Outcome Depend on the Molecular Subtypes. Clinical Cancer Research 2008 Aug; 14:5158–65. DOI: 10.1158/1078-0432.ccr-07-4756

38. Forsare C et al. Non-linear transformations of age at diagnosis, tumor size, and number of positive lymph nodes in prediction of clinical outcome in breast cancer. BMC Cancer 2018 Dec; 18. DOI: 10.1186/s12885-018-5123-x

39. Ding P and VanderWeele TJ. Sensitivity Analysis Without Assumptions. Epidemiology 2016 May; 27:368–77. DOI: 10.1097/ede.0000000000000457

40. Peale FV and Gerritsen ME. Gene profiling techniques and their application in angiogenesis and vascular development. The Journal of Pathology 2001; 195:7–19. DOI: 10.1002/path.888

41. Hahn CN et al. Expression profiling reveals functionally important genes and coordinately regulated signaling pathway genes during in vitro angiogenesis. Physiological Genomics 2005 Jun; 22:57–69. DOI: 10.1152/physiolgenomics.00278.2004

All figures for the manuscript
Click here to access/download;Figure;Baidoo_Angiogenesis_BreastCancer_Fi

Multi-Dimensional and Subtype-Specific Variation in Angiogenesis and Its Association with Survival in Breast Cancer

Dennis Baidoo[1,2*]

1 Department of Mathematics and Statistics, University of New Mexico, Albuquerque, NM, USA
2 Department of Statistics and Actuarial Science, University of Ghana, Accra, Ghana
3 College of Population Health, University of New Mexico, Albuquerque, NM, USA

¶ Author contributions: The author conceived the study, curated the data, performed the analyses, interpreted the results, and wrote the manuscript.

* dbaidoo@unm.edu

Figures

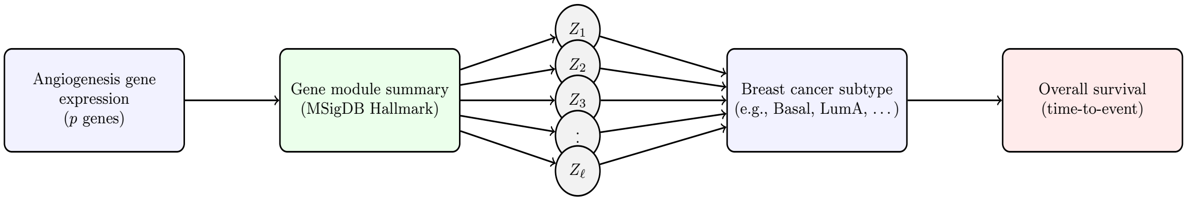

Fig 1. Conceptual framework for modeling angiogenesis as a multi-dimensional latent process. Gene expression is summarized into latent components that capture coordinated biological activity and relate to subtype and survival.

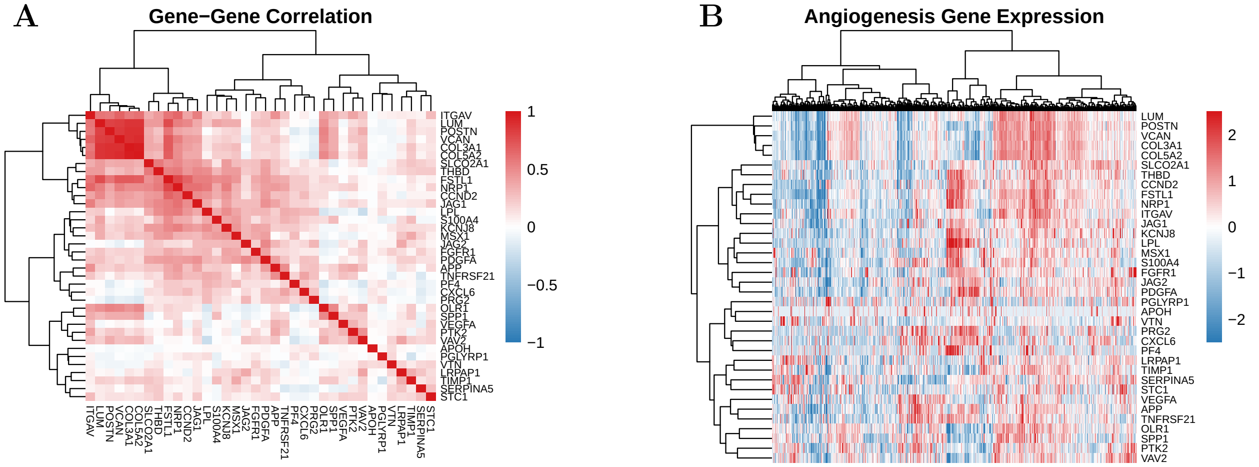

Fig 2. Latent transcriptional organization of the angiogenesis gene program in TCGA-BRCA. (A) Gene–gene Pearson correlation matrix of the 36 angiogenesis genes across 1,227 breast cancer patients. Hierarchical clustering reveals structured positive correlation blocks, indicating coordinated co-expression submodules rather than a single homogeneous program. (B) Heatmap of log-transformed and Z-score–standardized gene expression values across patients. Distinct patient subgroups exhibit coordinated upregulation (red) and downregulation (blue) of angiogenesis genes, supporting a multi-dimensional latent expression architecture.

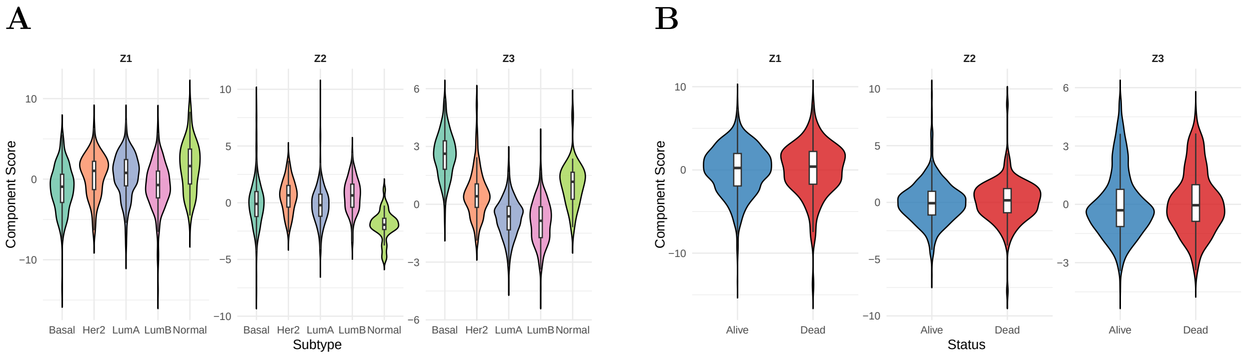

Fig 3. Latent angiogenesis components capture molecular subtype structure and survival-associated heterogeneity. (A) Distribution of latent components (Z_1-Z_3) across PAM50 breast cancer subtypes (Basal, Her2, LumA, LumB, Normal). The violin plots reveal subtype-specific shifts in latent expression patterns, indicating that angiogenesis-related transcriptional programs are not homogeneous but vary systematically across molecular subtypes. (B) Distribution of latent components stratified by survival status (Alive vs Dead). Subtle shifts in component distributions suggest that angiogenesis activity contributes modestly to survival differences, consistent with a diffuse but biologically meaningful signal rather than a single dominant prognostic axis.

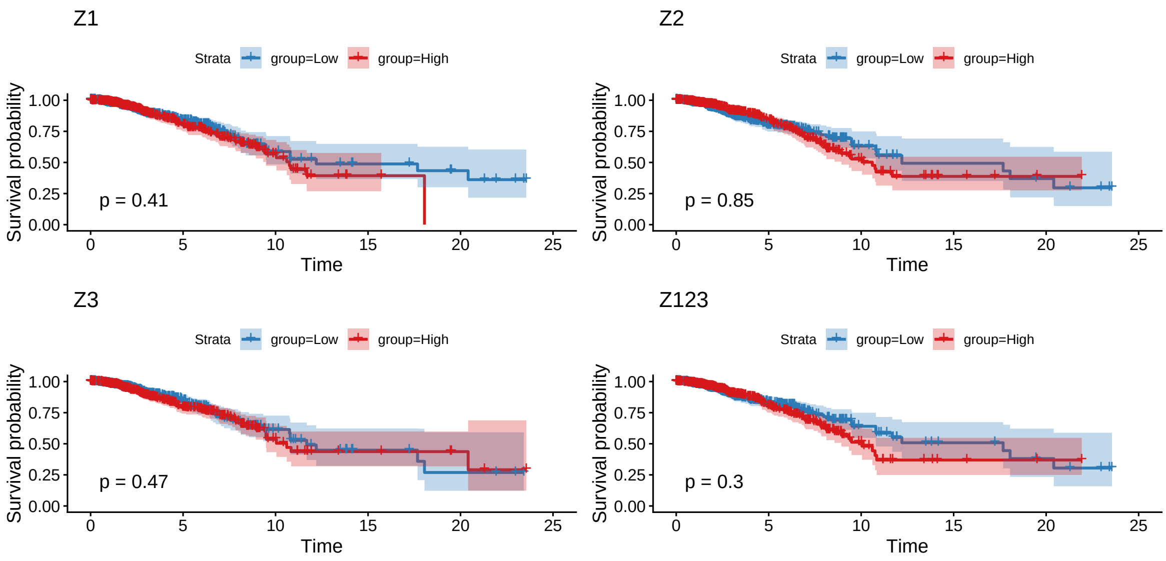

Fig 4. Unadjusted Kaplan–Meier survival curves for latent angiogenesis components. No significant survival differences were observed for Z_1-Z_3 or the combined score (Z_{123}), although a modest trend toward poorer survival is seen in the high Z_{123} group.

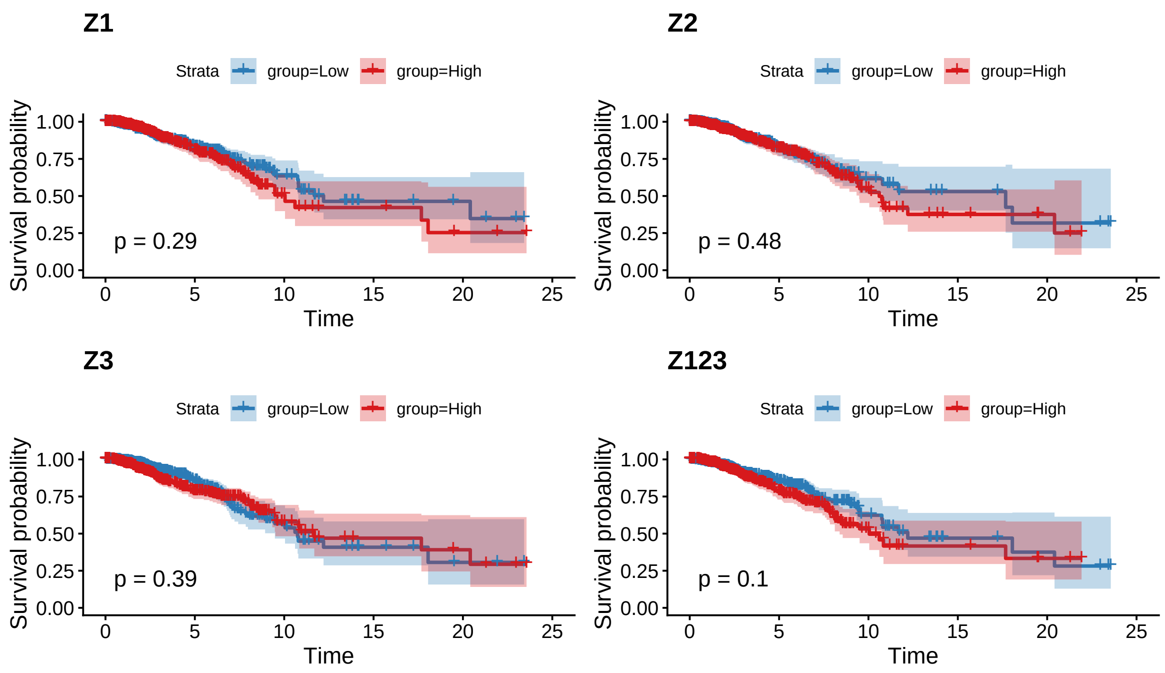

Fig 5. Kaplan–Meier survival analysis of latent angiogenesis components. Survival curves comparing high versus low groups (median split) for the first three latent components (Z_1-Z_3) and their combined score (Z_{123}). None of the individual components showed statistically significant survival differences (Z_1: $p = 0.29$, Z_2: $p = 0.48$, Z_3: $p = 0.39$). The combined angiogenesis score (Z_{123}) demonstrated a trend toward poorer survival in the high group ($p = 0.10$), suggesting that angiogenic activity is distributed across multiple latent dimensions rather than driven by a single dominant component. Shaded regions represent 95% confidence intervals.

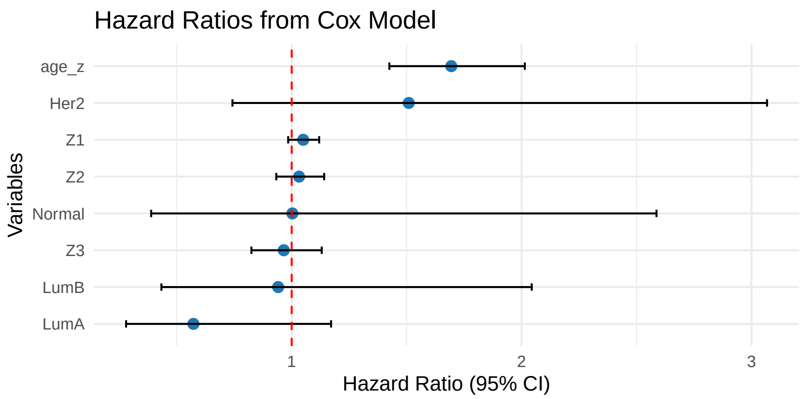

Fig 6. Adjusted hazard ratios for clinical covariates and angiogenesis gene program activity. Hazard ratios (HR) and 95% confidence intervals are shown for age at diagnosis and molecular subtypes in a multivariable Cox proportional hazards model. Points represent estimated hazard ratios and horizontal lines indicate 95% confidence intervals; the dashed vertical line at HR = 1 denotes no effect.

Preprint for the paper

Click here to access/download
Preprint PDF
Baidoo_Angiogenesis_Preprint.pdf
