# Supplementary material for: Multi-dimensional structure and subtype-specific heterogeneity of angiogenesis in breast cancer
Source: PLoS One. 2026 Oct 30;21(10):e0348390. doi: 10.1371/journal.pone.0348390 (PMC13722169; doi:10.1371/journal.pone.0348390)
Supplement: Supplementary file 2 [file pone.0348390.s003.pdf]

Multi-Dimensional Structure and Subtype-Specific Heterogeneity of Angiogenesis in Breast Cancer

Dear Editor and Reviewers,

We sincerely thank the Academic Editor and the reviewers for their careful evaluation of our manuscript and for their constructive suggestions. We have revised the manuscript extensively and believe the changes have substantially improved its clarity, methodological transparency, and scientific rigor. Reviewer comments are reproduced below in **bold**, followed by our responses.

Response to the Academic Editor and Reviewers

General Revisions

Several reviewers raised related concerns regarding methodological transparency, preprocessing procedures, latent component interpretation, model assumptions, reproducibility, and the clinical interpretation of the findings. In response, we have:

- Expanded the Introduction to better justify angiogenesis as a multidimensional biological process and clarify the methodological gap addressed by this study.
- Added detailed descriptions of RNA-seq preprocessing, TMM normalization, log-CPM transformation, gene filtering, and handling of missing data.
- Clarified the rationale for retaining the first three principal components based on variance explained, scree plot inspection, and biological interpretability.
- Explicitly stated that the combined angiogenesis score (Z_{123}) was exploratory and not a primary inferential endpoint.
- Added proportional hazards diagnostics, time-dependent sensitivity analyses, and complete-case analysis details.
- Expanded subtype-specific analyses and reported sample sizes, event counts, hazard ratios, confidence intervals, and p-values.
- Added Gene Ontology enrichment analyses for all retained latent components.
- Revised the Discussion to reduce overinterpretation of PCA loadings and emphasize the exploratory nature of biological interpretations.
- Added Data Availability and Ethics Approval sections.
- Updated references throughout the manuscript and incorporated additional methodological and biological citations.

- Revised the manuscript narrative to emphasize biological heterogeneity rather than prognostic prediction.

Reviewer 1

Abstract

Comment: The overall objective is not clearly stated.

Response: We thank the reviewer for this observation. The abstract has been revised to explicitly state the study objective, namely to characterize the multidimensional structure of angiogenesis and evaluate the prognostic relevance of its latent components for overall survival in breast cancer.

Comment: The conclusion lacks the answer to the topic in terms of survival.

Response: The abstract conclusion has been revised to explicitly state that none of the latent angiogenesis components demonstrated independent prognostic value for overall survival despite substantial biological heterogeneity.

Comment: Clarify the distinction between latent-component findings and the overall conclusion.

Response: The abstract has been revised to clearly separate component-level findings from the overall pathway-level conclusion. The revised text emphasizes that while angiogenesis exhibits biologically meaningful heterogeneity, it demonstrated limited independent prognostic relevance for overall survival.

Comment: Specify covariates used in adjusted models.

Response: The abstract now explicitly states that adjusted analyses included age and molecular subtype and clarifies the interpretation of subtype-specific analyses.

Introduction

Comment: Better justify latent multidimensional modeling.

Response: The Introduction has been expanded to explain why coordinated biological programs such as angiogenesis may not be adequately represented by single-gene analyses or single pathway scores. We further explain how PCA-based latent dimensions can reveal biologically distinct patterns of coordinated activity.

Comment: Clarify the knowledge gap.

Response: The Introduction now clearly states that relatively few studies have simultaneously modeled angiogenesis as a multidimensional latent process, evaluated subtype heterogeneity, and examined survival associations after accounting for clinical covariates.

Methods

Comment: Justify retention of three principal components and construction of Z_{123}.

Response: Additional justification has been added. Component retention was based on variance explained, scree plot inspection, and biological interpretability. We further clarify that Z_{123} was considered an exploratory descriptive summary rather than a primary inferential endpoint.

Comment: Clarify handling of missing data, censoring, stage, and model assumptions.

Response: We have expanded the Methods section to describe complete-case analysis, censoring definitions, handling of missing clinical data, event counts, molecular subtype classification, proportional hazards diagnostics using Schoenfeld residuals, and time-dependent sensitivity analyses.

Results

Comment: Reconcile Cox model and Kaplan–Meier findings.

Response: We now explicitly state that dichotomization may reduce statistical power and obscure continuous risk gradients captured by Cox proportional hazards models.

Comment: Provide more subtype-specific results.

Response: The Results section now includes additional subtype-specific Cox regression estimates, sample sizes, event counts, confidence intervals, and p-values.

Discussion

Comment: Avoid overinterpreting PCA loadings.

Response: We agree. The Discussion now emphasizes that interpretations based on PCA loadings are exploratory and hypothesis-generating rather than mechanistic.

Comment: Compare findings with previous angiogenesis signatures.

Response: The Discussion has been expanded to compare our findings with previous angiogenesis-related prognostic studies and to discuss differences in cohort composition, endpoints, subtype distributions, gene sets, and analytical strategies.

Conclusion

Comment: Avoid overstatement and provide future directions.

Response: The Conclusion has been revised to emphasize biological heterogeneity and limited prognostic value rather than strong prognostic utility. We now recommend future integration of angiogenesis with immune, stromal, hypoxia-related, and treatment-response features together with validation in independent cohorts.

Reviewer 2

Comment: Clarify preprocessing procedures.

Response: Detailed descriptions of TMM normalization, log-CPM transformation, gene filtering, quality control procedures, and handling of missing data have been added.

Comment: Justify PCA component retention and Z_{123}.

Response: Addressed as described above.

Comment: Justify median splits.

Response: We now explicitly note that median splits were used only for visualization and descriptive analyses. Primary inference was based on continuous Cox models.

Comment: Discuss weak model performance.

Response: The revised manuscript now discusses model discrimination and emphasizes that angiogenesis exhibited biological heterogeneity but limited independent prognostic value.

Reviewer 3

Comment: Positive evaluation.

Response: We thank the reviewer for the encouraging comments and careful assessment of the manuscript.

Reviewer 4

Response: We thank the reviewer for the thoughtful comments. In response:

- TCGA-BRCA is now defined in full in the abstract.
- The United States source of TCGA-BRCA data is now stated.
- Additional rationale for latent modeling has been added.
- Missing data handling, confounding variables, and event counts are now clearly described.
- Table 1 has been expanded and revised.
- Results sections have been rewritten to reduce discussion-like interpretation.
- Discussion language has been revised to reduce biological overstatement and improve balance.
- Data Availability and Ethics Approval statements have been added.
- Figure captions and titles have been revised for clarity and conciseness.

Reviewer 5

Response: We agree that the study's primary contribution lies in clarifying the multidimensional structure of angiogenesis rather than establishing a clinically actionable biomarker. Accordingly:

- The manuscript now emphasizes biological heterogeneity over prognostic prediction.
- The rationale for principal component retention has been expanded.
- The exploratory nature of Z_{123} has been clarified.
- Limitations regarding single-cohort design, residual confounding, and lack of external validation have been strengthened.
- Discussion of benchmarking against alternative angiogenesis scoring strategies has been added.

Reviewer 6

Comment 1: Were low-expression genes filtered?

Response: Yes. We have now explicitly stated that genes with counts per million (CPM) greater than 1 in a minimum proportion of samples were retained for analysis.

Comment 2: Were proportional hazards assumptions assessed?

Response: Yes. Proportional hazards assumptions were assessed using Schoenfeld residuals and global tests. These procedures are now described explicitly in the Methods section.

Reviewer 7

Response: We sincerely thank the reviewer for the positive comments and encouragement.

Reviewer 8

Response: We thank the reviewer for the detailed suggestions.

- We expanded biological interpretation for all retained latent components.
- Gene Ontology enrichment analyses were added for Z_1, Z_2, and Z_3.
- Subtype-specific Cox analyses were conducted and reported in detail.
- The manuscript now emphasizes biological heterogeneity rather than prognostic prediction.
- Figure captions have been clarified and standardized.
- Gene nomenclature has been reviewed for consistency.

Reviewer 9

Response: We appreciate the reviewer's emphasis on accessibility and interpretability.

- The Introduction has been expanded to provide additional biological and methodological context.
- Data acquisition and preprocessing sections were separated and expanded.
- PCA, latent structures, survival analysis, and related statistical concepts are now described more explicitly.
- The Results section now includes clearer descriptions of subgroup definitions, event counts, and key findings.
- The Discussion includes a more extensive comparison with previous angiogenesis literature.

We thank the Academic Editor and all reviewers for their constructive comments and believe that the revised manuscript has been substantially strengthened as a result.

Sincerely,
Dennis Baidoo
Division of Biostatistics, College of Public Health
The Ohio State University
Columbus, OH
