# Supplementary material for: Multi-dimensional structure and subtype-specific heterogeneity of angiogenesis in breast cancer
Source: PLoS One. 2026 Oct 30;21(10):e0348390. doi: 10.1371/journal.pone.0348390 (PMC13722169; doi:10.1371/journal.pone.0348390)
Supplement: Supplementary file 3 [file pone.0348390.s005.pdf]

Multi-Dimensional Structure and Subtype-Specific Heterogeneity of Angiogenesis in Breast Cancer

Dear Editor and Reviewers,

We sincerely thank the Academic Editor and the reviewers for their careful evaluation of our manuscript and for their constructive suggestions. We have revised the manuscript extensively and believe the changes have substantially improved its clarity, methodological transparency, and scientific rigor. Reviewer comments are reproduced below in **bold**, followed by our responses.

General Revisions

In response to the remaining comments from Reviewers 10, 11, and 13, we have:

- Removed the exploratory composite angiogenesis score (Z123) and all related analyses, tables, figures, and interpretations.
- Revised the manuscript to consistently interpret Z1–Z3 as PCA-derived latent transcriptional dimensions with biologically interpretable enrichment patterns.
- Removed language implying prognostic trends or prognostic utility when statistical significance was not observed.
- Removed the previous adjusted Kaplan–Meier presentation and clarified survival-analysis descriptions.
- Explicitly specified the low-expression filtering threshold (CPM > 1 in at least 10% of samples).
- Expanded the Discussion to better address the biological implications of subtype-specific heterogeneity and the limited prognostic relevance of angiogenesis alone.
- Strengthened the limitations section regarding the lack of external validation and the challenges associated with exploratory stage-adjusted analyses due to incomplete stage information.

Reviewer 2

Comment: The authors have adequately addressed the majority of my previous comments. The manuscript has been substantially improved in terms of methodological transparency, statistical reporting, and interpretation of the findings. Although some limitations remain (e.g., lack of external validation and limited justification for the exploratory Z123 score), these are appropriately acknowledged by the authors and do not preclude publication.

Response: We sincerely thank the reviewer for the encouraging assessment of the revised manuscript and for the constructive feedback provided throughout the review process. We appreciate the reviewer's recognition of the improvements in methodological transparency, statistical reporting, and interpretation of the findings. In the current revision, we have further strengthened the manuscript by removing the exploratory Z123 score entirely and expanding discussion of limitations related to external validation.

Reviewer 5

Comment: All comments have been addressed.

Response: We thank the reviewer for the positive evaluation and for the time and effort devoted to reviewing our manuscript.

Reviewer 10

Comment: The conceptual novelty and incremental contribution of the study appear limited, and the central prognostic hypothesis is not supported by the data.

Response: We thank the reviewer for this thoughtful assessment. We agree that the primary contribution of this study is the characterization of multidimensional angiogenesis-related transcriptional heterogeneity rather than the development of a clinically actionable prognostic biomarker. To better align the manuscript with this objective, we revised the manuscript throughout to emphasize biological heterogeneity and subtype-specific variation rather than prognostic prediction. We have also reduced language that could be interpreted as implying prognostic utility beyond that supported by the data.

Comment: The biological interpretation of the composite Z123 score remains uncertain.

Response: We appreciate the reviewer's concerns regarding the interpretation of the composite Z123 score. In response, we removed the exploratory Z123 score entirely from the manuscript, including all related analyses, tables, figures, and discussion. The revised manuscript now focuses exclusively on the three retained latent dimensions (Z1–Z3), which are interpreted as PCA-derived transcriptional axes supported by distinct enrichment patterns.

Comment: The description of Figure 6 as a Kaplan–Meier analysis performed while adjusting for clinical annotations is methodologically unclear.

Response: We agree with the reviewer that standard Kaplan–Meier curves are unadjusted estimators. To avoid ambiguity, we removed the previous adjusted Kaplan–Meier presentation and revised the survival-analysis section accordingly. The revised manuscript now clearly distinguishes between Kaplan–Meier visualization and Cox proportional hazards modeling.

Comment: The repeated emphasis on a trend toward poorer survival despite non-significant p-values should be reconsidered.

Response: We agree with the reviewer. We carefully reviewed the manuscript and removed language referring to "trends toward poorer survival" or similar interpretations when statistical significance was not observed. The revised manuscript now consistently reports non-significant findings using neutral and statistically appropriate language.

Comment: The low-expression filtering threshold is not fully specified.

Response: The Methods section has been revised to explicitly state the filtering criterion. Genes were retained if they exhibited counts per million (CPM) greater than 1 in at least 10% of samples.

Comment: The lack of independent validation in an external cohort such as METABRIC is an important limitation.

Response: We agree that external validation represents an important next step. We have strengthened the limitations section and discussion of future work to explicitly acknowledge the need for validation of the identified latent angiogenesis dimensions in independent cohorts such as METABRIC.

Comment: The multivariable model adjusts for age and molecular subtype but not tumor stage.

Response: We appreciate this observation. Tumor stage was evaluated descriptively and demonstrated only modest differences across latent angiogenesis groups. Additional exploratory stage-adjusted analyses resulted in substantial reductions in sample size and event count because of incomplete stage information, leading to unstable estimates for several stage categories. Consequently, we retained the primary model specification based on age and molecular subtype and expanded discussion of this limitation in the revised manuscript.

Reviewer 11

Comment: The author needs to justify the rationale for combining the first three PCA scores into a composite Z123 score and clarify its interpretation.

Response: We thank the reviewer for this helpful suggestion. To address concerns regarding interpretation of the composite score, we removed the exploratory Z123 measure entirely from the revised manuscript. The manuscript now consistently interprets Z1–Z3 as PCA-derived latent transcriptional dimensions supported by distinct enrichment profiles and biologically interpretable patterns. This revision allows the focus to remain on the multidimensional structure of angiogenesis without requiring interpretation of an aggregated composite score.

Comment: The authors can either provide additional evidence supporting the biological distinctness and robustness of the components or consistently frame them as PCA-derived transcriptional axes with biologically interpretable enrichment patterns.

Response: We agree with the reviewer and revised the manuscript accordingly. Throughout the manuscript, Z1–Z3 are now consistently described as PCA-derived latent transcriptional dimen-

sions. Interpretation is supported by gene loading patterns and Gene Ontology enrichment analyses, and we have avoided language suggesting that the components represent discrete biological entities.

Reviewer 13

Comment: The reviewer raises several questions regarding the biological interpretation of the findings, including the role of angiogenesis in disease progression, subtype-specific heterogeneity without corresponding survival differences, implications for anti-angiogenic therapies, integration with additional biological processes, and potential differences in metastatic disease.

Response: We thank the reviewer for these thoughtful questions. In response, we expanded the Discussion to more clearly address the biological implications of our findings. The revised Discussion emphasizes that angiogenesis-related transcriptional variation may represent an important dimension of tumor heterogeneity without necessarily functioning as an independent determinant of overall survival. We further discuss the possibility that prognostic discrimination may require integration with additional biological processes, including immune regulation, stromal remodeling, hypoxia-related pathways, and treatment-response mechanisms. Finally, we note that the current findings derive from primary TCGA-BRCA tumors and may differ in metastatic disease settings, highlighting an important direction for future investigation.

We thank the Academic Editor and all reviewers for their constructive comments and believe that the manuscript has been substantially strengthened as a result of these revisions.

Sincerely,

Dennis Baidoo
Division of Biostatistics
College of Public Health
The Ohio State University
Columbus, OH
