# Supplementary material for: Multi-dimensional structure and subtype-specific heterogeneity of angiogenesis in breast cancer
Source: PLoS One. 2026 Oct 30;21(10):e0348390. doi: 10.1371/journal.pone.0348390 (PMC13722169; doi:10.1371/journal.pone.0348390)
Supplement: Supplementary file 1 [file pone.0348390.s001.docx]

Title: Multi-Dimensional and Subtype-Specific Variation in Angiogenesis and Its Association with Survival in Breast Cancer

My comments:

**Abstract:**

1. Though the problem is clear, however the overall objective is not there and would improve the clarity of the research article.
2. The conclusion lacks the answer to the topic in terms of survival. It should be improved!
3. **Clearly distinguish between the statistically significant association of Z2 with survival and the overall conclusion that angiogenesis has limited independent prognostic value?**
   Since Z2 shows a significant Cox model association, while Kaplan–Meier and adjusted combined-score analyses are largely non-significant, the abstract should clarify whether the main message is “one latent axis has modest prognostic relevance” or “angiogenesis is not useful as a standalone biomarker.”
4. **Specify the key clinical covariates used in the adjusted Cox models and briefly state whether molecular subtype modified the angiogenesis–survival relationship?**
   The abstract mentions adjustment for clinical covariates and a Luminal A-specific effect, but readers would benefit from knowing upfront that age, stage/subtype, or other variables were considered and how subtype-specific findings influenced the interpretation.

**Introduction:**

1. **Can the introduction better justify why angiogenesis should be modeled as a multi-dimensional latent process rather than as a conventional pathway score or individual-gene signature?**
   The rationale is present, but it could be strengthened by explaining why PCA-based latent dimensions may reveal biologically meaningful heterogeneity that single-score approaches might miss.
2. **Identify the specific gap in prior breast cancer angiogenesis studies that this manuscript addresses?**
   The introduction discusses individual-gene and pathway-based prognostic studies, but it should more explicitly state whether the novelty lies in the latent variable framework, subtype-specific evaluation, adjustment for confounding, or interpretation of angiogenesis as a heterogeneous biological program.

**Methods:**

1. **Can the authors provide a stronger justification for retaining exactly three principal components and for constructing Z123 as the simple sum of Z1, Z2, and Z3?**
   Since the first three components explain 22.6%, 9.1%, and 7.7% of variance, the manuscript should explain whether retention was based on scree plot criteria, cumulative variance, biological interpretability, statistical association with survival, or another rule. The summation approach also needs justification because PCs are orthogonal and may represent biologically distinct or opposing processes.
2. **Clarify how missing clinical data, censoring, subtype classification, tumor stage, and proportional hazards assumptions were handled?**
   The manuscript states that Cox models were adjusted for clinical covariates, but a reader needs more detail on inclusion/exclusion criteria, missingness, event counts by subgroup, whether stage was included in final models, and whether Schoenfeld residuals or similar diagnostics were used.

**Results**

1. **Can the results better reconcile the significant continuous Cox association for Z2 with the non-significant Kaplan–Meier median-split analysis for Z2?**
   Z2 is significant in the Cox model, yet the median-based survival curves show no significant difference. The authors should explain whether this reflects loss of power from dichotomization, weak effect size, nonlinearity, confounding, or limited survival discrimination.
2. **Provide more quantitative detail for subtype-specific analyses, especially for Luminal A and Basal tumors?**
   The manuscript reports a modest Luminal A effect but not Basal tumors. It would be helpful to present hazard ratios, confidence intervals, p-values, sample sizes, and event counts for each subtype to support the claim that angiogenesis has subtype-dependent prognostic relevance.

**Discussion**

1. **Can the discussion more critically address the biological interpretation of Z2 and avoid overinterpreting PCA loadings as mechanistic pathways?**
   The interpretation of Z2 as a balance between extracellular matrix remodeling and regulatory/stabilizing pathways is interesting, but PCA is an unsupervised statistical method. The authors should discuss the extent to which this interpretation is exploratory and whether external validation or pathway enrichment supports it.
2. **Compare the findings with prior angiogenesis-related prognostic signatures in breast cancer?**
   Since the manuscript concludes that angiogenesis has limited standalone prognostic value, it should explain why this differs from or agrees with previous studies that reported stronger angiogenesis-related survival associations, including possible differences in cohorts, endpoints, gene sets, modeling strategies, and subtype composition.

**Conclusion**

1. **Avoid potential overstatement and align more tightly with the evidence presented?**
   The data support a modest, subtype-dependent, and mostly non-independent association. The conclusion should avoid implying that the study definitively establishes the role of angiogenesis in breast cancer survival without external validation.
2. **Include a clearer forward-looking statement about how angiogenesis should be incorporated into future prognostic models?**
   Rather than ending mainly with the limited standalone value of angiogenesis, the authors could specify whether future work should combine angiogenesis with immune, stromal, hypoxia, treatment-response, or tumor microenvironment features and validate the model in independent cohorts.
