# Supplementary material for: Multi-dimensional structure and subtype-specific heterogeneity of angiogenesis in breast cancer
Source: PLoS One. 2026 Oct 30;21(10):e0348390. doi: 10.1371/journal.pone.0348390 (PMC13722169; doi:10.1371/journal.pone.0348390)
Supplement: Supplementary file 2 [file pone.0348390.s004.docx]

**Recommendation**

The authors have addressed the majority of the concerns raised in the previous review. The revised manuscript provides substantially improved methodological transparency, including detailed preprocessing procedures, justification of PCA component retention, clarification of the exploratory nature of Z123, assessment of proportional hazards assumptions, and expanded subtype-specific analyses. The Discussion has also been appropriately revised to avoid overinterpretation of the prognostic findings.

One minor clarification remains regarding the precise proportion of samples used as the threshold for low-expression gene filtering. In addition, the lack of explicit batch correction should be acknowledged as a limitation, although the authors have transparently stated this issue.

Overall, the manuscript has been substantially improved and I believe the remaining issues are minor.

**أ**
