# Supplementary material for: Multi-dimensional structure and subtype-specific heterogeneity of angiogenesis in breast cancer
Source: PLoS One. 2026 Oct 30;21(10):e0348390. doi: 10.1371/journal.pone.0348390 (PMC13722169; doi:10.1371/journal.pone.0348390)
Supplement: Peer review history

# Peer review: Decision Letter 0

**DOI:** 10.1371/journal.pone.0348390.r001

**Authors:** UDAYAN BHATTACHARYA

18 May 2026

Dear Dr. Baidoo,

Thank you for submitting your manuscript to PLOS ONE. After careful consideration, we feel that it has merit but does not fully meet PLOS ONE’s publication criteria as it currently stands. Therefore, we invite you to submit a revised version of the manuscript that addresses the points raised during the review process.

Please submit your revised manuscript by Jul 02 2026 11:59PM. If you will need more time than this to complete your revisions, please reply to this message or contact the journal office at plosone@plos.org. When you're ready to submit your revision, log on to https://www.editorialmanager.com/pone/ and select the 'Submissions Needing Revision' folder to locate your manuscript file.

- A letter that responds to each point raised by the academic editor and reviewer(s). You should upload this letter as a separate file labeled 'Response to Reviewers'.
- A marked-up copy of your manuscript that highlights changes made to the original version. You should upload this as a separate file labeled 'Revised Manuscript with Track Changes'.
- An unmarked version of your revised paper without tracked changes. You should upload this as a separate file labeled 'Manuscript'.

If you would like to make changes to your financial disclosure, please include your updated statement in your cover letter. Guidelines for resubmitting your figure files are available below the reviewer comments at the end of this letter.

If applicable, we recommend that you deposit your laboratory protocols in protocols.io to enhance the reproducibility of your results. Protocols.io assigns your protocol its own identifier (DOI) so that it can be cited independently in the future. For instructions see: https://journals.plos.org/plosone/s/submission-guidelines#loc-laboratory-protocols. Additionally, PLOS ONE offers an option for publishing peer-reviewed Lab Protocol articles, which describe protocols hosted on protocols.io. Read more information on sharing protocols at https://plos.org/protocols?utm_medium=editorial-email&utm_source=authorletters&utm_campaign=protocols.

As the corresponding author, your ORCID iD is verified in the submission system and will appear in the published article. PLOS supports the use of ORCID, and we encourage all coauthors to register for an ORCID iD and use it as well. Please encourage your coauthors to verify their ORCID iD within the submission system before final acceptance, as unverified ORCID iDs will not appear in the published article. *Only*  the individual author can complete the verification step; PLOS staff *cannot*  verify ORCID iDs on behalf of authors.

We look forward to receiving your revised manuscript.

Kind regards,

UDAYAN BHATTACHARYA

Academic Editor

PLOS One

Journal Requirements:

When submitting your revision, we need you to address these additional requirements.

1. Please ensure that your manuscript meets PLOS ONE's style requirements, including those for file naming. The PLOS ONE style templates can be found at

https://journals.plos.org/plosone/s/file?id=wjVg/PLOSOne_formatting_sample_main_body.pdf and

https://journals.plos.org/plosone/s/file?id=ba62/PLOSOne_formatting_sample_title_authors_affiliations.pdf

2. Please note that PLOS One has specific guidelines on code sharing for submissions in which author-generated code underpins the findings in the manuscript. In these cases, we expect all author-generated code to be made available without restrictions upon publication of the work. Please review our guidelines at https://journals.plos.org/plosone/s/materials-and-software-sharing#loc-sharing-code and ensure that your code is shared in a way that follows best practice and facilitates reproducibility and reuse.

3. Thank you for stating the following in the Acknowledgments Section of your manuscript:

“The author received no specific funding for this work.”

We note that you have provided funding information that is not currently declared in your Funding Statement. However, funding information should not appear in the Acknowledgments section or other areas of your manuscript. We will only publish funding information present in the Funding Statement section of the online submission form.

Please remove any funding-related text from the manuscript and let us know how you would like to update your Funding Statement. Currently, your Funding Statement reads as follows:

“The author(s) received no specific funding for this work.”

Please include your amended statements within your cover letter; we will change the online submission form on your behalf.

4. Thank you for uploading your study's underlying data set. Unfortunately, the repository you have noted in your Data Availability statement does not qualify as an acceptable data repository according to PLOS's standards.

At this time, please upload the minimal data set necessary to replicate your study's findings to a stable, public repository (such as figshare or Dryad) and provide us with the relevant URLs, DOIs, or accession numbers that may be used to access these data. For a list of recommended repositories and additional information on PLOS standards for data deposition, please see https://journals.plos.org/plosone/s/recommended-repositories.

5. Please ensure that you refer to Figure 1 in your text as, if accepted, production will need this reference to link the reader to the figure.

6. If the reviewer comments include a recommendation to cite specific previously published works, please review and evaluate these publications to determine whether they are relevant and should be cited. There is no requirement to cite these works unless the editor has indicated otherwise.

Additional Editor Comments:

Manuscript Number: PONE-D-26-18657

Title: Multi-Dimensional and Subtype-Specific Variation in Angiogenesis and Its Association with Survival in Breast Cancer

Journal: PLOS ONE

Dear Dr. Baidoo,

Thank you for submitting your manuscript to PLOS ONE. Your work has been evaluated by a panel of reviewers, and their detailed comments are appended below.

As you will see, while the reviewers find the investigation into subtype-specific angiogenic variation and breast cancer survival to be highly relevant and promising, they have raised significant concerns that must be addressed before the manuscript can be accepted for publication. Therefore, we invite you to submit a revised version of the manuscript that thoroughly addresses these points.

In your revision, please pay particular attention to the major methodological and conceptual points highlighted by the reviewers, ensuring that data interpretations are robust and limitations are transparently discussed.

Please submit your revised manuscript and a detailed, point-by-point response to the reviewers' comments. Your response should explicitly outline the modifications made in response to each point raised.

Comments to the Author:

Reviewer 1: Minor Revision

Reviewer 2: Minor Revision

Reviewer 3: Accept

Reviewer 4: Minor Revision

Reviewer 5: Major Revision

Reviewer 6: Accept

Reviewer 7: Accept

Reviewer 8: Major Revision

Reviewer 9: Minor Revision

We look forward to receiving your revised manuscript.

Sincerely,

Dr. Udayan Bhattacharya

Academic Editor

PLOS ONE

[Note: HTML markup is below. Please do not edit.]

Reviewers' comments:

Reviewer's Responses to Questions

**Comments to the Author**

1. Is the manuscript technically sound, and do the data support the conclusions?

Reviewer #1: Yes

Reviewer #2: Yes

Reviewer #3: Yes

Reviewer #4: Partly

Reviewer #5: Yes

Reviewer #6: Yes

Reviewer #7: Yes

Reviewer #8: Yes

Reviewer #9: Yes

**********

2. Has the statistical analysis been performed appropriately and rigorously?

Reviewer #1: Yes

Reviewer #2: Yes

Reviewer #3: Yes

Reviewer #4: Yes

Reviewer #5: Yes

Reviewer #6: Yes

Reviewer #7: Yes

Reviewer #8: N/A

Reviewer #9: Yes

**********

3. Have the authors made all data underlying the findings in their manuscript fully available?

The PLOS Data policy

Reviewer #1: Yes

Reviewer #2: Yes

Reviewer #3: Yes

Reviewer #4: No

Reviewer #5: No

Reviewer #6: Yes

Reviewer #7: Yes

Reviewer #8: Yes

Reviewer #9: Yes

**********

4. Is the manuscript presented in an intelligible fashion and written in standard English?

Reviewer #1: Yes

Reviewer #2: Yes

Reviewer #3: Yes

Reviewer #4: Yes

Reviewer #5: Yes

Reviewer #6: Yes

Reviewer #7: Yes

Reviewer #8: Yes

Reviewer #9: Yes

**********

Reviewer #1: see my attached file comments. However, the paper is well written with minor grammatical errors. The scientific message is well captured and give an in-depth statistical analyses and database searches for genetic information related.

Reviewer #2: The manuscript addresses an important question regarding the prognostic relevance of angiogenesis in breast cancer using a pathway-level, latent variable approach. The idea of modeling angiogenesis as a multi-dimensional process rather than individual genes is conceptually sound and represents a meaningful contribution.

The study benefits from a relatively large sample size (TCGA-BRCA, n=1,227) and appropriate use of survival analysis methods. The results are generally consistent and clearly presented, particularly the identification of a specific latent component (Z2) associated with survival.

However, several methodological aspects require clarification. The preprocessing pipeline lacks sufficient detail (normalization, batch correction, quality control), and key analytical choices—such as retaining three PCA components and constructing the combined score (Z123)—are not adequately justified.

The use of median splits in Kaplan–Meier analysis should be reconsidered or justified, as it may reduce statistical power and obscure continuous relationships.

Model performance appears weak (C-index ≈ 0.545), and this limitation should be more explicitly acknowledged and discussed in terms of clinical relevance.

The biological interpretation of the Z2 component based on gene loadings is interesting but may be overinterpreted without external validation or functional analysis.

Subtype-specific findings (e.g., Luminal A) are potentially important but require deeper discussion and stronger support from the literature.

The manuscript would benefit from improved reporting of statistical assumptions (e.g., proportional hazards), handling of missing data, and inclusion of reproducibility details (software, code availability).

Several sections lack sufficient or up-to-date references, particularly for methodological choices (PCA, gene set scoring, median split) and biological interpretations.

Reviewer #3: The manuscript "Multi-Dimensional and Subtype-Specific Variation in Angiogenesis and Its Association

with Survival in Breast Cancer" was well articulated and the rationale was stated clearly. The methodology were clearly described and the statistical analyses were rightly applied. The findings were clearly presented and their implications sufficiently discussed. The limitations of the study were also duly outlined.

Reviewer #4: Review comments on “Multi-Dimensional and Subtype-Specific Variation in Angiogenesis and Its Association with Survival in Breast Cancer”

This study effectively leveraged on breast cancer data from the TCGA-BRCA cohort to evaluate the prognostic relevance of angiogenesis using RNA sequencing. A key strength is the adoption of a multi-dimensional latent modeling approach, which advances beyond single-gene or composite indices to capture coordinated gene expression patterns, reflecting biological complexity. Methodologically, the use of a large transcriptomic dataset (1,227 patients) and the integration of gene expression, molecular subtypes, and survival outcomes enhance statistical robustness and improve the potential generalizability of the findings within breast cancer research.

However, the study demonstrates limited prognostic value, as survival analyses revealed predominantly non-significant associations between latent angiogenesis components and survival outcomes (p-values largely >0.05), thereby weakening its translational relevance, although this remains an informative finding. Additionally, the lack of validation using independent datasets constrains the reproducibility and generalizability of the results. Furthermore, the absence of comparative benchmarking against established angiogenesis scoring systems limits the ability to evaluate the performance and added value of the proposed modeling approach.

On the Abstract

Write the full words for TCGA-BRCA before putting it in bracket and the name of the country/location for which the TCGA-BRCA 1,227 patients’ data was obtained from.

Introduction

Your introduction is generally okay, but your rationale for angiogenesis needs to sufficiently justify the choice of using latent modeling as a superior existing approach in Oncology.

Add 1 or 2 more points on current methodological gaps that predicate the study’s relevance.

Methods

Your methods lack a thorough description of the latent component derivation and clarity on the gene selection criteria. Also, there wasn’t any mention of how confounders, incomplete/missing data were handled

Results

Results write ups should show general pertinent points from your study analysis figures and not be written like discussion. Do address that across board in this section. You should work on table 1. Let it include demographic and clinical variables for the TCGA-BRCA cohorts like age, race, overall survival (in months or years), event rate etc. based on the Z 1, 2, 3 and 123 subgroup stratifications. Note that 518 +509=1,027 is 200 less your total of 1,227, so expatiate on that. Also, provide more details on the 36 angiogenesis-related genes sequenced using the RNA. Work seriously to summarize the write up for table 2 by making it briefer and more concise as a result overview. Put the specific p-values derived from your survival analysis for the Z 1, 3 and 123.

Reduce your heavy reliance on descriptive interpretation by boosting the work’s inferential robustness. Your figures suggest heterogeneity, but the clinical implications were weak. Try to work on that.

Discussion

Reduce most or remove those many bracketed result values written in this section and make the section more elaborate/interactive with consistent, contrasting or plausible explanations. Reduce the strong biological tone and overemphasize on that given that the interpretation from your statistical evidence was weak. Boost the section by stating why angiogenesis signals were not strongly predictive giving plausible reasons. Link such to potential confounders like treatment variability, demographics etc.

Conclusion

Your conclusion is okay, though you can make it better.

I didn’t see any statement on the modalities on how ethical clearance was obtained for your study data.

Work on your figure titles by making them brief and concise

Reviewer #5: This secondary analysis offers an incremental scientific contribution rather than a major conceptual advance. Its value lies in moving beyond the study of individual angiogenesis genes to examining angiogenesis as a coordinated, multi-dimensional transcriptional program within the large TCGA-BRCA cohort. In doing so, it suggests that prognostic information is concentrated in a specific latent component (Z2) rather than in overall angiogenesis pathway activity. That said, the practical impact remains limited because the principal finding is largely negative: angiogenesis shows only weak independent prognostic relevance, with poor discrimination (C-index 0.545) and minimal added value beyond established clinical factors such as age and molecular subtype.

Methodological framing is interesting, but the latent model is fairly basic and partly under-justified. PCA is a reasonable first-pass summary of correlated genes, and identifying subtype-dependent latent structure is useful. But the manuscript sometimes overstates this as a latent variable framework when the implementation is essentially unsupervised PCA plus Cox regression; the biological rationale for retaining exactly three PCs and for defining the combined score as a simple sum (Z123) is weak.

The survival analysis is informative, but the prognostic claims are limited by analytical issues. The finding that only Z2 associates with survival, while Kaplan–Meier curves are non-significant, is scientifically interesting because it suggests subtle continuous effects rather than clean patient stratification. However, there are internal presentation problems: adjusted Kaplan–Meier language is not correct in the usual sense, proportional hazards diagnostics are only asserted rather than shown, and subgroup claims (e.g., Luminal A effect) may be vulnerable to multiple-testing and low event counts.

The biological interpretation is plausible but remains shallow and not yet convincing as mechanism. The paper’s strongest scientific value is the suggestion that Z2 reflects a balance between extracellular matrix remodeling/pro-angiogenic signaling and stabilizing/regulatory pathways, which could explain why overall angiogenesis is not prognostic. Yet this interpretation is based only on PCA loadings, without deeper pathway, cell-type, stromal, vascular, or microenvironmental validation; thus, the biology remains speculative.

The study is clinically relevant, but its translational impact is weakened by design limitations and lack of validation. Using TCGA-BRCA with RNA-seq and clinical covariates is appropriate, and the paper appropriately concludes that angiogenesis alone is not a strong standalone biomarker. Still, the observational single-cohort design, incomplete treatment information, possible residual confounding, and no external validation make it hard to know whether the result is robust or clinically actionable.

Overall, this paper advances science modestly by clarifying the structure and limited prognostic role of angiogenesis in a large public cohort. However, the methodology is relatively simple for the claims made: PCA is useful but basic, the rationale for selecting three components and summing them into Z123 is not strongly justified, and there is no external validation. The study would be substantially strengthened by validating results in an independent dataset, comparing PCA-derived scores with established angiogenesis signatures, and incorporating richer clinical variables such as treatment and stage more systematically.

Reviewer #6: I appreciate the authors for performing such a nice piece of research work like, Integration of molecular subtype information strengthens the biological relevance of the angiogenesis analysis. I just want to clarify the two comments

1. Were low-expression genes filtered prior to downstream analysis? If yes, specify the filtering threshold.

2. Did the authors assess proportional hazard assumptions before performing survival analysis?

Reviewer #7: The manuscript is well written and illustrated with a valuable finding suggesting the factor of angiogenesis as a potent but not universal biomarker in breast cancer survival. The results and discussion are well presented in the manuscript with proper statistical analysis of the observations.

Reviewer #8: Major Revisions

1. In Table 2, while the second principal component (Z2) is statistically significant (p < 0.001), the Concordance Index (C-index) is only 0.545.

2. This value is only marginally better than random chance (0.5), which contradicts the claim of a strong "association with survival"

3. Requirement: You must explicitly discuss this limitation. The manuscript should pivot from presenting these components as "prognostic tools" to "biological insights into tumor heterogeneity".

4. The study identifies Z1, Z2, and Z3 through Principal Component Analysis (PCA) but primarily focuses on the gene loadings of Z2.

5. Requirement: To provide a truly "multi-dimensional" view, you must provide the biological context for Z1 and Z3 as well. Perform Gene Ontology (GO) or Pathway enrichment analysis to define what specific biological processes each axis represents (e.g., vessel sprouting vs. basement membrane stabilization).

6. Table 1 shows a highly significant difference in subtype distribution (p < 0.001), where the "Low Angiogenesis" group is enriched for Basal tumors.

7. Requirement: Since Basal-like tumors have notoriously poor prognosis, you must demonstrate that the survival associations for Z2 are not merely a reflection of these underlying subtype proportions. A subtype-stratified Cox model is necessary to confirm the independent effect of angiogenesis components.

Minor Revisions

1. Inconsistencies in P-values: In Table 1, ensure consistent decimal places for non-significant values (e.g., Age p=0.5 vs. Time p=0.4).

2. Gene Nomenclature: All gene symbols (e.g., ITGAV, OLR1, VCAN) must be consistently italicized throughout the text.

3. Clarity of Figures: In the Figure 3 caption, ensure you define the "latent scores" and the method of calculation for the violin plots to ensure reproducibility.

4. The current title is descriptive but perhaps too broad given the modest survival results.

5. Suggestion: Consider a title that emphasizes the "Heterogeneity" or "Deconstruction" of the angiogenesis program rather than its "Association with Survival," which the data suggests is diffuse and weak.

Reviewer #9: General Comment

The manuscript is generally well written, clearly structured, and addresses an important topic. The writing is concise and the flow of the manuscript allows the reader to follow the overall objectives and findings with relative ease.

However, the manuscript would benefit from the inclusion of additional explanatory detail, particularly in sections where statistical methods and concepts are introduced. At present, several key points rely heavily on references to previously published work without providing sufficient in-text explanation. While this approach may be appropriate for readers with a strong statistical background, it may limit accessibility for a broader audience.

Specifically, readers with limited statistical expertise may find it challenging to fully understand the methods and their implications, as the manuscript does not consistently provide simplified explanations or contextual descriptions of the referenced approaches. Including brief explanations of key statistical concepts, assumptions, and interpretations would greatly enhance clarity.

The specific areas where additional clarification and detail would be beneficial are indicated below.

Introduction

The authors indicated that prior studies have established that cancer progression is driven by groups of genes concerned with the regulation of process such as angiogenesis, immune evasion and metastasis (lines 17-21).

However, the authors do not provide a clear rationale for focusing specifically on angiogenesis over other relevant biological processes. Including this justification in the Introduction would provide important context and help guide the reader’s understanding of the study’s focus.

In addition, it would be beneficial for the authors to elaborate on the choice of methodology, specifically explaining why the selected approach was used in preference to alternative methods. Providing this reasoning would strengthen the manuscript and improve its clarity.

Materials and Methods

The authors should consider separating the “Data Source Preprocessing” subsections to allow each to be described in sufficient detail and with greater clarity (lines 41-56). This would improve the overall structure and make the information easier to follow. Relevant articles by Lyu, RB. (2020) Survival Analysis of Lung Cancer Patients from TCGA Cohort. Advances in Lung Cancer and Wu and Fang (2022) Stacked Autoencoder Based Multi-Omics Data Integration for Cancer Survival Prediction, among others, could be used as useful guides to strengthen and refine these sections.

A similar issue is observed in other subsections, such as “Exploratory Latent Structure Assessment” where principal component analysis (PCA) and Cox proportional hazards regression were introduced without a clear explanation of what the process entails or its relevance and importance within the study (lines 57-76). Providing this context would significantly improve reader understanding.

In addition, the “Gene Module Definition, Data Structure and Notation, Multi-Factor Latent Gene Module Model, and Survival Analysis” sections would benefit from a brief introductory explanation of the analytical approaches and data sets before presenting the analyses themselves (lines 77-121). This would help orient readers, particularly those without a strong statistical background. The suggested papers could also be used as examples to provide context and support clearer explanations across these sections.

Expanding these subsections with additional detail would help to better explain the processes involved and make the content more accessible to a broader readership.

Results

The “Baseline Characteristics” section does not provide sufficient detail on how the low and high angiogenesis groups were defined, and this should be clearly explained (lines 123-138). In addition, it would be helpful to include key demographic and clinical characteristics, such as age range, sex distribution, treatment, and outcome data (e.g., the number of individuals who survived and/or died within each group, and this information should be included in Figure 3B).

Similarly, the “Latent Structure of Angiogenesis Gene Expression” section would benefit from a clearer synthesis of the findings presented in the Figure 2 (lines171-186). Providing a brief summary of the key gene clusters with shared expression patterns, along with specific examples of upregulated and downregulated genes, would help to guide the reader’s interpretation and highlight the most important relationships.

The Results section should clearly state the number of participants included in each analysis.

Discussion

The authors stated that the findings provide a potential explanation for inconsistent results in prior studies that have treated angiogenesis as a single pathway (lines 295-297). However, they do not discuss the findings from these prior studies.

Final Thought

Overall, strengthening the explanatory components would make the manuscript more accessible, improve reader comprehension, and increase its impact across a wider audience.

**********

what does this mean?). If published, this will include your full peer review and any attached files.

If you choose “no”, your identity will remain anonymous but your review may still be made public.

**Do you want your identity to be public for this peer review?**  For information about this choice, including consent withdrawal, please see our Privacy Policy

Reviewer #1: **Yes:**  Jean Baptiste Niyibizi

Reviewer #2: **Yes:**  Associate Prof. Shimaa El-Sayed Rashad

Reviewer #3: No

Reviewer #4: **Yes:**  Martin Zang Pam

Reviewer #5: No

Reviewer #6: **Yes:**  Dr.P.Priya Dharsini

Reviewer #7: No

Reviewer #8: No

Reviewer #9: No

**********

[NOTE: If reviewer comments were submitted as an attachment file, they will be attached to this email and accessible via the submission site. Please log into your account, locate the manuscript record, and check for the action link "View Attachments". If this link does not appear, there are no attachment files.]

To ensure your figures meet our technical requirements, please review our figure guidelines: https://journals.plos.org/plosone/s/figures

You may also use PLOS’s free figure tool, NAAS, to help you prepare publication quality figures: https://journals.plos.org/plosone/s/figures#loc-tools-for-figure-preparation.

NAAS will assess whether your figures meet our technical requirements by comparing each figure against our figure specifications.

Attachment Submitted filename: Review PLOS ONE JBN.docx

Attachment Submitted filename: PONE-D-26-18657_reviewer.pdf

# Author comment: Author response to Decision Letter 1

**DOI:** 10.1371/journal.pone.0348390.r002

21 Jul 2026

Title: Multi-Dimensional Structure and Subtype-Specific Heterogeneity of Angiogenesis in Breast Cancer

Dear Dr. Bhattacharya,

We sincerely thank the Academic Editor and all reviewers for their careful evaluation of our manuscript and their constructive comments. We have revised the manuscript extensively in response to the concerns raised. Major revisions include expansion of the methodological description, clarification of preprocessing procedures and model assumptions, justification of latent component derivation, additional subtype-specific analyses, incorporation of Gene Ontology enrichment analysis, improved reporting of survival analyses, broader discussion of limitations and biological interpretation, inclusion of Data Availability and Ethics statements, and substantial revisions throughout the manuscript to improve clarity, transparency, and reproducibility.

Detailed responses are provided below.

Reviewer #1

We thank the reviewer for the thoughtful and constructive comments. In response, we revised the Abstract to more clearly state the study objective and to distinguish between component-level findings and the overall conclusion regarding angiogenesis as a prognostic biomarker. We clarified the role of clinical covariates in the adjusted Cox models and strengthened the discussion of subtype-specific findings.

The Introduction was revised to better justify modeling angiogenesis as a multidimensional latent process and to more explicitly define the methodological and biological gap addressed by the study. Additional detail was added regarding retention of the first three principal components, construction of the exploratory angiogenesis score (Z123), handling of missing data, censoring, molecular subtype classification, and proportional hazards diagnostics.

The Results section was expanded to better explain the relationship between continuous Cox models and Kaplan–Meier analyses and now includes more detailed subtype-specific results. The Discussion was revised to reduce overinterpretation of PCA loadings, emphasize the exploratory nature of biological interpretations, and provide broader comparison with previously published angiogenesis-related prognostic studies. The Conclusion was rewritten to avoid overstatement and provide clearer future directions involving integration with additional tumor microenvironment features and independent validation.

Reviewer #2

We appreciate the reviewer’s positive assessment of the study and the helpful suggestions regarding methodological transparency. The preprocessing workflow was expanded substantially and now includes detailed descriptions of RNA-seq normalization using TMM, log-CPM transformation, low-expression gene filtering, quality-control procedures, and complete-case analysis. Additional justification was provided for retaining three principal components and for the exploratory use of Z123.

The manuscript now explicitly discusses the limitations of median-split Kaplan–Meier analyses and clarifies that primary inference relied on continuous Cox proportional hazards models. We expanded reporting of proportional hazards diagnostics, censoring procedures, and model assumptions. Additional methodological references were added throughout the manuscript.

The Results and Discussion were revised to more carefully interpret model discrimination and emphasize that the primary contribution of the study lies in characterizing angiogenesis-related biological heterogeneity rather than establishing a clinically useful prognostic tool.

Reviewer #3

We thank the reviewer for the positive comments regarding the clarity of the rationale, methodology, statistical analyses, presentation of results, discussion, and limitations. No specific methodological revisions were requested; however, the manuscript has been strengthened further through revisions made in response to comments from the other reviewers.

Reviewer #4

We appreciate the reviewer’s detailed comments and suggestions. The Abstract was revised to provide the full name of TCGA-BRCA and identify the source population. Additional rationale was added to justify the latent modeling framework and to better explain existing methodological gaps in angiogenesis research.

The Methods section was substantially expanded to provide greater detail regarding latent component derivation, gene-selection procedures, handling of confounders, missing data, and proportional hazards assumptions. Table 1 was revised and expanded to improve presentation of demographic and clinical variables across angiogenesis strata. Results text was rewritten to focus on objective presentation of findings and reduce discussion-style interpretation. Additional details concerning the Hallmark angiogenesis gene set and the biological interpretation of latent components were incorporated.

The Discussion was revised extensively to reduce biological overstatement, improve balance, and provide more nuanced discussion of weak prognostic findings and potential explanations, including subtype heterogeneity and residual confounding. Dedicated Data Availability and Ethics Approval sections have also been added. Figure titles and captions were revised to improve clarity and conciseness.

Reviewer #5

We thank the reviewer for recognizing the value of the pathway-level perspective and for the detailed methodological suggestions. The manuscript was revised to more clearly present the study as an investigation of biological heterogeneity rather than as a prognostic prediction framework. The rationale for retaining three principal components was expanded and the exploratory nature of Z123 was clarified throughout the Methods and Discussion.

The Discussion now acknowledges limitations associated with PCA, lack of external validation, and absence of benchmarking against alternative angiogenesis scoring systems. Additional discussion was added regarding differences between latent-variable approaches and established angiogenesis-related prognostic signatures. Limitations associated with single-cohort analyses, residual confounding, treatment variability, and generalizability are now discussed in greater detail.

Reviewer #6

We thank the reviewer for the positive assessment of the work and for the two specific methodological questions. The Methods now explicitly state that low-expression genes were filtered prior to downstream analysis using counts-per-million thresholds. The manuscript also now clearly describes assessment of proportional hazards assumptions using Schoenfeld residuals, global tests, and time-dependent sensitivity analyses.

Reviewer #7

We are grateful for the reviewer’s positive assessment of the manuscript and appreciation of the study’s integration of molecular subtype information and statistical methodology. We thank the reviewer for the encouraging comments.

Reviewer #8

We appreciate the reviewer’s detailed and thoughtful suggestions. In response, Gene Ontology enrichment analyses were performed and incorporated for all retained latent components, allowing biological interpretation of Z1, Z2, and Z3. The Results and Discussion now emphasize biological heterogeneity rather than strong prognostic prediction.

Additional subtype-specific Cox proportional hazards analyses were conducted and reported, including hazard ratios, confidence intervals, sample sizes, and event counts, demonstrating that the observed associations were not solely attributable to differences in subtype composition. Figure captions were expanded to improve reproducibility and clarity, and nomenclature was reviewed for consistency. The manuscript title and discussion emphasis were revised to better reflect the limited independent prognostic value of angiogenesis.

Reviewer #9

We thank the reviewer for the constructive comments regarding accessibility and methodological explanation. The Introduction was expanded to provide additional biological and methodological rationale for modeling angiogenesis using latent dimensions and to better explain why angiogenesis was selected as the focal pathway.

Several sections within the Methods were reorganized and expanded to improve readability and accessibility, including separate descriptions of data acquisition, preprocessing, latent structure analysis, PCA methodology, and survival analysis. Additional explanations were provided for key statistical concepts and analytical assumptions. The Results section now includes clearer descriptions of subgroup definitions, event counts, latent component distributions, and interpretation of key findings. The Discussion was expanded to more thoroughly compare the present findings with prior angiogenesis-related prognostic studies.

Closing Remarks

We sincerely thank the Academic Editor and all reviewers for their valuable comments and recommendations. We believe that the extensive revisions have substantially strengthened the manuscript, improving methodological transparency, interpretability, reproducibility, and overall scientific rigor. We appreciate the opportunity to revise our work and respectfully submit this revised manuscript for further consideration.

Sincerely,

Dennis Baidoo

Division of Biostatistics, College of Public Health

The Ohio State University

Columbus, OH

baidoo.20@osu.edu

Attachment Submitted filename: Response_to_Reviewers.pdf

# Peer review: Decision Letter 1

**DOI:** 10.1371/journal.pone.0348390.r003

**Authors:** UDAYAN BHATTACHARYA

25 Aug 2026

Dear Dr. Baidoo,

plosone@plos.org. When you're ready to submit your revision, log on to https://www.editorialmanager.com/pone/ and select the 'Submissions Needing Revision' folder to locate your manuscript file.

- A letter that responds to each point raised by the academic editor and reviewer(s). You should upload this letter as a separate file labeled 'Response to Reviewers'.
- A marked-up copy of your manuscript that highlights changes made to the original version. You should upload this as a separate file labeled 'Revised Manuscript with Track Changes'.
- An unmarked version of your revised paper without tracked changes. You should upload this as a separate file labeled 'Manuscript'.

If you would like to make changes to your financial disclosure, please include your updated statement in your cover letter. Guidelines for resubmitting your figure files are available below the reviewer comments at the end of this letter.

If applicable, we recommend that you deposit your laboratory protocols in protocols.io to enhance the reproducibility of your results. Protocols.io assigns your protocol its own identifier (DOI) so that it can be cited independently in the future. For instructions see: https://journals.plos.org/plosone/s/submission-guidelines#loc-laboratory-protocols. Additionally, PLOS One offers an option for publishing peer-reviewed Lab Protocol articles, which describe protocols hosted on protocols.io. Read more information on sharing protocols at https://plos.org/protocols?utm_medium=editorial-email&utm_source=authorletters&utm_campaign=protocols.

As the corresponding author, your ORCID iD is verified in the submission system and will appear in the published article. PLOS supports the use of ORCID, and we encourage all coauthors to register for an ORCID iD and use it as well. Please encourage your coauthors to verify their ORCID iD within the submission system before final acceptance, as unverified ORCID iDs will not appear in the published article. *Only*  the individual author can complete the verification step; PLOS staff *cannot*  verify ORCID iDs on behalf of authors.

We look forward to receiving your revised manuscript.

Kind regards,

UDAYAN BHATTACHARYA

Academic Editor

PLOS One

Journal Requirements:

If the reviewer comments include a recommendation to cite specific previously published works, please review and evaluate these publications to determine whether they are relevant and should be cited. There is no requirement to cite these works unless the editor has indicated otherwise.

Please review your reference list to ensure that it is complete and correct. If you have cited papers that have been retracted, please include the rationale for doing so in the manuscript text, or remove these references and replace them with relevant current references. Any changes to the reference list should be mentioned in the rebuttal letter that accompanies your revised manuscript. If you need to cite a retracted article, indicate the article’s retracted status in the References list and also include a citation and full reference for the retraction notice.

Additional Editor Comments:

Dear Dr. Baidoo,

Thank you for submitting the revised manuscript "Multi-Dimensional Structure and Subtype-Specific Heterogeneity of Angiogenesis in Breast Cancer" (Manuscript Number: PONE-D-26-18657R1) to PLOS ONE.

We have now received reports from the reviewers who evaluated Revision 1 of your work. As you will see from their comments appended below, the reviewers are generally positive about the improvements made to the paper. However, a few minor concerns and clarification requests remain that need to be addressed before a final decision can be reached.

Therefore, we invite you to submit a minor revision of your manuscript.

Key Points to Address in Your Revision:

Point-by-Point Response: Include a detailed cover letter providing a point-by-point response to each reviewer comment, clearly indicating where changes were made in the text.

Tracked Changes: Upload both a marked-up copy of your manuscript highlighting changes (using tracked changes or highlighting) and a clean version.

Reviewer Comments: Carefully address the specific feedback provided by the reviewers below, particularly resolving remaining technical clarifications and typographical refinements.

Please ensure your revised manuscript and response document are uploaded through the Editorial Manager portal by the specified deadline.

We look forward to receiving your revised manuscript.

Sincerely,

Dr. Udayan Bhattacharya

Academic Editor, PLOS ONE

[Note: HTML markup is below. Please do not edit.]

Reviewers' comments:

Reviewer's Responses to Questions

**Comments to the Author**

Reviewer #2: All comments have been addressed

Reviewer #5: All comments have been addressed

Reviewer #10: (No Response)

Reviewer #11: (No Response)

Reviewer #12: All comments have been addressed

Reviewer #13: (No Response)

**********

2. Is the manuscript technically sound, and do the data support the conclusions?

Reviewer #2: Yes

Reviewer #5: Yes

Reviewer #10: Partly

Reviewer #11: Partly

Reviewer #12: Yes

Reviewer #13: Yes

**********

3. Has the statistical analysis been performed appropriately and rigorously?

Reviewer #2: Yes

Reviewer #5: Yes

Reviewer #10: Yes

Reviewer #11: Yes

Reviewer #12: Yes

Reviewer #13: Yes

**********

4. Have the authors made all data underlying the findings in their manuscript fully available?

The PLOS Data policy

Reviewer #2: Yes

Reviewer #5: Yes

Reviewer #10: Yes

Reviewer #11: Yes

Reviewer #12: Yes

Reviewer #13: Yes

**********

5. Is the manuscript presented in an intelligible fashion and written in standard English?

Reviewer #2: Yes

Reviewer #5: Yes

Reviewer #10: Yes

Reviewer #11: Yes

Reviewer #12: Yes

Reviewer #13: Yes

**********

Reviewer #2: The authors have adequately addressed the majority of my previous comments. The manuscript has been substantially improved in terms of methodological transparency, statistical reporting, and interpretation of the findings. Although some limitations remain (e.g., lack of external validation and limited justification for the exploratory Z123 score), these are appropriately acknowledged by the authors and do not preclude publication. I recommend acceptance after minor revision, or acceptance if the journal considers these remaining issues sufficiently addressed.

Reviewer #5: (No Response)

Reviewer #10: Manuscript: Multi-Dimensional Structure and Subtype-Specific Heterogeneity of Angiogenesis in Breast Cancer (PONE-D-26-18657R1)

This study presents a comprehensive characterization of angiogenesis-related transcriptional heterogeneity in breast cancer using RNA-sequencing and clinical data from 1,096 patients in the TCGA-BRCA cohort. The authors identified three latent components by applying PCA to a predefined 36-gene Hallmark angiogenesis set and integrated these components with functional enrichment, PAM50 molecular subtype, and overall survival analyses. A key strength of the study lies in treating angiogenesis as a multidimensional transcriptional program rather than reducing it to individual genes or a single aggregate risk score. The marked differences in latent-component distributions across molecular subtypes support the presence of biologically relevant heterogeneity. However, none of the retained components showed a statistically significant independent association with overall survival, and the combined angiogenesis score did not provide clear incremental prognostic value. Overall, the manuscript is substantially more convincing as a descriptive characterization of subtype-associated angiogenesis biology than as evidence of a meaningful prognostic or translational advance.

The main concern is that the conceptual novelty of the study and its incremental contribution to the existing literature appear limited. PCA-based characterization of angiogenesis-related expression heterogeneity and prognostic evaluation of angiogenesis-associated gene programs have been explored previously, and the principal positive finding here—subtype-dependent variation in angiogenesis-related transcriptional activity—extends rather than fundamentally changes the current understanding of breast cancer heterogeneity. At the same time, the central prognostic hypothesis is not supported by the data: Z1, Z2, and Z3 all show hazard ratios close to unity with non-significant associations, and Z123 does not significantly improve prognostic modeling beyond clinical covariates. Several methodological issues further weaken the interpretation. The primary multivariable model adjusts for age and molecular subtype but not for tumor stage, despite claims regarding prognostic information beyond established clinical factors. The reported C-index of 0.676 cannot be clearly attributed to the angiogenesis components, as the manuscript does not demonstrate a meaningful increase in discrimination beyond the clinical model. Subtype-specific conclusions are also broader than the detailed results presented; estimates are reported primarily for Z2, while formal component-by-subtype interaction tests are not clearly presented. Furthermore, several subtype strata contain very few events, substantially limiting statistical power and making strong conclusions regarding the absence of subtype-specific effects difficult to justify.

Additional concerns include the description of Figure 6 as a Kaplan–Meier analysis performed “while adjusting for clinical annotations,” which is methodologically unclear because standard Kaplan–Meier estimation is unadjusted, as well as the repeated emphasis on a “trend toward poorer survival” for Z123 despite p>0.05. The biological interpretation of a score constructed by summing orthogonal principal components also remains uncertain, and the low-expression filtering threshold is not fully specified. In addition, the lack of independent validation of the PCA-derived structure in an external cohort such as METABRIC is an important limitation. More importantly, these concerns are not limited to reporting or presentation. Even if several methodological issues were addressed in revision, the study would still lack a clearly novel positive biological finding, a statistically supported prognostic signal, or demonstrated incremental clinical utility. In my view, the present work therefore represents a largely confirmatory and descriptive extension of existing knowledge rather than a sufficiently substantive advance in biological, prognostic, or translational understanding. For these reasons, I do not believe that revision alone would adequately address the fundamental limitations in novelty and scientific impact, and I recommend rejection of the manuscript.

Reviewer #11: This manuscript presents an interesting analysis of angiogenesis-related transcriptional variation in breast cancer using TCGA-BRCA.

The study employed a distinguishing approach of integrating transcriptomic and clinical information, and an attempt to move beyond conventional single-score representations of angiogenesis. The manuscript was well written, had an excellent study design and detailed statistical analysis.

One major concern is the need to clarify the interpretation of PCA-derived components. The author needs to justify the rationale to combine the first three PCA scores into a composite Z123 score and hence the interpretation.

The authors can either provide additional evidence supporting the biological distinctness and robustness of the components or consistently frame them as PCA-derived transcriptional axes with biologically interpretable enrichment patterns.

Reviewer #12: (No Response)

Reviewer #13: Given that the study identified no independent prognostic significance for survival, does this finding suggest that angiogenesis functions as a driver of disease progression, or does it instead represent a secondary or epiphenomenal feature of the tumor microenvironment?

How should the substantial variation observed in the distribution of latent components across molecular subtypes inform our understanding of intrinsic breast cancer subtypes, given that these differences do not appear to translate into differential survival outcomes?

In light of the finding that age constituted a strong predictor of overall survival whereas the angiogenesis score did not, what inferences can be drawn regarding the relative contribution of systemic versus tumor-intrinsic factors to patient outcomes?

Does the absence of a statistically significant association within individual molecular subtypes suggest that anti-angiogenic therapies should be stratified according to specific latent dimensions rather than overall angiogenesis expression levels?

Given the conclusion that angiogenesis alone is insufficient as a standalone prognostic biomarker, what additional multi-dimensional biological processes might be integrated to enhance prognostic discrimination?

Considering that the findings derive from the TCGA-BRCA cohort, to what extent might these results differ if the analysis were extended to metastatic disease rather than confined to primary invasive carcinoma samples?

**********

what does this mean?). If published, this will include your full peer review and any attached files.

If you choose “no”, your identity will remain anonymous but your review may still be made public.

**Do you want your identity to be public for this peer review?**  For information about this choice, including consent withdrawal, please see our Privacy Policy

Reviewer #2: **Yes:**  Dr. Shimaa E. Rashad

Reviewer #5: No

Reviewer #10: No

Reviewer #11: No

Reviewer #12: No

Reviewer #13: No

**********

[NOTE: If reviewer comments were submitted as an attachment file, they will be attached to this email and accessible via the submission site. Please log into your account, locate the manuscript record, and check for the action link "View Attachments". If this link does not appear, there are no attachment files.]

To ensure your figures meet our technical requirements, please review our figure guidelines: https://journals.plos.org/plosone/s/figures

You may also use PLOS’s free figure tool, NAAS, to help you prepare publication quality figures: https://journals.plos.org/plosone/s/figures#loc-tools-for-figure-preparation.

NAAS will assess whether your figures meet our technical requirements by comparing each figure against our figure specifications.

Attachment Submitted filename: New Microsoft Word Document.docx

# Author comment: Author response to Decision Letter 2

**DOI:** 10.1371/journal.pone.0348390.r004

12 Oct 2026

Dear Editor and Reviewers,

We sincerely thank the Academic Editor and the reviewers for their careful evaluation of our manuscript and for their constructive suggestions. We have revised the manuscript extensively and believe the changes have substantially improved its clarity, methodological transparency, and scientific rigor. Reviewer comments are reproduced below, followed by our responses.

General Revisions

In response to the remaining comments from Reviewers 10, 11, and 13, we have:

• Removed the exploratory composite angiogenesis score (Z123) and all related analyses, tables, figures, and interpretations.

• Revised the manuscript to consistently interpret Z1–Z3 as PCA-derived latent transcriptional dimensions with biologically interpretable enrichment patterns.

• Removed language implying prognostic trends or prognostic utility when statistical significance was not observed.

• Removed the previous adjusted Kaplan–Meier presentation and clarified survival-analysis descriptions.

• Explicitly specified the low-expression filtering threshold (CPM > 1 in at least 10% of samples).

• Expanded the Discussion to better address the biological implications of subtype-specific heterogeneity and the limited prognostic relevance of angiogenesis alone.

• Strengthened the limitations section regarding the lack of external validation and the challenges associated with exploratory stage-adjusted analyses due to incomplete stage information.

Reviewer 2

Comment: The authors have adequately addressed the majority of my previous comments. The manuscript has been substantially improved in terms of methodological transparency, statistical reporting, and interpretation of the findings. Although some limitations remain (e.g., lack of external validation and limited justification for the exploratory Z123 score), these are appropriately acknowledged by the authors and do not preclude publication.

Response: We sincerely thank the reviewer for the encouraging assessment of the revised manuscript and for the constructive feedback provided throughout the review process. We appreciate the reviewer’s recognition of the improvements in methodological transparency, statistical reporting, and interpretation of the findings. In the current revision, we have further strengthened the manuscript by removing the exploratory Z123 score entirely and expanding discussion of

limitations related to external validation.

Reviewer 5

Comment: All comments have been addressed.

Response: We thank the reviewer for the positive evaluation and for the time and effort

devoted to reviewing our manuscript.

Reviewer 10

Comment: The conceptual novelty and incremental contribution of the study appear limited, and the central prognostic hypothesis is not supported by the data.

Response: We thank the reviewer for this thoughtful assessment. We agree that the primary contribution of this study is the characterization of multidimensional angiogenesis-related transcriptional heterogeneity rather than the development of a clinically actionable prognostic biomarker. To better align the manuscript with this objective, we revised the manuscript throughout to emphasize biological heterogeneity and subtype-specific variation rather than prognostic prediction.

We have also reduced language that could be interpreted as implying prognostic utility beyond that supported by the data.

Comment: The biological interpretation of the composite Z123 score remains uncertain. Response: We appreciate the reviewer’s concerns regarding the interpretation of the composite Z123 score. In response, we removed the exploratory Z123 score entirely from the manuscript, including all related analyses, tables, figures, and discussion. The revised manuscript now focuses exclusively on the three retained latent dimensions (Z1–Z3), which are interpreted as PCA-derived

transcriptional axes supported by distinct enrichment patterns. Comment: The description of Figure 6 as a Kaplan–Meier analysis performed while adjusting for clinical annotations is methodologically unclear.

Response: We agree with the reviewer that standard Kaplan–Meier curves are unadjusted estimators. To avoid ambiguity, we removed the previous adjusted Kaplan–Meier presentation and revised the survival-analysis section accordingly. The revised manuscript now clearly distinguishes between Kaplan–Meier visualization and Cox proportional hazards modeling. Comment: The repeated emphasis on a trend toward poorer survival despite non-significant p-values should be reconsidered. Response: We agree with the reviewer. We carefully reviewed the manuscript and removed language referring to “trends toward poorer survival” or similar interpretations when statistical significance was not observed. The revised manuscript now consistently reports non-significant

findings using neutral and statistically appropriate language. Comment: The low-expression filtering threshold is not fully specified.

Response: The Methods section has been revised to explicitly state the filtering criterion. Genes were retained if they exhibited counts per million (CPM) greater than 1 in at least 10% of samples. Comment: The lack of independent validation in an external cohort such as METABRIC is

an important limitation. Response: We agree that external validation represents an important next step. We have

strengthened the limitations section and discussion of future work to explicitly acknowledge the need for validation of the identified latent angiogenesis dimensions in independent cohorts such as METABRIC.

Comment: The multivariable model adjusts for age and molecular subtype but not tumor stage. Response: We appreciate this observation. Tumor stage was evaluated descriptively and demonstrated only modest differences across latent angiogenesis groups. Additional exploratory stage-adjusted analyses resulted in substantial reductions in sample size and event count because of incomplete stage information, leading to unstable estimates for several stage categories. Consequently, we retained the primary model specification based on age and molecular subtype and expanded discussion of this limitation in the revised manuscript.

Reviewer 11

Comment: The author needs to justify the rationale for combining the first three PCA scores into a composite Z123 score and clarify its interpretation.

Response: We thank the reviewer for this helpful suggestion. To address concerns regarding interpretation of the composite score, we removed the exploratory Z123 measure entirely from the revised manuscript. The manuscript now consistently interprets Z1–Z3 as PCA-derived latent transcriptional dimensions supported by distinct enrichment profiles and biologically interpretable patterns. This revision allows the focus to remain on the multidimensional structure of angiogenesis without requiring interpretation of an aggregated composite score. Comment: The authors can either provide additional evidence supporting the biological dis-

tinctness and robustness of the components or consistently frame them as PCA-derived transcriptional axes with biologically interpretable enrichment patterns.

Response: We agree with the reviewer and revised the manuscript accordingly. Throughout the manuscript, Z1–Z3 are now consistently described as PCA-derived latent transcriptional dimensions. Interpretation is supported by gene loading patterns and Gene Ontology enrichment analyses, and we have avoided language suggesting that the components represent discrete biological entities.

Reviewer 13

Comment: The reviewer raises several questions regarding the biological interpretation of the findings, including the role of angiogenesis in disease progression, subtype-specific heterogeneity without corresponding survival differences, implications for anti-angiogenic therapies, integration with additional biological processes, and potential differences in metastatic disease. Response: We thank the reviewer for these thoughtful questions. In response, we expanded the Discussion to more clearly address the biological implications of our findings. The revised Discussion emphasizes that angiogenesis-related transcriptional variation may represent an important dimension of tumor heterogeneity without necessarily functioning as an independent determinant of overall survival. We further discuss the possibility that prognostic discrimination may require integration with additional biological processes, including immune regulation, stromal remodeling, hypoxia-related pathways, and treatment-response mechanisms. Finally, we note that the current findings derive from primary TCGA-BRCA tumors and may differ in metastatic disease settings,

highlighting an important direction for future investigation. We thank the Academic Editor and all reviewers for their constructive comments and believe that the manuscript has been substantially strengthened as a result of these revisions.

Sincerely,

Dennis Baidoo

Division of Biostatistics

College of Public Health

The Ohio State University

Columbus, OH

Attachment Submitted filename: Response_to_Reviewers-Baidoo.pdf

# Editor report: Decision Letter 2

**DOI:** 10.1371/journal.pone.0348390.r005

**Authors:** UDAYAN BHATTACHARYA

15 Oct 2026

Multi-Dimensional Structure and Subtype-Specific Heterogeneity of Angiogenesis in Breast Cancer

PONE-D-26-18657R2

Dear Dr. Baidoo,

We’re pleased to inform you that your manuscript has been judged scientifically suitable for publication and will be formally accepted for publication once it meets all outstanding technical requirements.

Within one week, you’ll receive an e-mail detailing the required amendments. When these have been addressed, you’ll receive a formal acceptance letter and your manuscript will be scheduled for publication.

An invoice will be generated when your article is formally accepted. Please note, if your institution has a publishing partnership with PLOS and your article meets the relevant criteria, all or part of your publication costs will be covered. Please make sure your user information is up-to-date by logging into Editorial Manager at Editorial Manager® and clicking the ‘Update My Information' link at the top of the page. For questions related to billing, please contact billing support.

If your institution or institutions have a press office, please notify them about your upcoming paper to help maximize its impact. If they’ll be preparing press materials, please inform our press team as soon as possible -- no later than 48 hours after receiving the formal acceptance. Your manuscript will remain under strict press embargo until 2 pm Eastern Time on the date of publication. For more information, please contact onepress@plos.org.

Kind regards,

UDAYAN BHATTACHARYA

Academic Editor

PLOS One

Additional Editor Comments (optional):

Dear Dr. Baidoo,

It is a pleasure to inform you that your manuscript entitled "Multi-Dimensional Structure and Subtype-Specific Heterogeneity of Angiogenesis in Breast Cancer" has been formally accepted for publication in PLOS ONE.

We appreciate the thorough revisions you and your co-authors have undertaken in response to the reviewers' comments. The changes have significantly strengthened the manuscript, and we are confident that this work will make a valuable contribution to the field.

Reviewers' comments:

# Editor report: Acceptance letter

**DOI:** 10.1371/journal.pone.0348390.r006

**Authors:** UDAYAN BHATTACHARYA

PONE-D-26-18657R2

PLOS One

Dear Dr. Baidoo,

I'm pleased to inform you that your manuscript has been deemed suitable for publication in PLOS One. Congratulations! Your manuscript is now being handed over to our production team.

At this stage, our production department will prepare your paper for publication. This includes ensuring the following:

* All references, tables, and figures are properly cited

* All relevant supporting information is included in the manuscript submission,

* There are no issues that prevent the paper from being properly typeset

You will receive further instructions from the production team, including instructions on how to review your proof when it is ready. Please keep in mind that we are working through a large volume of accepted articles, so please give us a few days to review your paper and let you know the next and final steps.

Lastly, if your institution or institutions have a press office, please let them know about your upcoming paper now to help maximize its impact. If they'll be preparing press materials, please inform our press team within the next 48 hours. Your manuscript will remain under strict press embargo until 2 pm Eastern Time on the date of publication. For more information, please contact onepress@plos.org.

You will receive an invoice from PLOS for your publication fee after your manuscript has reached the completed accept phase. If you receive an email requesting payment before acceptance or for any other service, this may be a phishing scheme. Learn how to identify phishing emails and protect your accounts at https://explore.plos.org/phishing.

If we can help with anything else, please email us at customercare@plos.org.

Thank you for submitting your work to PLOS One and supporting open access.

Kind regards,

PLOS One Editorial Office Staff

on behalf of

Dr. UDAYAN BHATTACHARYA

Academic Editor

PLOS One
